# Compositional Analysis of Flatworm Genomes Shows Strong Codon Usage Biases Across All Classes

**DOI:** 10.3389/fgene.2019.00771

**Published:** 2019-09-05

**Authors:** Guillermo Lamolle, Santiago Fontenla, Gastón Rijo, Jose F. Tort, Pablo Smircich

**Affiliations:** ^1^Departamento de Genética, Facultad de Medicina, Universidad de la Republica, UDELAR, Montevideo, Uruguay; ^2^Departamento de Genómica, Instituto de Investigaciones Biológicas Clemente Estable, IIBCE, MEC, Montevideo, Uruguay; ^3^Laboratorio de Interacciones Moleculares, Facultad de Ciencias, Universidad de la Republica, UDELAR, Montevideo, Uruguay

**Keywords:** flatworms, GC content, synonymous codons, codon usage, non-synonymous substitutions, amino acid usage, mutation, selection

## Abstract

In the present work, we performed a comparative genome-wide analysis of 22 species representative of the main clades and lifestyles of the phylum Platyhelminthes. We selected a set of 700 orthologous genes conserved in all species, measuring changes in GC content, codon, and amino acid usage in orthologous positions. Values of 3^rd^ codon position GC spanned over a wide range, allowing to discriminate two distinctive clusters within freshwater turbellarians, Cestodes and Trematodes respectively. Furthermore, a hierarchical clustering of codon usage data differs remarkably from the phylogenetic tree. Additionally, we detected a synonymous codon usage bias that was more dramatic in extreme GC-poor or GC-rich genomes, i.e., GC-poor Schistosomes preferred to use AT-rich terminated synonymous codons, while GC-rich *M. lignano* showed the opposite behavior. Interestingly, these biases impacted the amino acidic usage, with preferred amino acids encoded by codons following the GC content trend. These are associated with non-synonymous substitutions at orthologous positions. The detailed analysis of the synonymous and non-synonymous changes provides evidence for a two-hit mechanism where both mutation and selection forces drive the diverse coding strategies of flatworms.

## Introduction

The phylum Platyhelminthes with more than 30,000 species is one of the major phyla of invertebrate animals containing an enormous diversity of life forms that had colonized very diverse niches (Caira and Littlewood, 2013). Almost three quarters of the flatworms are parasitic and belong to the Neodermata, a monophyletic clade characterized by a syncytial tegument and the presence of diverse specialized organs to attach to hosts like suckers and hooks. The Neodermata comprise three classes: the Monogenea (primarily external parasites of cold-blooded aquatic vertebrates), the Cestoda (obligate endoparasites of vertebrates), and the Trematoda (endoparasites of vertebrates as adults, with intermediate stages endoparasitic in other invertebrates, mainly mollusks) (Caira and Littlewood, 2013). Besides the parasitic Neodermatans, an enormous diversity of species occurs in seas, rivers, and lakes and on all continental land masses comprising one of the successful phyla of invertebrates (Collins, 2017). A few species exist as either commensals or occasional parasites of invertebrates, but most of them are free-living predator species forming a single paraphyletic group collectively referred to as “turbellarians” (Caira and Littlewood, 2013). Studies based on rRNA (Larsson and Jondelius, 2008; Laumer and Giribet, 2014) and transcriptomic data (Egger et al., 2015; Laumer et al., 2015) showed that the phylum Platyhelminthes split early into two clades: the ancestral Catenulida and the Rhabditophora, which includes several free-living orders and the Neodermatans (**Table 1**). Taxonomically, the Macrostomorpha was placed as the earliest diverging Rhabditophoran linage and the order Tricladida, which contains the model organism *Schmidtea mediterranea*, as part of the later-evolved “turbellarians.”

**Table 1:** List of species analyzed and their GC content.FL, Free-living; PR, parasitic; G.GC, genomic GC percentage; T.GC, transcript (CDS) GC percentage.

Class	Subclass/order	Species	Abbreviation	Habitat	G.GC	T.GC
CATENULIDA		*Stenostomum leucops*	Sleu	FL		54.0
RHABDITOPHORA	Macrostomorpha	*Macrostomum lignano*	Mlig	FL	45.9	58.7
RHABDITOPHORA	Lecithoepitheliata	*Geocentrophora applanata*	Gapp	FL		37.8
RHABDITOPHORA	Polycladida	*Prostheceraeus vittatus*	Pvit	FL		46.4
RHABDITOPHORA	Neodalyellida/Rhabdocoela	*Rhynchomesostoma rostratum*	Rros	FL		40.1
RHABDITOPHORA	Dalyellioida/Fecampiida	*Kronborgia amphipodicola*	Kamp	PR		40.3
RHABDITOPHORA	Seriata/Proseriata	*Monocelis fusca*	Mfus	FL		40.8
RHABDITOPHORA	Seriata/Bothrioplanida	*Botrhioplana semperi*	Bsem	FL		53.0
RHABDITOPHORA	Seriata/Tricladida	*Schmidtea mediterranea*	Smed	FL	29.9	36.6
MONOGENEA	Monopisthocotylea/Gyrodactylidea	*Gyrodactilus salaris*	Gsal	PR	33.9	43.3
MONOGENEA	Polyopisthocotylea/Polystomatidea	*Protopolystoma xenopodis*	Pxen	PR	37.7	50.2
TREMATODA	Digenea/Strigeidida	*Schistosoma mansoni*	Sman	PR	35.5	36.0
TREMATODA	Digenea/Strigeidida	*Schistosoma japonicum*	Sjap	PR	34.1	36.0
TREMATODA	Digenea/Strigeidida	*Trichobilharzia regenti*	Treg	PR	37.4	37.2
TREMATODA	Digenea/Plagiorchiida	*Fasciola hepatica*	Fhep	PR	44.1	47.8
TREMATODA	Digenea/Opisthorchiida	*Clonorchis sinensis*	Csin	PR	44.0	48.4
TREMATODA	Digenea/Opisthorchiida	*Opistorchis viverrini*	Oviv	PR	43.8	48.5
CESTODA	Eucestoda/Diphyllobothriidea	*Schistocephalus solidus*	Ssol	PR	43.0	51.9
CESTODA	Eucestoda/Cyclophyllidea	*Mesocestoides corti*	Mcor	PR	36.7	51.4
CESTODA	Eucestoda/Cyclophyllidea	*Hymenolepis diminuta*	Hdim	PR	35.2	44.2
CESTODA	Eucestoda/Cyclophyllidea	*Echinococcus granulosus*	Egra	PR	41.9	50.0
CESTODA	Eucestoda/Cyclophyllidea	*Echinococcus multilocularis*	Emul	PR	42.2	49.9

*FL, free-living; PR, parasitic; G.GC, genomic GC percentage; T.GC, transcript (CDS) GC percentage.*

The huge diversity of flatworm’s life forms seems to be paralleled at genomic level. The recent publication of several genomic assemblies of the phylum (most of them corresponding to parasitic Neodermatans) has revealed a wide genomic diversity. For example, genome sizes range from 67 or 104 Mbases in the monogenean *Gyrodactilus salaris* or the cestode *Hydatigera taeniaeformis*, respectively, to 1,200 Mbases in the trematode *Fasciola hepatica* (Coghlan et al., 2019). Interestingly, this variation has little correlation with gene set completeness among genomes and is mostly due to non-coding elements, including repetitive and non-repetitive elements, with repeat content ranging from less than 4% in the smallest genomes of cestodes to 68% in *Fasciola hepatica*. Additionally, guanine and cytosine (GC) contents are very diverse from 28% in the planaria *S. mediterranea* and 33% in Monogenea *Gyrodactylus salaris*, to more than 45% in *M. lignano* and the food-borne trematodes (FBT) *F. hepatica*, *C. sinensis*, and *O. volvulus* (Coghlan et al., 2019).

We wondered if these large variations in genomic composition and structure could be correlated with the morphological and ecological diversity. It is well known that genomic GC content determines codon usage across species (Bernardi and Bernardi, 1985; Plotkin and Kudla, 2011) and the use of alternative synonymous codons is a non-random process (Sharp et al., 2010; Plotkin and Kudla, 2011). Due to the degeneracy of the genetic code, most amino acids, with the exceptions of methionine and tryptophan, are encoded by more than one codon. Codon usage bias (CUB) is a phenomenon where synonymous codons are not used with equal frequencies in coding DNA. It has been suggested that codon usage bias is the result of an equilibrium between mutational bias and natural selection and that natural selection could be acting in presumably highly expressed genes (Sharp et al., 2010; Plotkin and Kudla, 2011). Besides the effect at synonymous codon usage, it has been shown that strong GC bias could lead to changes in amino acid frequencies (Behura and Severson, 2013; Li et al., 2015). While this has not been explored widely in flatworms, several advances have been made in nematodes (Cutter et al., 2006; Mitreva et al., 2006; Mazumder et al., 2017a; Mazumder et al., 2017b). It is not clear yet how genomic GC differences could be influencing the codon usage and amino acid composition of proteins in Platyhelminthes and if these variations correlate with the ecological and physiological diversity in the phylum.

First reports of flatworm codon usage predated the genomic era and were based on a low representative number of sequences in Schistosomes and *Echinococcus*. Heterogeneity was evidenced since Schistosomes preferred A+T-rich codons, while *Echinococcus* favored GC3-rich codons (Meadows and Simpson, 1989; Alvarez et al., 1993; Kalinna and McManus, 1994; Milho and Tracy, 1995). Further analysis in larger sets of genes showed that codon bias was not uniformly distributed between genes introducing the possibility of isochores (regions that differ in GC content) in the genomes of flatworms (Ellis and Morrison, 1995; Ellis et al., 1995). In agreement, a more recent compositional analysis of the *S. mansoni* genome reported an isochore-like organization (Lamolle et al., 2016). Early studies analyzing the forces behind codon bias found evidences of both mutational pressure (Musto et al., 1998) in *S. mansoni* and selection (Fernandez et al., 2001) in *Echinococcus* spp. as preponderant forces. More recently, studies on *S. haematobium* and *S. japonicum* confirmed a major role of natural selection in shaping the codon usage bias in these species (Mazumder et al., 2017a; Mazumder et al., 2017b). Several studies analyzed codon usage on the available genomes and transcriptomes of *Taenidae* species showing weak codon bias and a higher GC3 in highly expressed genes explained by combined mutational and selection forces (Chen et al., 2013; Yang et al., 2014; Yang et al., 2015; Huang et al., 2017). A more preponderant contribution of selection shaping codon usage was identified in a comparative analysis in *Echinococcus* species (Maldonado et al., 2018). While these studies highlight that platyhelminthes are compositionally varied, they are focused just in the schistosomes and tapeworms. We took advantage of the wide array of transcriptomes and genomes now available to extend the study to a phylum-wide analysis of codon usage patterns, as a proxy of the molecular organization of flatworm genomes. We performed a comparative analysis at the genomic level of 22 species representative of the main clades and lifestyles of the phylum Platyhelminthes. Within these species, we picked a set of 700 orthologous gene groups conserved across the 22 species and measured changes in GC content, codon, and amino acid usage in orthologous positions. We found a class independent-wide diversity in codon and amino acid usages. Based on the study of orthologous positions in selected pairs of species with diverse GC content, we provide evidence of a combined contribution of mutational forces and selection that enforced synonymous codon usage bias and differential amino acid usage.

## Methods

### Data Acquisition

Genomic and coding sequences of 22 flatworm species were used in this work. To ease data visualization, a four-letter code was used to name the species (**Table 1**). Genomic and transcriptomic data of Mlig, Smed, Gsal, Pxen, Csin, Oviv, Fhep, Treg, Sjap, Sman, Mcor, Hdim, Egra, Emul, and Ssol were obtained from the public repository Wormbase parasite (Howe et al., 2017) (https://parasite.wormbase.org/). Transcriptomic data on Sleu, Gapp, Pvit, Rros, Mfus, Kamp, and Bsem were generated by Laumer et al. (2015) and downloaded from the public repository Data Dryad (doi:10.5061/dryad.622q4).

### Orthologues Determination

In-house Perl and Bash scripts that implemented a BLASTp best reciprocal hit strategy were used to identify a core of orthologous genes. An e-value cutoff of 1e-5 was used to define significant hits. The restrictive method produced one orthologue gene per species. A total of 700 orthologous groups were detected in all 22 species, and these sequences were used for the analysis, adding to more than 8 million codons analyzed (8.242.428).

Expression data for *S. mansoni* in reads per kilobase per million (RPKM) were taken from the study of Protasio et al. (2012). Expression data were available for 696 of the 700 *S. mansoni* orthologs. Expression data for the adult stages of *F. hepatica*, *E. granulosus*, *H. diminuta*, *S. mediterranea*, and *M. lignano* were downloaded from WormBase Parasite (Howe et al., 2017).

### Gene Alignment and Phylogenetic Tree

Each group of 22 orthologous sequences were translated with an in-house Perl script and aligned individually with Mafft (Katoh and Standley, 2013). Individual alignments were concatenated into a unique alignment. This alignment was used to build a phylogenetic tree with PhyML (Guindon et al., 2010). PhyML was run with the following options: -b -4, to calculate statistical branch support, -s BEST, for tree topology estimation, -m LG, to indicate the model substitution matrix, and -o tl, for tree topology and branch length optimization.

For the hierarchical clustering based on GC content of synonymous codons (RSCU), several clusters were built using hclust from R Stats package with different option settings (R Core Team, 2019). A final consensus cluster was made with the Ape package (Paradis et al., 2004), which retained the most frequent groupings.

### Codon Usage Analysis

Codon usage and compositional analyzes were done in R with the package seqinR (Charif and Lobry, 2007). Correlations between frequencies of each codon and GC3 were represented as heatmap with the R “Corrplot” package (Wei and Simko, 2017). In-house R scripts were used to evaluate significance of changes in frequencies between high- and low-expressed gene sets and defined preferred codons. A codon was considered “preferred” if its frequency (RSCU) significantly increases in a set of high-expression genes, compared with a low-expression set, regardless of whether it becomes the main codon for that amino acid or not. Correspondence analysis (COA) was performed in R.

### Neutrality and Effective Number of Codons Plots

Neutrality plots (Sueoka, 1988) (GC3 vs GC12) of the 22 species were used to evaluate the relationship among the three codon positions. Additionally, a unique plot showing general GC3–GC12 for all species was calculated by using a concatenated super gene for each species.

The effective number of codons (ENC) is used to quantify the variation in codon usage, ranging from 20 (when only one codon per amino acid is used) to 61 (when all possible codons are used). GC3 vs ENC charts are useful to estimate selection contribution to CUB. Expected values of ENC based on mutation pressure generate a bell curve, so in these charts, the points that fall directly on the curve represent genes with neutral evolution, while the points under the curve suggest action of natural selection (Wright, 1990).

### Amino Acid and Codon Substitutions Matrices

From the amino acid alignments of each COG, the sites that had gaps in one or more sequences were eliminated. Degapped COGs with less than 35 amino acids were eliminated. The resulting sequences were then concatenated, generating a “super-peptide” (without gaps) for each species. Then, the amino acid changes between each pair of species were counted (with a homemade R script), creating 20 by 20 substitution matrices. Each value of the matrix (A_Z,X_) represents how many times amino acid Z is present in one species, while amino acid X is present in the corresponding orthologous position in the other species, being, therefore, an asymmetric matrix. The diagonal of the matrix represents the unchanged sites, while the sum of the remaining values in each column or row represents the total substitutions for each amino acid. To test for deviations in the amino acid usage between species, the total count for each amino acid in the species of a pair was calculated, and the average was considered as expected value to perform chi-square tests (**Figure 5**). For each reciprocal changes in the matrix (A_Z,X,_ B_X,Z_), a chi-square test was performed considering the average of the counts as expected value. For simplicity of analysis, we focused in three comparisons between species with different global GC: cestodes (Hdim and Egra), trematodes (Sman and Fhep), and free-living species (Smed and Mlig). The last comparison involved the two more divergent species in GC content.

Based on the back-translation of the alignments, we generated a 61 × 61 (stop codons deleted) codon substitution matrix for the six selected species following similar procedures as the ones described in the previous section.

## Results

### Global GC Composition Varies Across Diverse Flatworm Taxa

As a first approach to analyze if there is a GC compositional difference in the phylum Platyhelminthes, we inspected the difference in the global genomic and transcriptomic G+C content. At first glance, it was clear that there is no correlation between genomic and transcriptomic GC, so it was not possible to use transcript GC to infer genomic GC. In most of the species, transcripts were GC richer than global genomic GC with the only exception of *T. regenti* (**Table 1**). However, while Schistosomatidae species show almost no difference in GC content between the overall genome and the coding region, Cestodes transcripts, for example, were on average 9.7% GC richer than all the genome considered together.

### GC Composition Varies Across Diverse Flatworm Taxa

**Figure 1:**
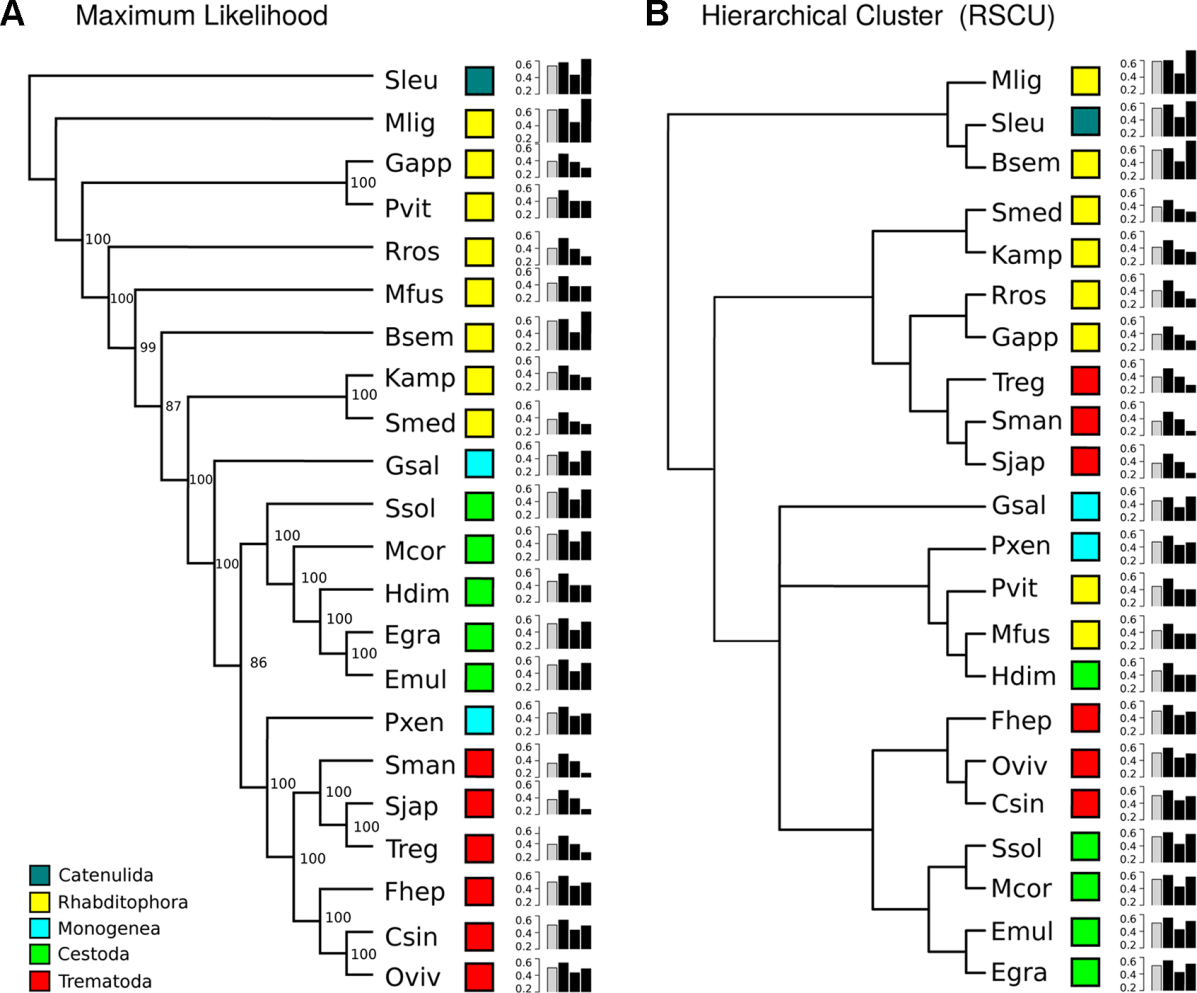
GC composition variation across Platyhelminthes. **(A)** Maximum likelihood phylogenetic tree built with a set of 700 orthologue genes. Light blue, green, and red boxes correspond to the Neodermata species; blue and yellow boxes correspond to “turbellaria” species (see abbreviations in **Table 1**). SH-like support values are indicated for each node. **(B)** Hierarchical clustering based on GC content of relative synonymous codons (RSCU). Histograms reflect overall GC content in all orthologues (gray bar) and by codon position (black bars). GC3 is the most variable between species.

To further analyze the GC composition in the coding region, we searched for a set of orthologous conserved genes in the available genomes and transcriptomes. Based on a best reciprocal hit BLAST search, we selected 700 orthologous genes present in the 22 species. A maximum likelihood tree confirmed that the orthologous groups strongly represented the accepted phylogeny of the species analyzed (organisms form the same group cluster together, with the only exception of the two Monogenea, which represent two distinct subclasses) (**Figure 1A**). Since GC varies across the different species, we calculated the relative synonymous codon usage (RSCU) and the GC values by codon position in this set of conserved genes. The clustering of the species based on the relative synonymous codon usage (RSCU) data showed an important reorganization respect to the phylogenetic tree (**Figure 1B**). Three main clusters were clearly appreciable: the first with high GC3 (with free-living species), the second with low GC3 values (including other free-living species and the schistosomatids), and a third with intermediate GC3 values. GC2 was the less variable between groups (0.42, 0.38, and 0.41 on average in groups 1, 2, and 3, respectively). Additionally, we noticed three subgroups within group 3: one that had lower GC1–2 than the rest but had high GC3 composed only by the monogean *G. salaris*; the cluster of *P. xenopodis*, *P. vittatus*, *M. fusca*, and the cestode *H. diminuta* that had lower GC3; and the subgroup composed by trematodes (*F. hepatica*, *O. viverrini*, *C. sinensis*) and cestodes (*S. solidus*, *M. corti*, *E. multilocularis*, and *E. granulosus*) that had higher G1-3 compared with other species of the group. This shows that global synonymous codon usage varies widely across the phylum.

**Figure 2:**
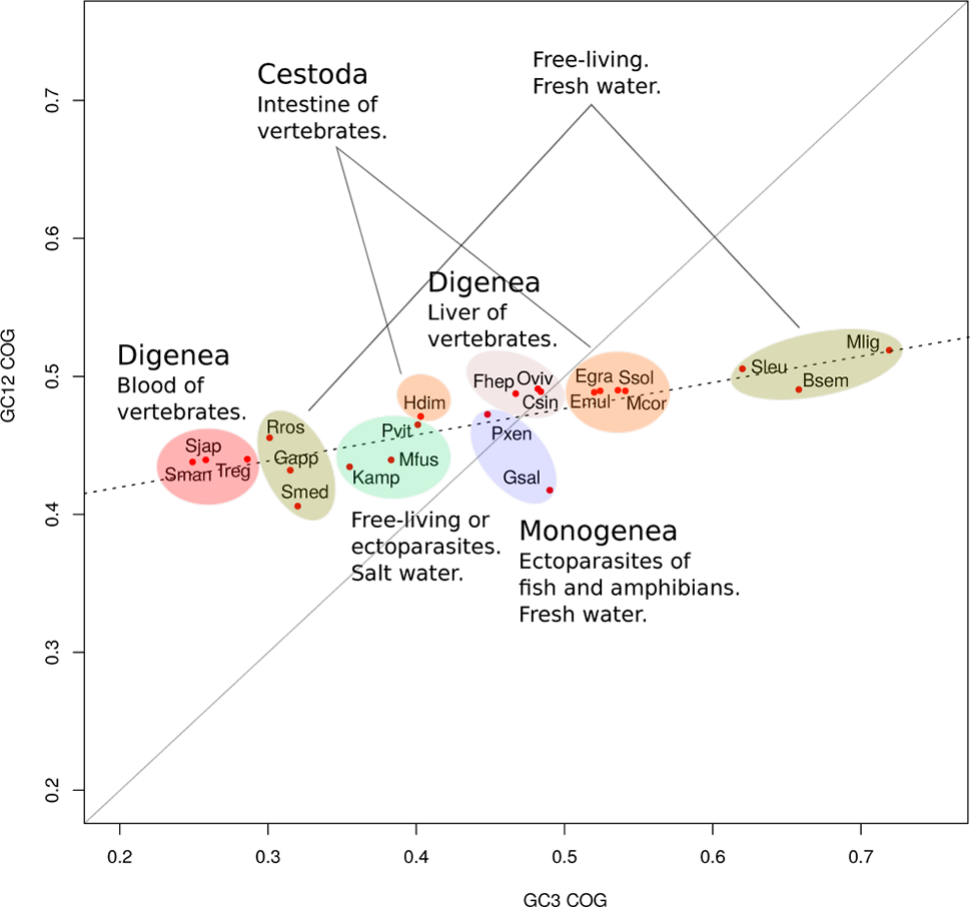
GC variation and lifestyles correlations. Mean of GC12 versus GC3 for orthologue groups. GC3 is a better discriminator of species than GC12. GC content was linked to the ecological niche of the adult form.

The relation of GC values in 1^st^ and 2^nd^ position versus those presented in 3^rd^ codon position (neutrality plot) is usually used to evaluate if the variations in codon usage are driven by mutation or selection. Neutrality plots for the 22 organisms based on the 700 orthologue genes were analyzed. In all cases, low slopes were found for the regression curve (maximum value of 0.2) (**Supplementary Figure 1**). Careful inspection of the plots indicates that this can be explained by a low variability of GC1/2 among the genes (ranges between 0.4 and 0.6). These results suggest a contribution of selection in shaping codon usage for these organisms. To visualize all species together, we plotted the GC1–2 versus GC3 of the concatenated orthologue groups (COGs) for each species (see Methods). Expectedly, while GC1–2 presented little variation across species, the best discriminator was variation at GC3 (**Figure 2**). For example, between the most GC biased genomes, the GC-poor *S. mediterranea* and the GC-rich *M. lignano*, there was only 10% variation in GC1–2 axis but 40% variation in GC3. Interestingly, based on GC3 variability, we found two distinctive clusters within the freshwater “turbellarians,” trematodes and cestodes. The trematodes species clearly differentiated the blood-dwelling flukes grouped on the lowest side of the GC3 spectrum to the food-borne liver flukes allocated in the middle upper GC3 range. Similarly, cestodes tend to cluster on the upper side of the GC3 range with the exception of the Hymenolepidae that fall on the lower-middle of the GC range. Freshwater “turbellarians” showed the largest variability in both the GC12 and GC3 range grouping into very distant clusters. However, we found no clear evolutionary-GC content correlation as species belonging to different lineages were mixed in both groups.

### GC Bias in Coding Sequences Affect Codon Usage

**Figure 3:**
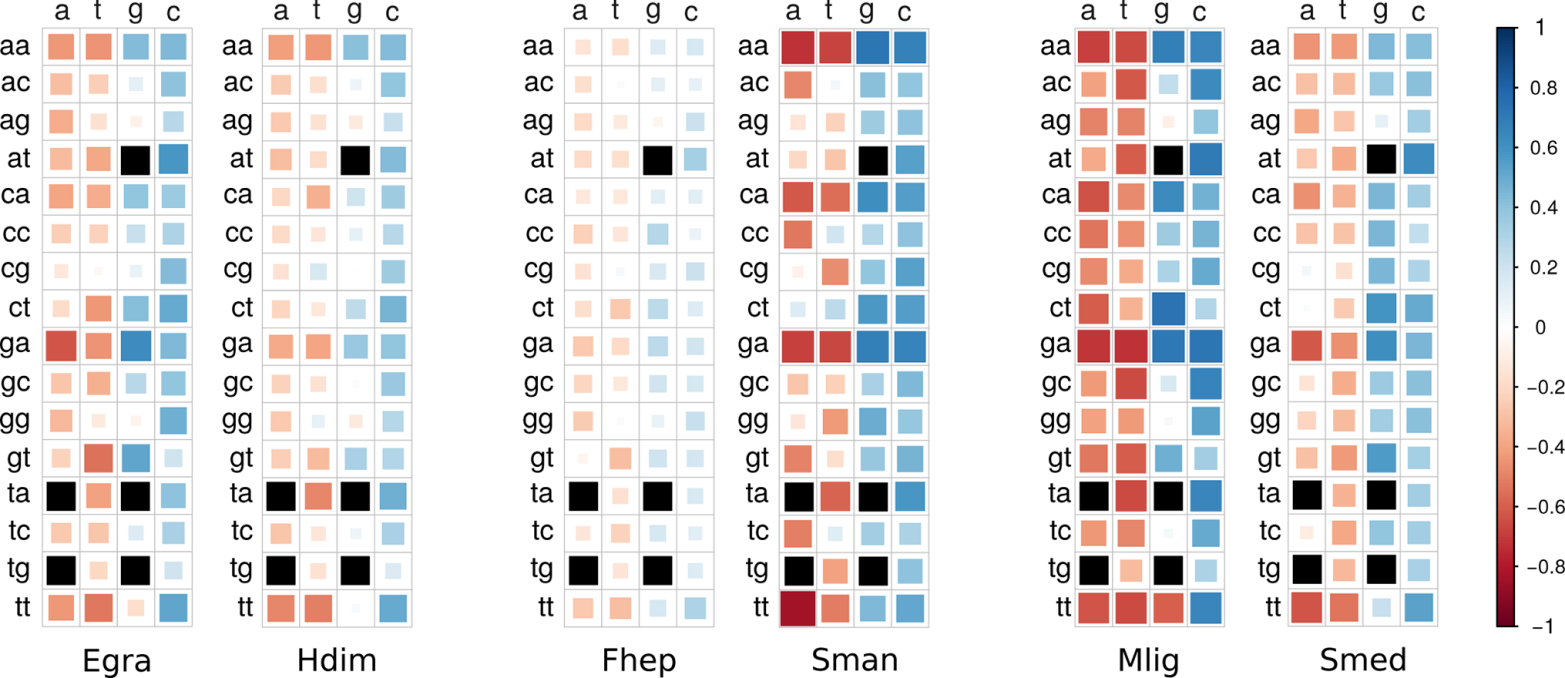
Heatmaps of correlations between codon usage and GC3. Correlation values are coded according to the color scale depicted in the side bar (blue = positive, red = negative). Color intensity and the size of the rectangle are proportional to the correlation coefficients. Black squares: ATG (Met), TGG (Trp), and STOP codons. Organism name abbreviations are as in **Table 1**.

**Figure 4:**
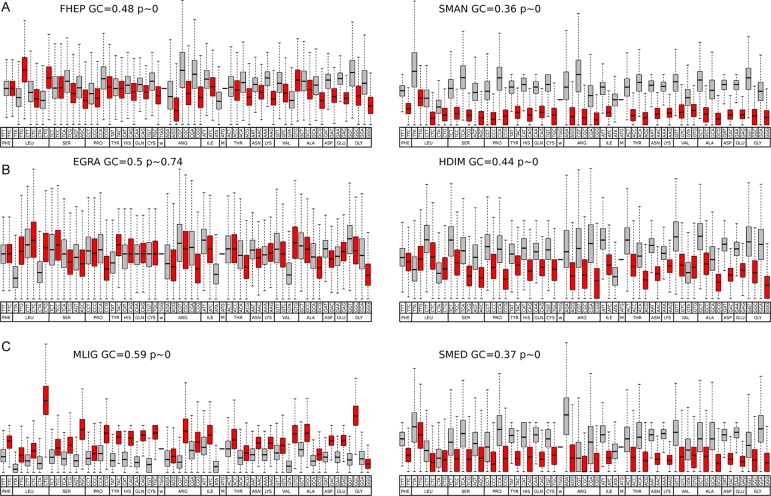
Codon usage in representative species of flatworms. Boxplot of codon usage in six representative species of the diverse lineages and GC content across flatworms. **(A)** comparison of the trematodes *F. hepatica* and *S. mansoni*; **(B)** comparison of the cestodes *E. granulosus* and *H. diminuta*; **(C)** comparison of the free-living *M. lignano* and *S. mediterranea*. GC- and AT-ended codons are grey and red coded for ease of visualization. Mean GC values and p values are indicated.

Codon usage bias is a general feature of genomes that has been widely associated with GC content (mutational bias) and natural selection (Bernardi and Bernardi, 1985; Sharp et al., 2010; Plotkin and Kudla, 2011). In this context, we decided to study its extent and its relationship with the genomic GC frequency discussed in the previous section. To this end, heatmaps were plotted to visualize the correlations between GC3 and codon usage (Palidwor et al., 2010). While codon usage bias is observed for all species, the more compositionally skewed organisms (the three plots on the right) show more intense correlations, indicating that the phenomena are stronger in these organisms as might be expected. Also, in most cases, the correlation values were positive for GC-ended codons and negative for the AT-ended ones (**Figure 3**). To further characterize this relationship, the distribution of the frequency of synonymous codons was analyzed for all organisms. A dramatic split of GC- vs AT-ended codons is observed in the species with the more biased GC genomes as the AT-rich model trematode *S. mansoni*. Notably, the split is seen in opposite directions in the free-living flatworms *S. mediterranea* and *M. lignano* that are at the extremes of the GC distribution (**Figure 4**). A less marked but significant difference is seen within the cestodes consistent with a more balanced GC content, a feature confirmed in the analysis of the 22 species across flatworm diversity (**Supplementary Figure 2**). These observations suggest that genome-wide mutational bias is a major contributor to the observed codon frequency profiles for each organism.

The GC3 vs ENC charts for the analyzed species (**Supplementary Figure 3**) show a combined contribution of selection and mutation for most of the species supporting the trends observed previously, while heavily biased genomes of Schistosomes fall on the curve, suggesting a strong effect of mutational bias.

### Differential Codon Usage Is Associated With Expression Levels

**Figure 5:**
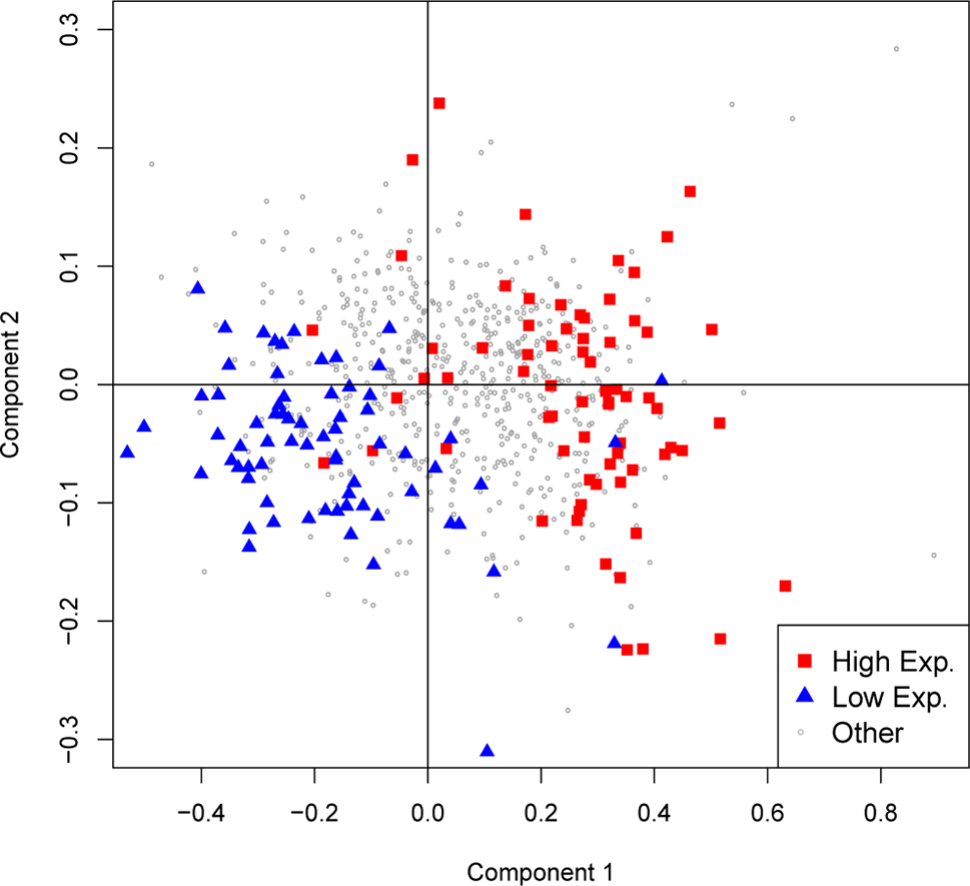
GC variation and expression level in *S. mansoni*. The first two axes of a principal component analysis on codon frequencies are plotted. Genes with expression levels above the 90^th^ percentile or below the 10^th^ percentile are colored red and blue, respectively.

While mutation bias influences codon usage in a genome-wide fashion, selection may also act on coding sequences to select for specific codons. This theory predicts that more frequent codons are actually more efficient and/or more accurate during translation of the mRNA (Sharp et al., 2010; Plotkin and Kudla, 2011). To test if this phenomenon is observed in flatworms, steady-state mRNA levels for the available species were collected to differentiate high- and low-expression genes. As shown in **Figure 5**, where the two main components of a PCA of codon usage for *S. mansoni* are plotted, high- and low-expression genes do present a distinct usage profile. Interestingly, when comparing the 10% higher- and lower-expressed genes in the adult stage, a preference for using GC-rich codons is observed narrowing the distribution in the highly expressed genes and extending it in the lowly expressed (**Supplementary Figure 4**). Even though these results may be explained by biased repair mechanisms acting on highly transcribed sequences, this result is also compatible with translational selection acting on these genes to drive the observed bias.

### Amino Acid Usage Is Also Biased in Diverse Flatworm Lineages

**Figure 6:**
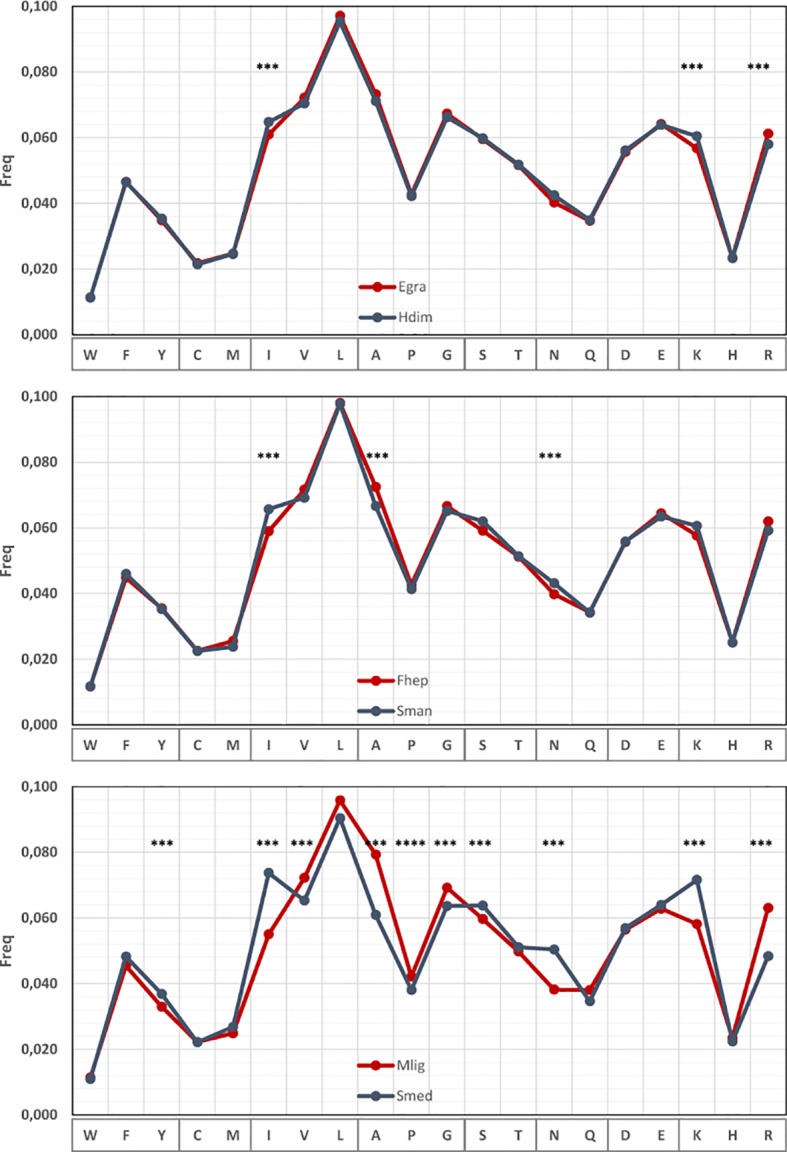
Amino acid usage differences within flatworms. Amino acid usage in a set or 700 orthologous genes compared in pairs of species with different global GC. The amino acids showing significant differences are indicated.

The strong bias observed in codon usage is expected to be associated with the 3^rd^ codon position allowing synonymous changes. However, variations might also exist at the amino acid level (Li et al., 2015). To investigate this, we analyzed the amino acid usage within the set of 700 orthologue genes in pairs of species. Subtle but significant differences in the amino acid frequencies can be detected in cestodes and trematodes mainly involving the amino acids encoded by AT-rich [Ile (AUR), Asn (AAY), Lys (AAR)] or GC-rich [Arg (CGN), Ala (GCN)] codons (**Figure 6** and **Supplementary Table 1**). The variations are more pronounced in the comparison of the free-living species, and in all the cases, the variation follows the GC trend of the species. Since these results are based on a set of orthologue genes, the variations in amino acid frequencies indicate that not only synonymous changes account for the variability observed but also non-synonymous substitutions are taking place.

### Synonymous and Non-Synonymous Substitutions in Conserved Orthologous Genes

**Figure 7:**
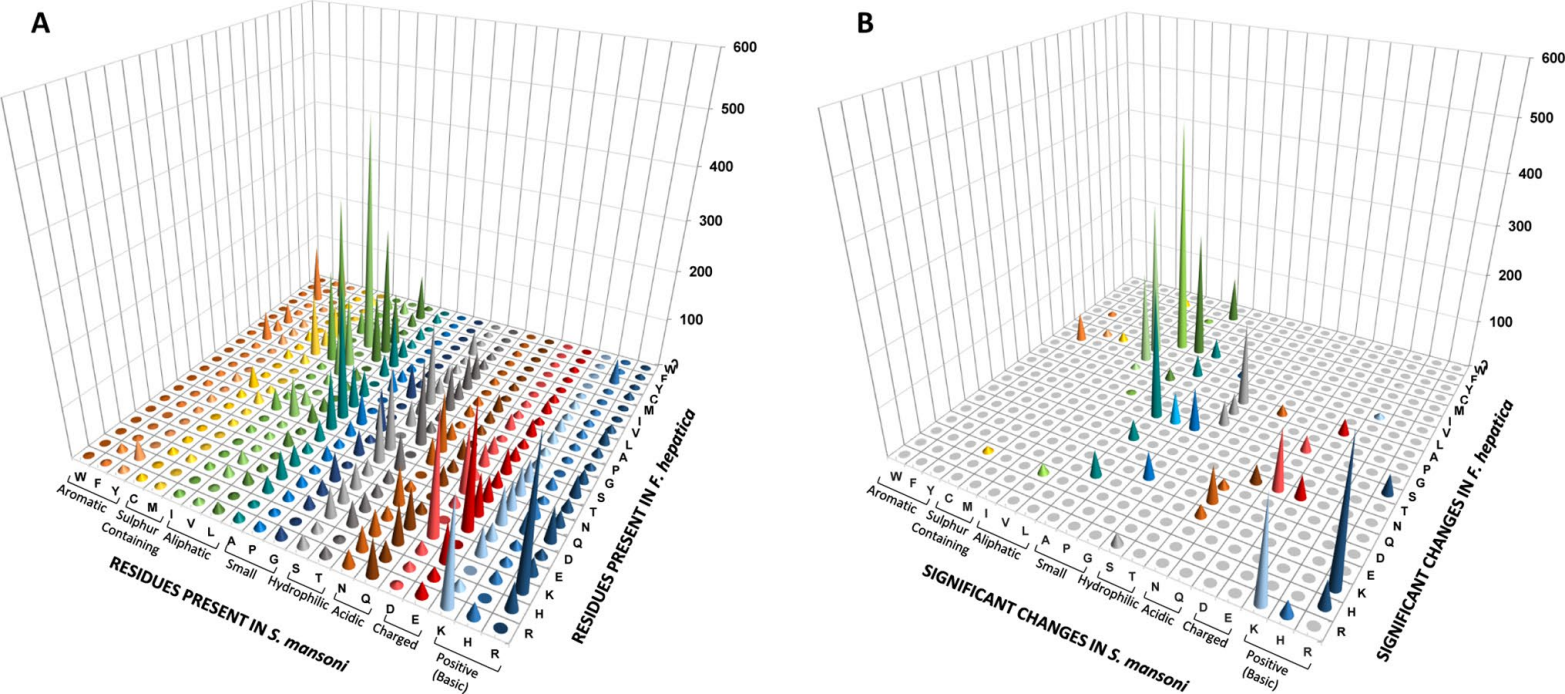
Non-synonymous amino acid changes between *F. hepatica* and *S. mansoni*. **(A)** Graphical representation of a substitution matrix of amino acids present at orthologous positions in alignments. The residues are indicated. Amino acids present on *S. mansoni* are ordered and color coded by property. **(B)** Non-synonymous reciprocal changes that show significant differences.

We decided to investigate if particular directional changes could be detected when analyzing orthologous positions in the three paired species. For this, we selected the ungapped regions of each pairwise alignment of orthologues and generated substitution matrixes based on the aligned orthologous positions. Amino acid conservation was generally high, with tryptophan (Trp) and glycine (Gly) as the more conserved residues in all the species, confirming Ile, Ser, Ala, Asn, and Arg as the more variable (**Supplementary Table 2**). Expectedly, the most frequent changes involved amino acids with similar properties (**Figure 7A**), particularly those involving aliphatic and hydrophilic residues. However, several reciprocal changes showed significant differences in the counts (**Figure 7B**). Similar effects can be observed in the other pairwise comparisons (**Supplementary Figure 5**).

To gain further insights into these phenomena, we evaluated the substitutions at the codon level generating substitutions matrices for the three pair of species selected (**Supplementary Table 3**).

**Figure 8:**
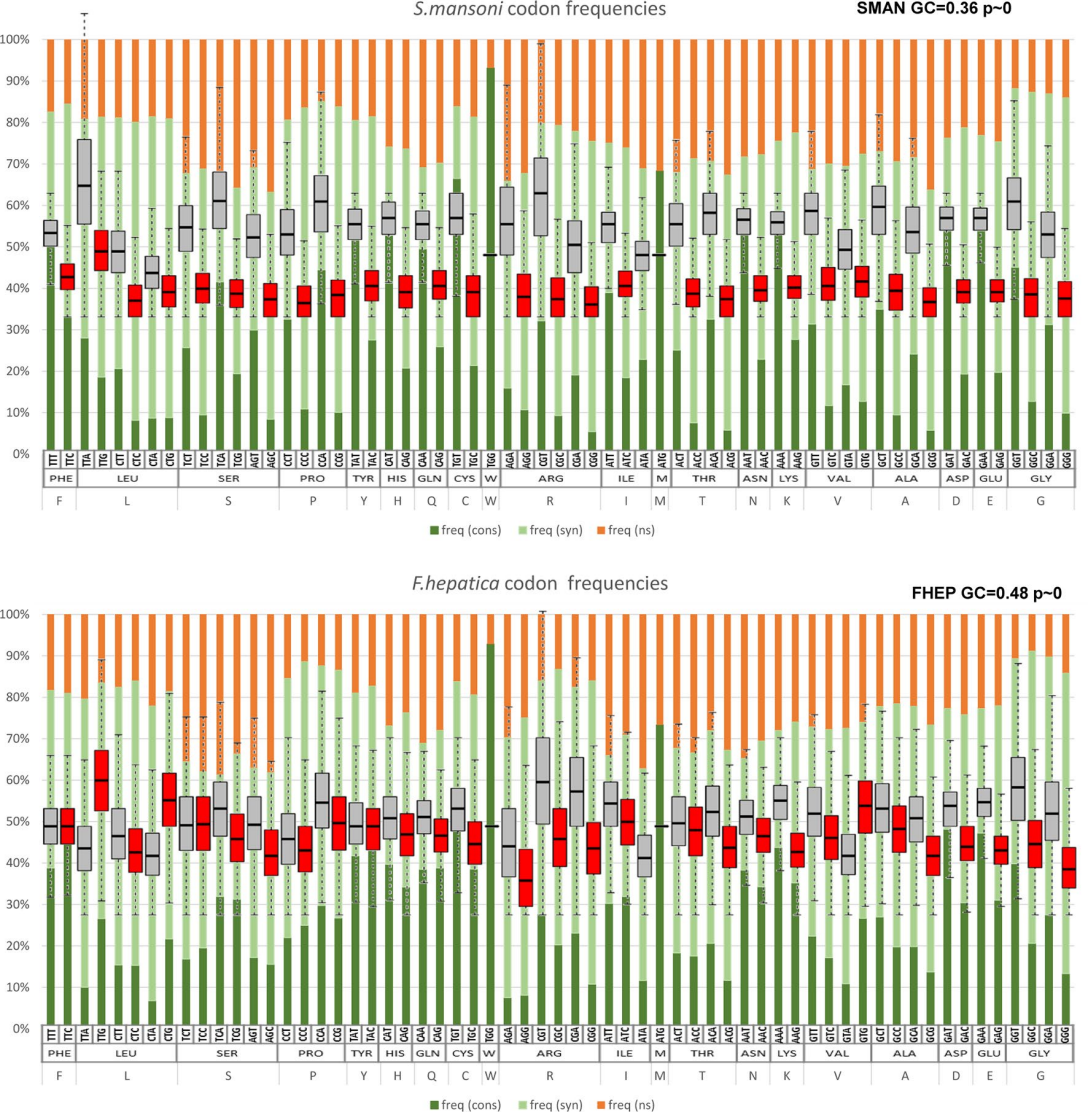
Codon changes between *S. mansoni* and *F. hepatica*. The substitution state for each codon in pairwise alignments is represented in bars, indicating conserved codons (dark green), synonymous substitutions (pale green), and non-synonymous changes (orange). Note the enrichment of synonymous changes in GC3-poor codons in *S. mansoni* (top) in comparison with *F. hepatica* (bottom). For comparison, the figure is overlaid with the codon frequencies of **Figure 3**.

The conservation at codon level as expected was much lower with a strong component of synonymous changes (**Supplementary Table 3**). The lower frequency of GC-rich codons in *S. mansoni* (depicted in **Figure 4**) is explained by a marked increase of synonymous substitutions toward AT-rich codons (**Figure 8**). Similarly, in *M. lignano*, synonymous substitutions toward GC-rich codons are associated with reduced AT codon counts (**Supplementary Figure 6**).

**Figure 9:**
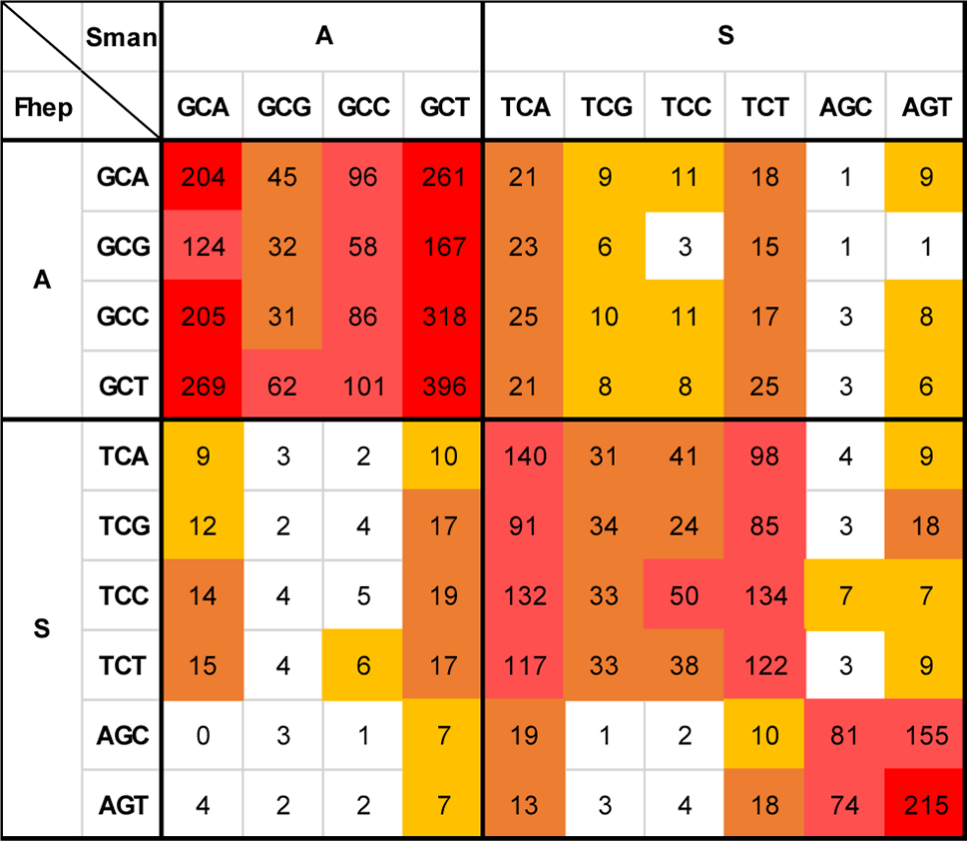
Non-synonymous changes between *S. mansoni* and *F. hepatica*. Detail of the substitution matrix of **Supplementary Table 3** of the changes involving Ala and Ser (*S. mansoni* codons in columns, *F. hepatica* in rows). Note the lower-than-expected counts of changes toward GC3-rich codons and the enrichment in synonymous substitutions that imply two substitutions.

The analysis of the non-synonymous changes at the codon level showed an increased complexity (**Supplementary Table 3**). One striking feature is that amino acid changes involving two substitutions are more common than those explained by simple substitutions. A detailed example is presented in **Figure 9**.

Ala is a relatively GC-rich codon (GCN) that is frequently substituted by the more GC neutral Ser (TCN + AGY) and vice versa (**Figures 7** and **9**). When this substitution takes place, it is expected that the GCN codon would change for the corresponding TCN variant, i.e., that GCA would turn into TTA and GCG into TCG. In 49 positions in the alignment, a GCG coding Ala is present in *F. hepatica*, while Ser codons are present in *S. mansoni* (second row). The simple transversion GCG to TCG is underrepresented with only six occurrences, while the changes toward TCA and TCT are more abundant (23 and 15 occurrences, respectively). Similarly, the GCC to TCC transversion (third row) represents only a 15% of the Ala (GCC) changes to Ser, while the more AT-rich variants (TCA and TCT) represent more than 56% of the substitutions. Notably, when the AT-ending Ala codons (GCA and GCT, 1^st^ and 4^th^ rows) are substituted, the more common codon is the one expected by a single 3^rd^ position change.

The reciprocal Ser (in *F. hepatica*) to Ala (in *S. mansoni*) changes are less common (169 times vs 263 Ala to Ser), but again, in 77% of the cases, they are enforced to 3^rd^ position T or A irrespective of the original Ser codon. Similar effects can be seen when analyzing other amino acid changes (**Supplementary Table 3**) and particularly in those regarded as significant (from **Figure 7**) as Ile to Val or Lys to Arg (**Supplementary Figure 7**).

Taken together, these results are strongly suggestive of a combined effect of mutation and selection in order to maintain both the compositional GC skew of the species and the property of the coded amino acid. In other words, whenever an amino acid change occurs through a simple substitution, this is then rapidly switched to those that follow the GC of the species.

## Discussion

Platyhelminthes classes show a wide range of GC composition, even within groups. Our results show that GC3 content explains most of the observed variability in the codon usage as reflected by the variation in the RSCU values. Based on GC3 variability, we found different clusters within the free-living species, trematodes, and cestodes. This can be clearly seen when the species tree is compared with the tree representing codon usage similarity. Indeed, Platyhelminthes show great differences between both trees, while this phenomenon is not seen in other models as different as bacteria and hexapoda (Behura and Severson, 2012; Dilucca et al., 2018). A similar study in nematodes show a comparably wide distribution of GC values, although the variations are more consistent with the phylogeny (Cutter et al., 2006). These results suggest more recent and strong compositional shifts for these groups of organisms. Further work is needed to explain this particular phenomenon in flatworms.

Codon usage bias is a generalized feature of the genomes of many organisms that is deeply influenced by evolutionary phenomena and results basically from the balance between mutational bias and natural selection (see Plotkin and Kudla, 2011, for a review). To assert the relative influence of these two factors, a plot of GC1–2 vs GC3 for all the species taken together was generated. This “pseudo” neutrality plot shows a slight slope, indicating that GC3 shows a different behavior when compared with GC1–2, a result that is generally taken as evidence that selection is acting to shape codon usage (Sueoka, 1988). Furthermore, this plot shows clearly distinctive clusters within trematodes, cestodes, and free-living species based on GC content. Notably, these differences seem to blurrily reflect the diversity of lifestyles and niches of the diverse flatworms. The observed differences question the use of single species as a model for each class; a clear demonstration of this is the differences between the model *S. mansoni* and other trematodes observed in the boxplot of **Figure 4**. Codon usage bias in flatworm mitochondrial genomes has also been reported (Le et al., 2004; Mazumder et al., 2018). Even though considering genic and genomic large differences, they may follow evolutionary pressures independent from the nuclear genomes.

Interestingly, a similar study across nematodes found robust evidence for selection on codon usage bias in free-living species, a feature found marginally in parasitic ones, and particularly in the most compositionally biased (Cutter et al., 2006). The association of selective bias in free-living or parasitic species is not clear-cut in the case of flatworms, which might be reflecting diverse evolutionary strategies.

In agreement with the hypothesis of translational selection driving synonymous codon usage bias, we observe a clear association of gene expression levels with codon usage where highly expressed genes are rich in GC-rich codons, while the opposite is observed for low-expression genes. Similar results have been previously reported for cestodes, among others (Chen et al., 2013; Yang et al., 2014; Yang et al., 2015; Huang et al., 2017; Maldonado et al., 2018). Even in highly AT-biased genomes—as observed for the schistosomes—the GC content of highly expressed genes is relatively high when compared with that of the general trend. It is worth to mention that the bias in repair mechanisms of actively transcribed DNA has also being proposed to explain this observation.

The observed differences in CG and codon usage among these organisms are also reflected in the amino acid composition. Recently, Li et al. (2015) show the strong relationship of synonymous codon usage and differential amino acid usage, using a strategy based on classifying amino acids in three groups (high, medium, and low GC content) according to the GC composition of their corresponding codons. Our results on amino acid frequencies in the different flatworm species are consistent with these observations.

Furthermore, when orthologues positions are considered, we mainly observed amino acid substitutions conservative of the physicochemical properties as would be expected. However, these changes frequently involve codons of completely different GC content that follow the differences observed in general GC content of the genomes, i.e., Ile (AUH) vs Leu (CCN, UCR). In this way, AT-rich genomes accumulate changes to amino acids in the low GC group, while the opposite is observed in GC-rich genomes.

Remarkably, when the frequency of a certain amino acid substitution is not reciprocal between two given organisms, the amino acids involved belong to the different groups defined by Li et al. (2015). An interesting case is observed for the Lys to Arg substitution. Even though these amino acids have similar physicochemical properties, they belong to opposite groups, being Lys coded by the most AT-rich group of codons, while Arg is on the highest GC content side.

A detailed analysis of the non-synonymous changes showed a higher-than-expected frequency of codon changes involving two nucleotides. This is paralleled by a marked reduction in the counts of the expected codon substitutions involving a single change. A plausible explanation for this phenomenon is offered by a two-hit mechanism, providing a clear example of the combined effect of mutation and selection. The two-hit hypothesis proposed implies that when a mutation changes the coded amino acid, this non-synonymous substitution is rapidly adapted to the general GC content of the genome by a second synonymous change.

## Conclusions

GC bias has a great influence on synonymous codon and amino acid usage across Platyhelminthes, a feature not shared by all metazoans. Both free-living and parasitic species show the phenomena, and no clear correlation with lifestyles or evolutionary closeness is evident so far. The changes introduced by GC bias impact not only in synonymous codon usage but also in amino acid frequencies. The evidence so far suggests that both mutation and selection are acting to shape the coding strategies of the diverse flatworms.

## Data Availability

All datasets generated for this study are included in the manuscript/supplementary files.

## Author Contributions

GL and SF performed the bioinformatics analysis and contributed in writing the manuscript. GR performed bioinformatics analysis. PS and JT participated in the design of the study and the interpretation of data, drafting the manuscript, and critical revision of its content. All authors read and approved the final manuscript.

## Funding

Comisión Sectorial de Investigación Científica-Universidad de la República (CSIC-UdelaR), Uruguay. Award number: I+D16-516. Specific budget for publication was included in the project.

SF, GL, PS, and JT are researchers of Programa de Desarrollo de las Ciencias Básicas (PEDECIBA) and members of the SNI program of the Agencia Nacional de Investigación e Innovación (ANII) program. GR received a postgraduate scholarship from ANII.

## Conflict of Interest Statement

The authors declare that the research was conducted in the absence of any commercial or financial relationships that could be construed as a potential conflict of interest.
