# Supplementary material for: Compositional Analysis of Flatworm Genomes Shows Strong Codon Usage Biases Across All Classes
Source: Front Genet. 2019 Sep 5;10:771. doi: 10.3389/fgene.2019.00771 (PMC6739440; doi:10.3389/fgene.2019.00771)
Supplement: Supplementary file 4 [file Image_1.pdf]

Supplmentary Figures S1-S7

INDEX

Page

- **SFig1. Neutrality plots with regression line and correlation values of the 22 species...............1**
- **SFig2. Box plots showing codon frequencies of the 22 species..24**
- **SFig3. GC3-ENC plots of the 22 species..47**
- **SFig4. Boxplots representing codon frequencies of 10% high and 10% low expressed genes...70**
- **SFig5. Non synonymous amino acid changes between members of three pairs of organisms of the groups trematoda, cestoda and turbellaria..72**
- **SFig6. Codon changes between *S. mediterranea* and *M. lignano*...74**
- **SFig7. Non synonymous changes between *S. mansoni* and *F. hepatica*....................................76**

SFig1. Neutrality plots with regression line and correlation values of the 22 species (for details see Materials & Methods).

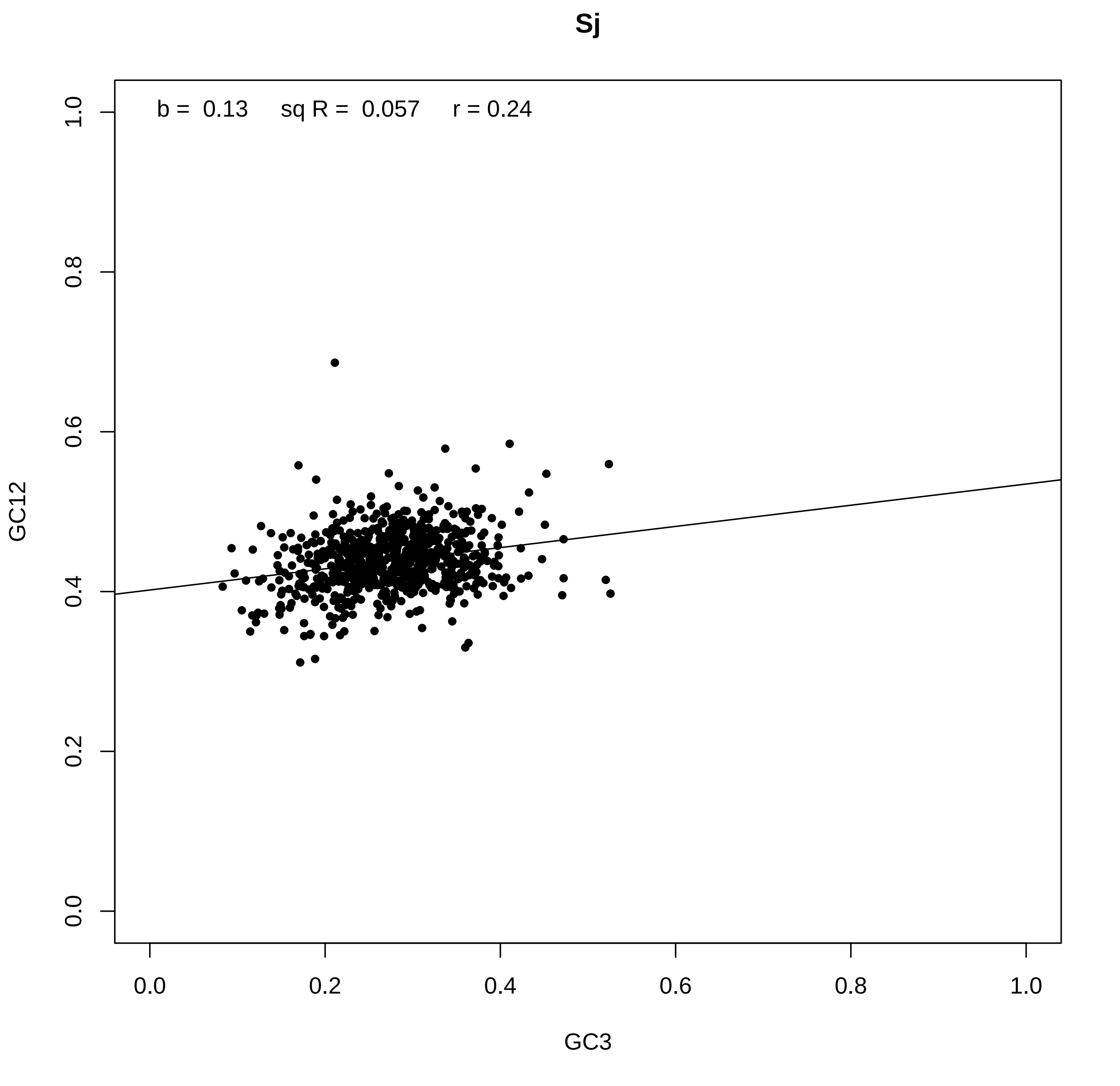

Sj
b = 0.13 sq R = 0.057 r = 0.24
GC12
GC3
0.0
0.2
0.4
0.6
0.8
1.0

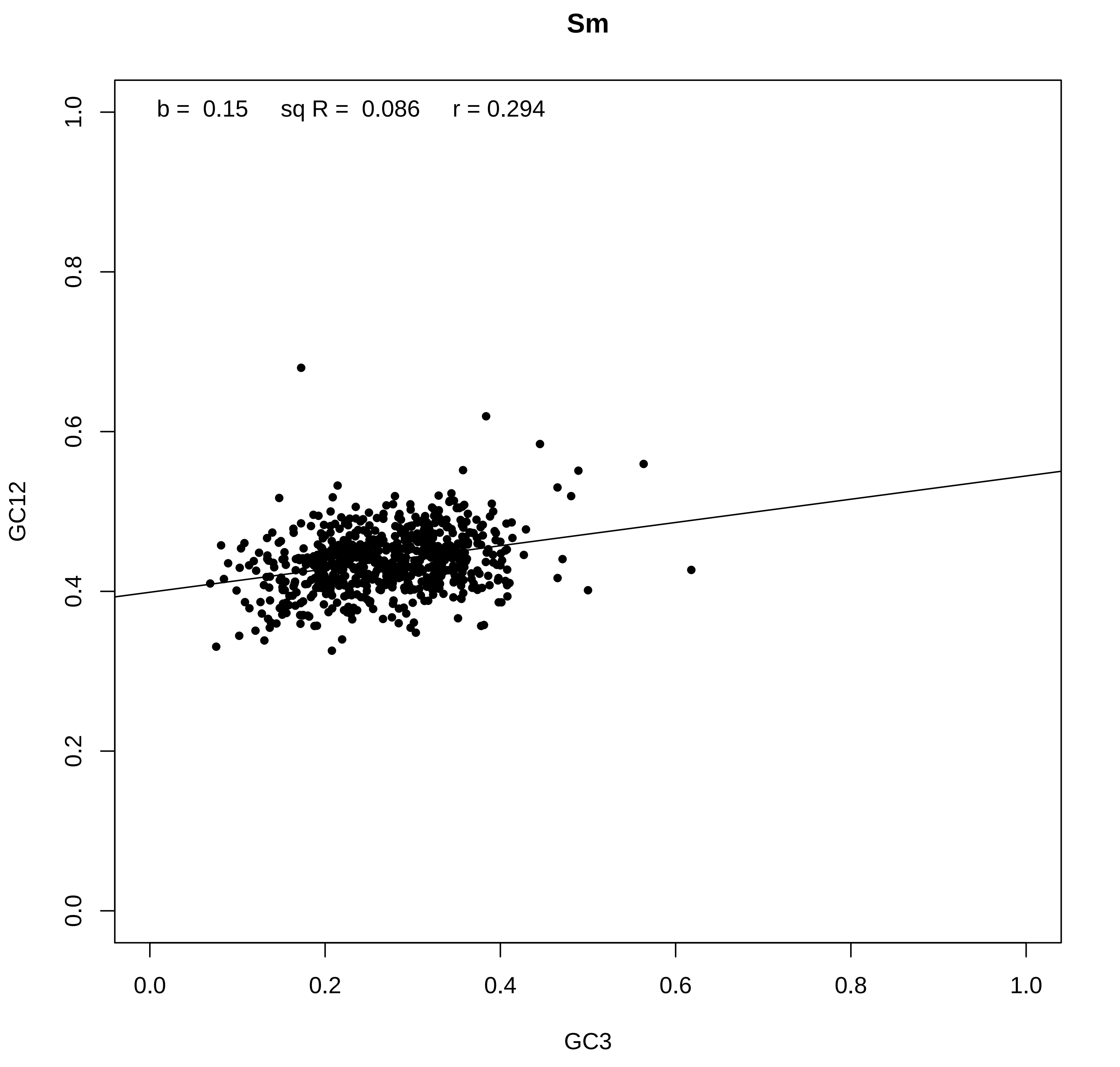

Sm
b = 0.15 sq R = 0.086 r = 0.294
GC12
GC3
0.0
0.2
0.4
0.6
0.8
1.0

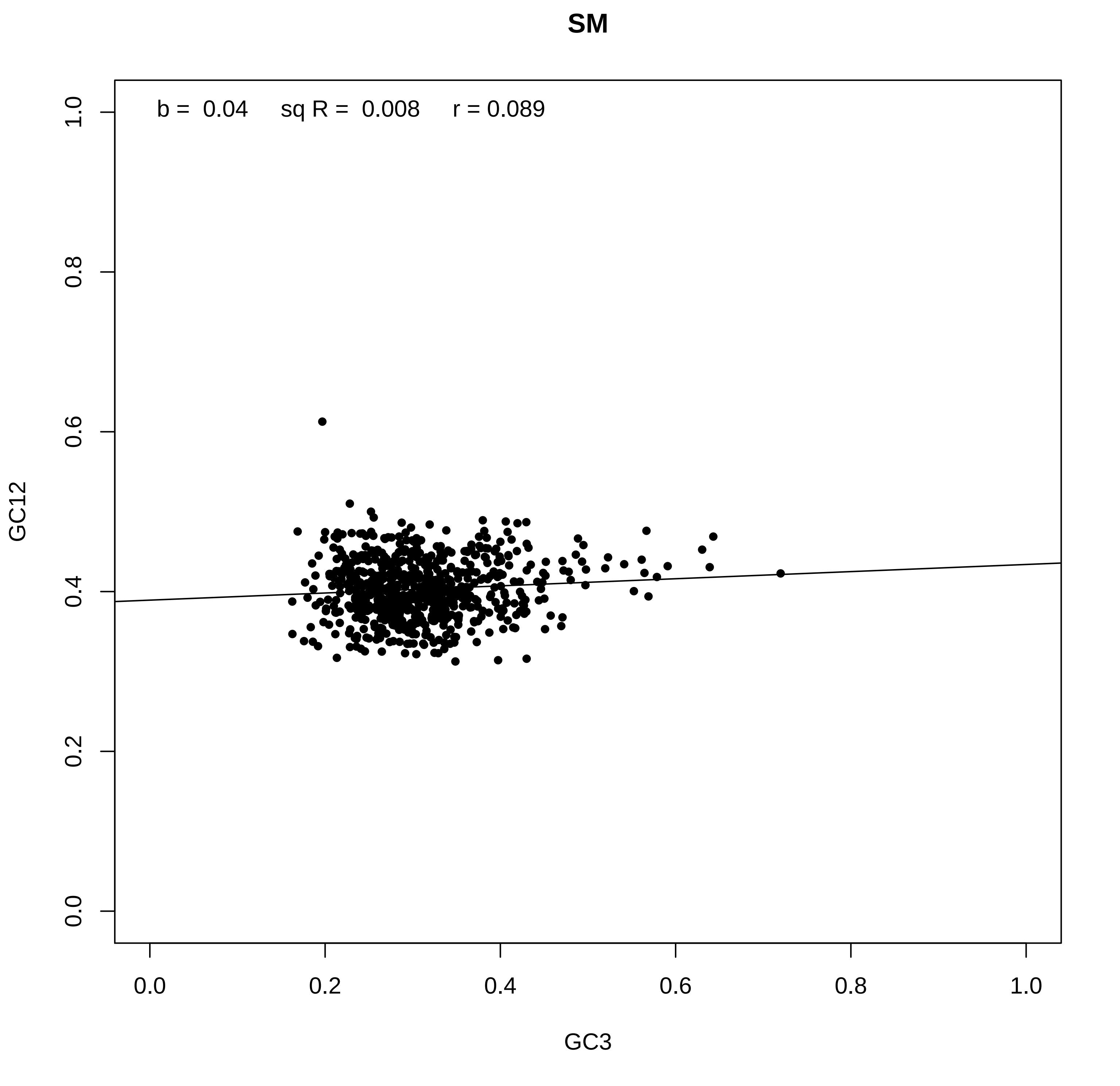
SM
b = 0.04 sq R = 0.008 r = 0.089
GC12
GC3
0.0
0.2
0.4
0.6
0.8
1.0

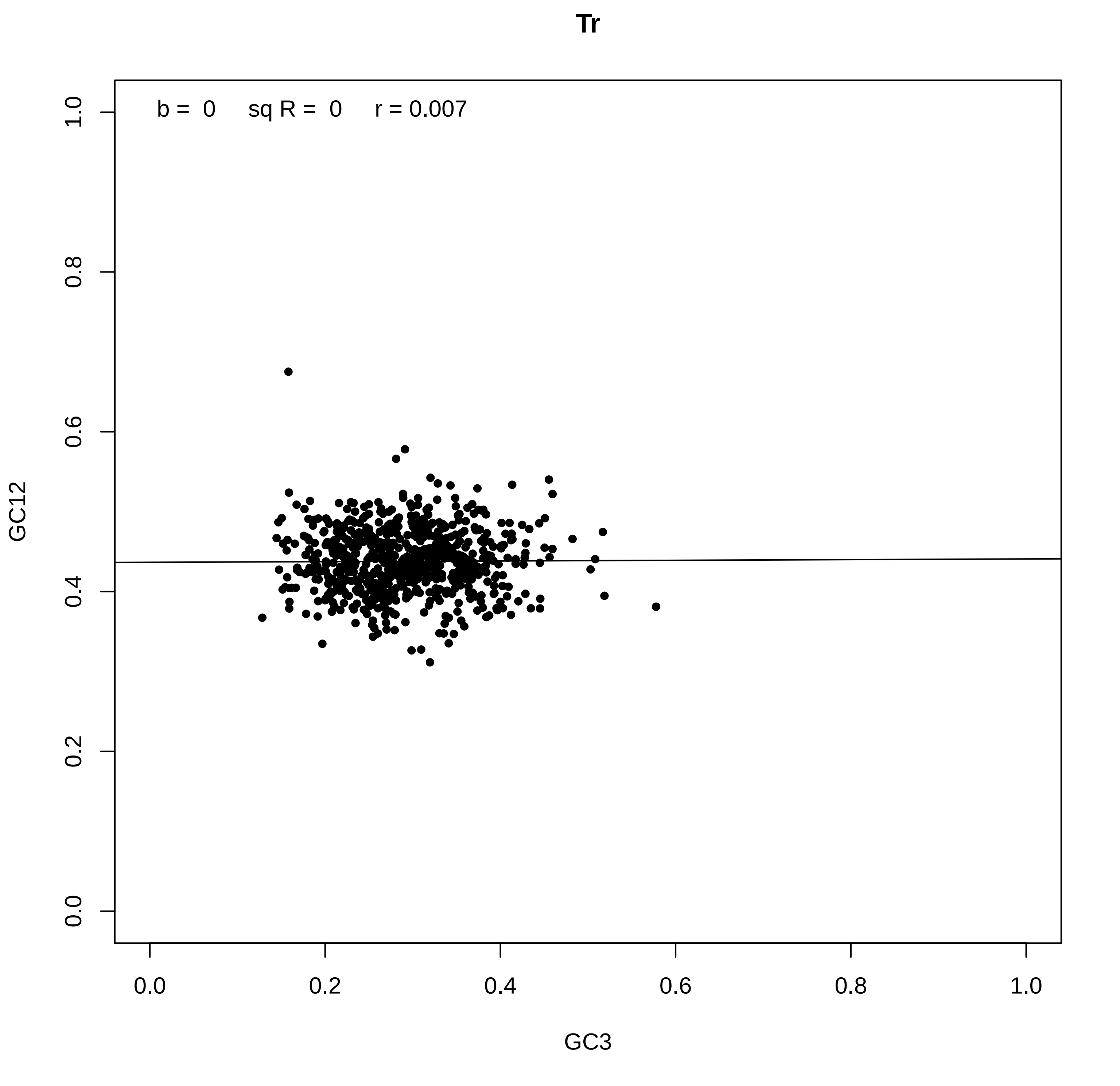
Tr
b = 0
sq R = 0
r = 0.007
GC12
GC3
0.0
0.2
0.4
0.6
0.8
1.0

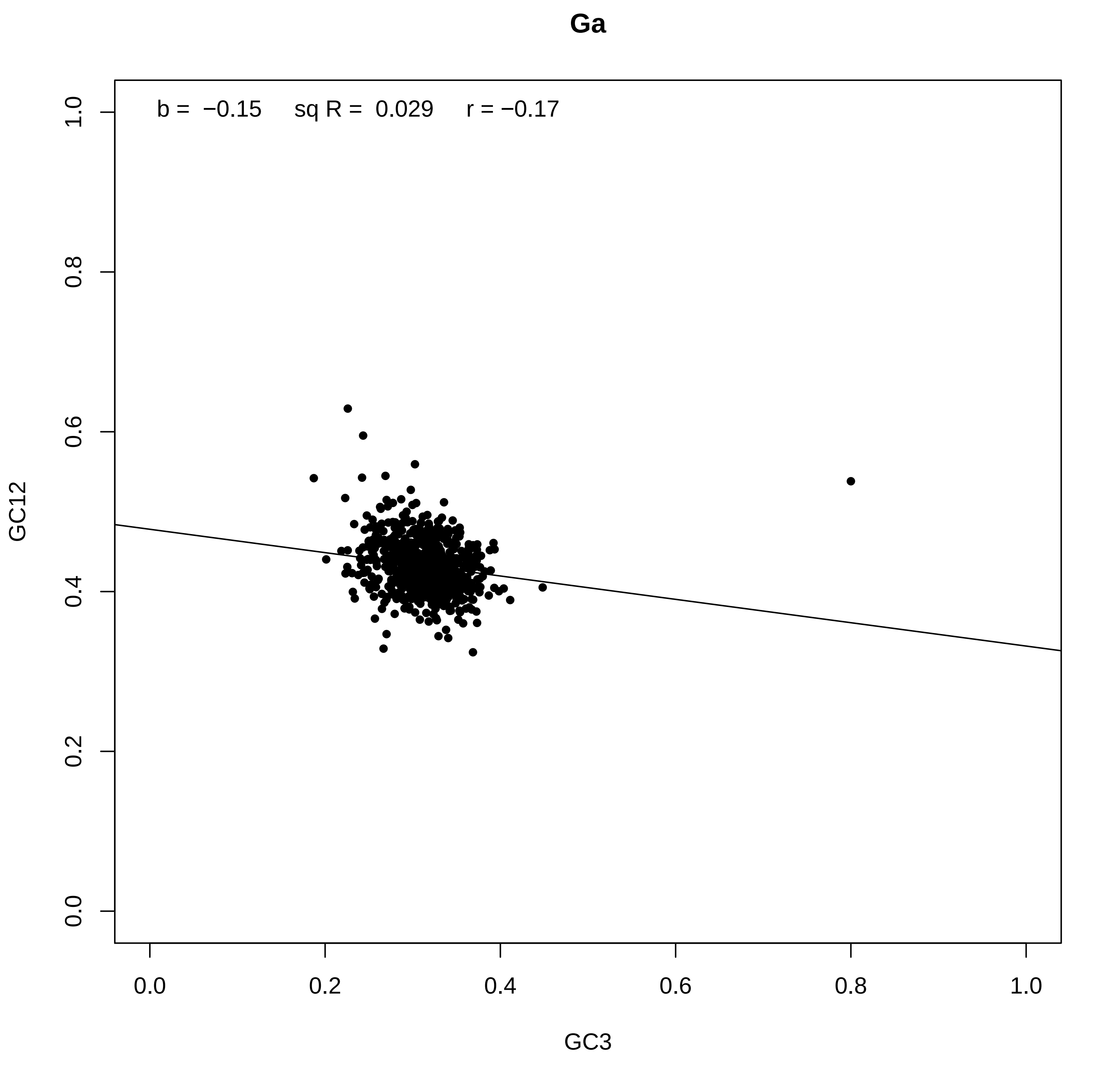

Ga
b = −0.15 sq R = 0.029 r = −0.17
GC12
GC3
0.0
0.2
0.4
0.6
0.8
1.0

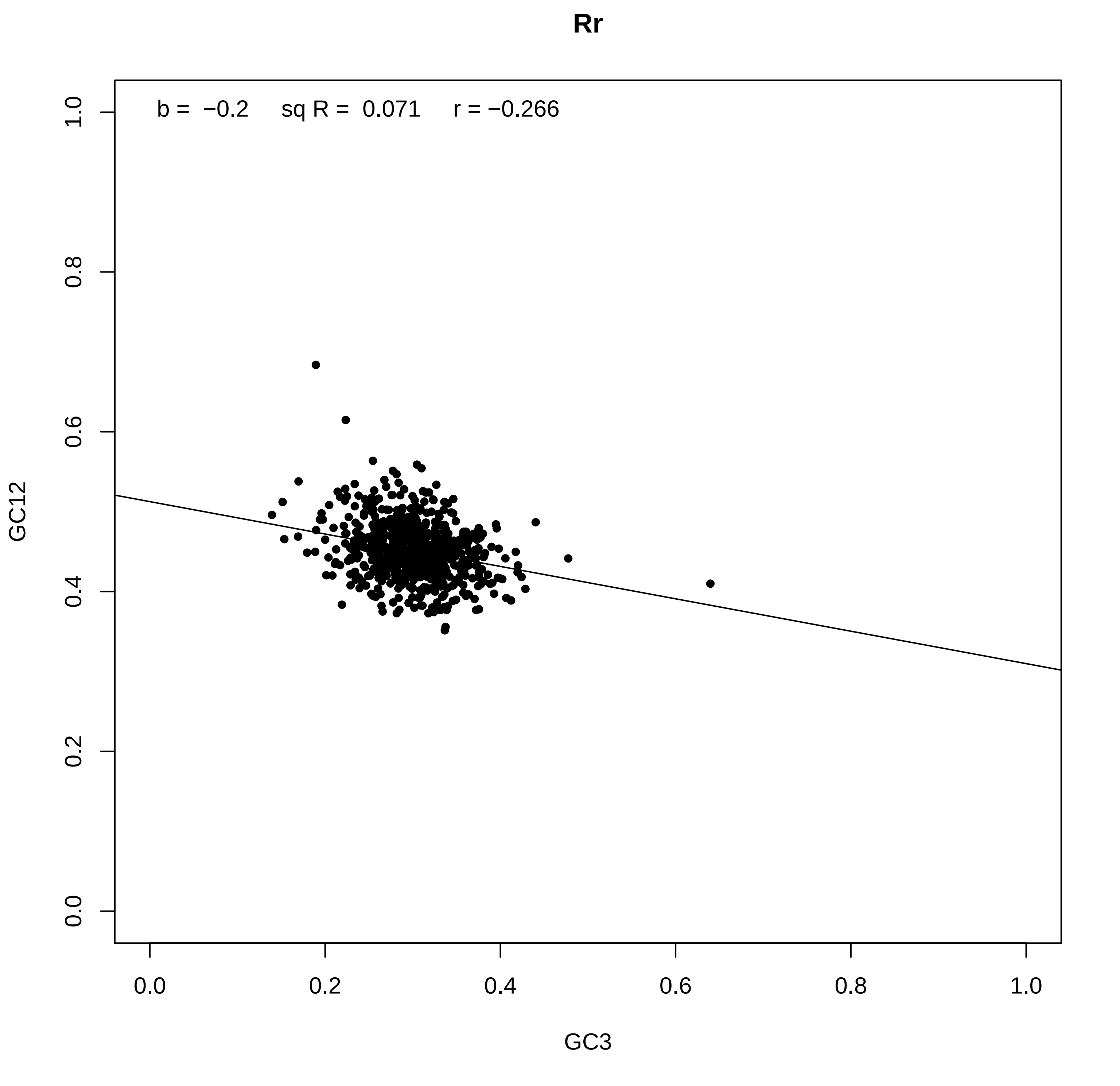
Rr
b = −0.2 sq R = 0.071 r = −0.266
GC12
GC3
0.0
0.2
0.4
0.6
0.8
1.0

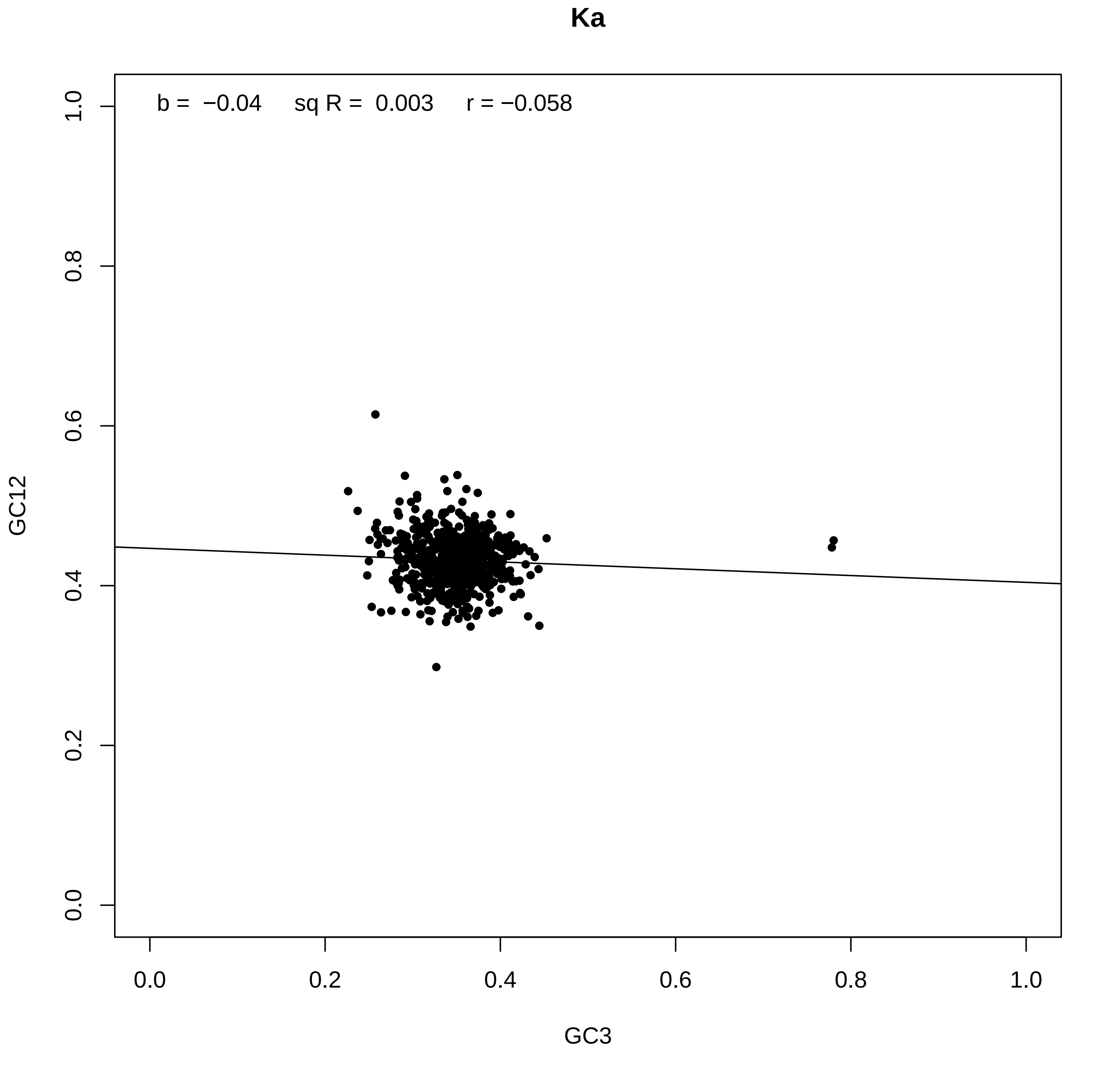
Ka
b = −0.04 sq R = 0.003 r = −0.058
GC12
GC3
0.0
0.2
0.4
0.6
0.8
1.0

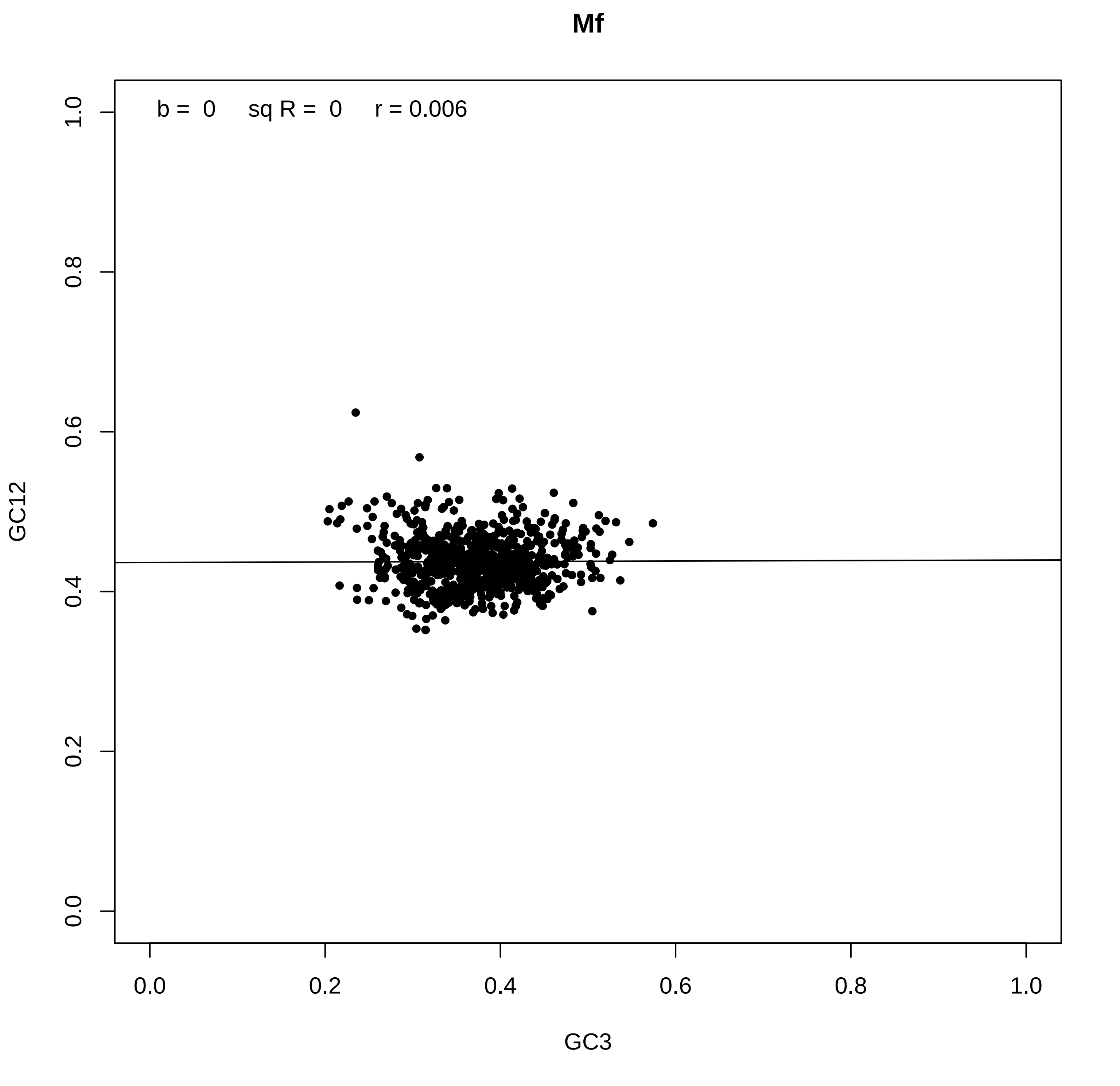
Mf
b = 0 sq R = 0 r = 0.006
GC12
GC3
0.0
0.2
0.4
0.6
0.8
1.0

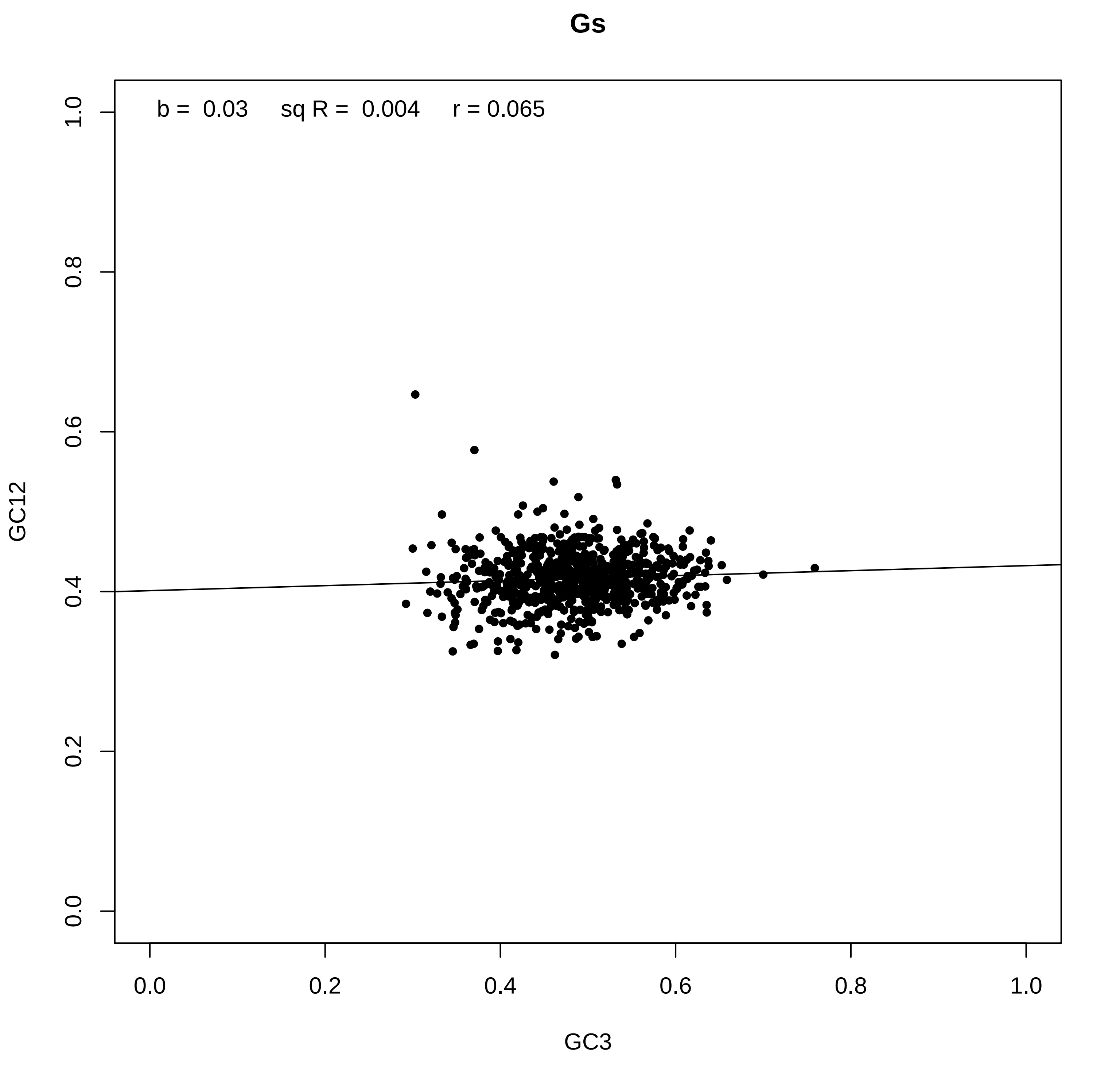
Gs
b = 0.03 sq R = 0.004 r = 0.065
GC12
GC3
0.0
0.2
0.4
0.6
0.8
1.0

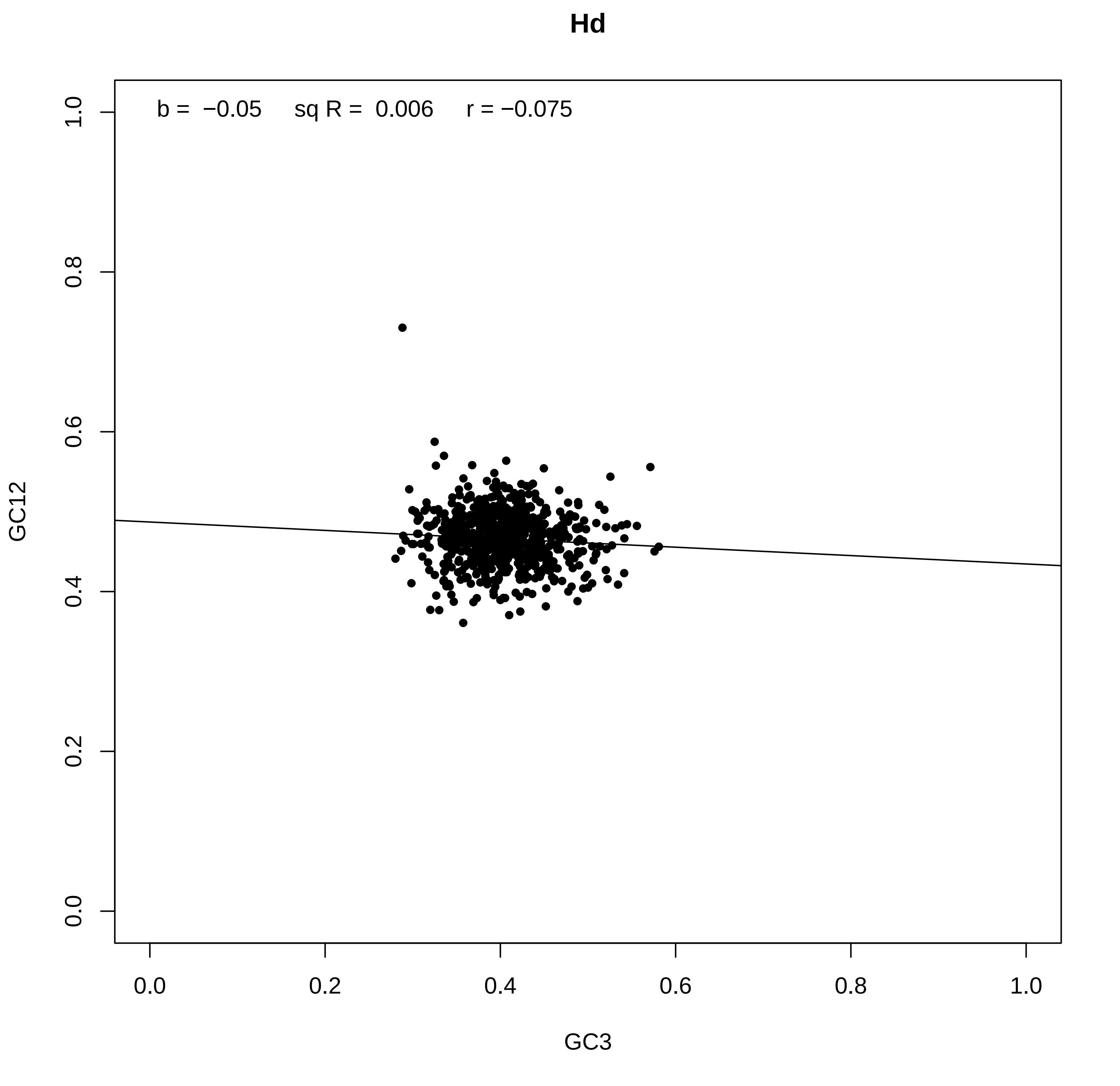
Hd
b = −0.05 sq R = 0.006 r = −0.075
GC12
GC3
0.0
0.2
0.4
0.6
0.8
1.0

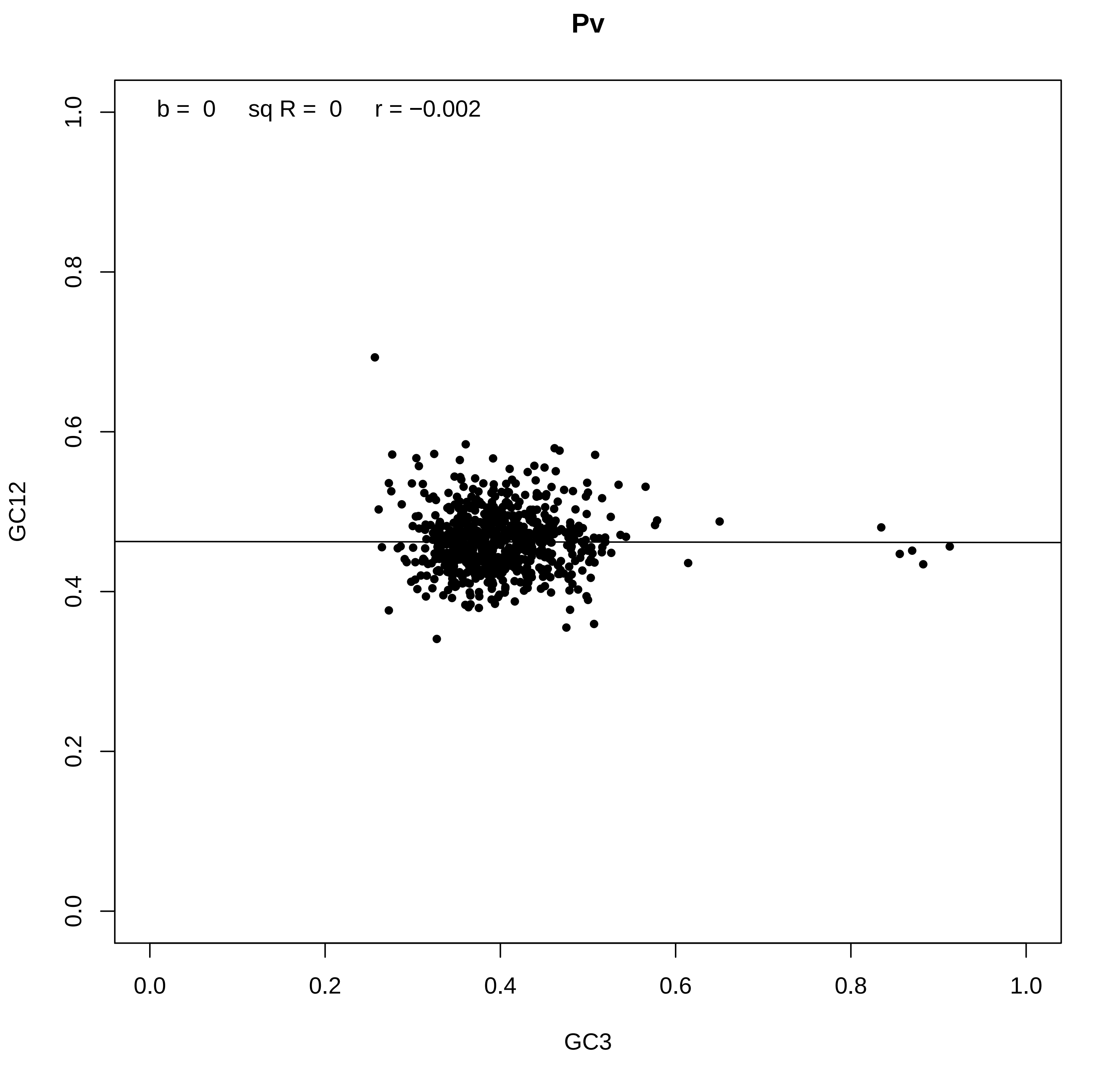
Pv
b = 0
sq R = 0
r = −0.002
GC12
GC3
0.0
0.2
0.4
0.6
0.8
1.0

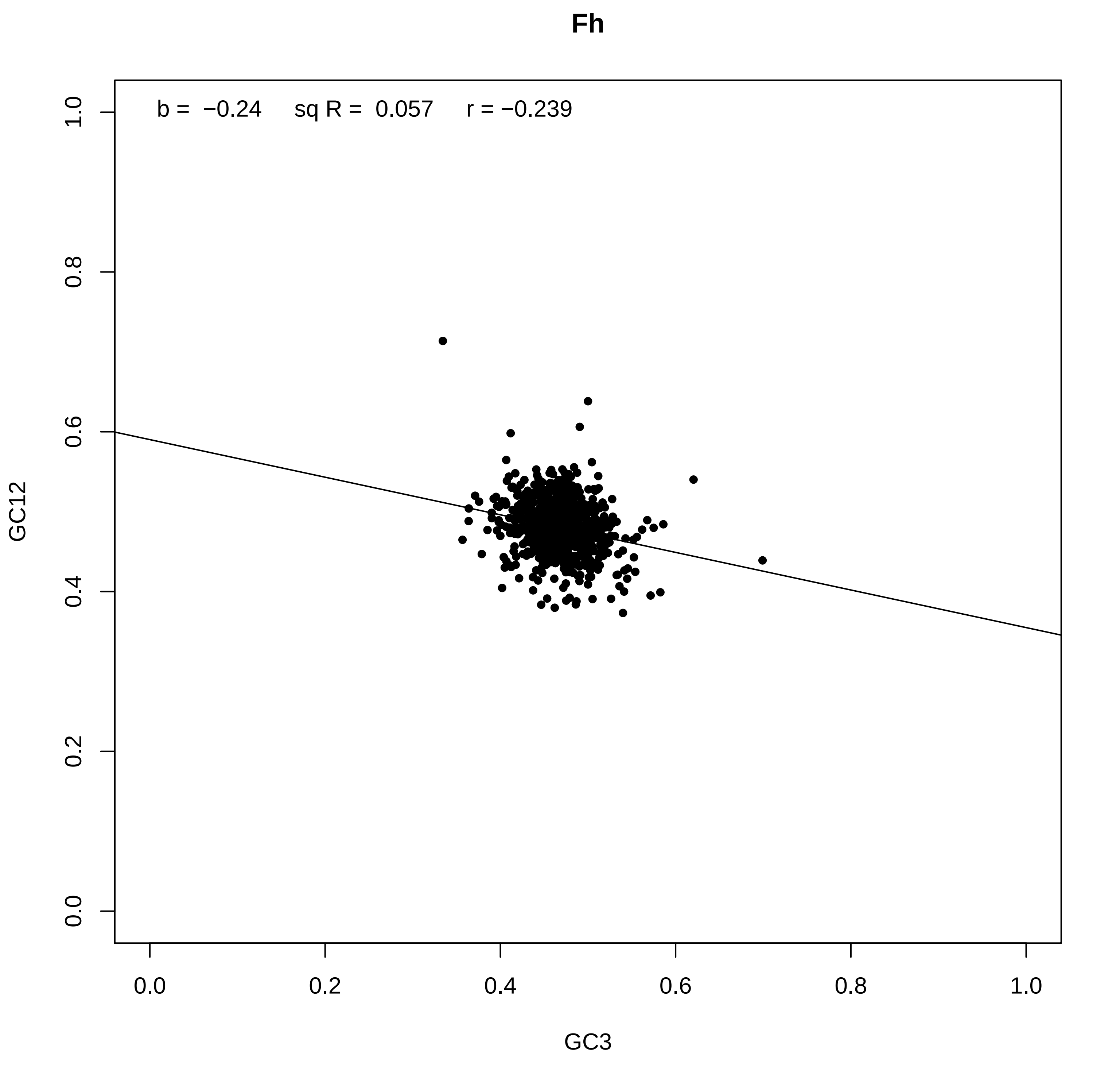
Fh
b = −0.24 sq R = 0.057 r = −0.239
GC12
GC3
0.0
0.2
0.4
0.6
0.8
1.0

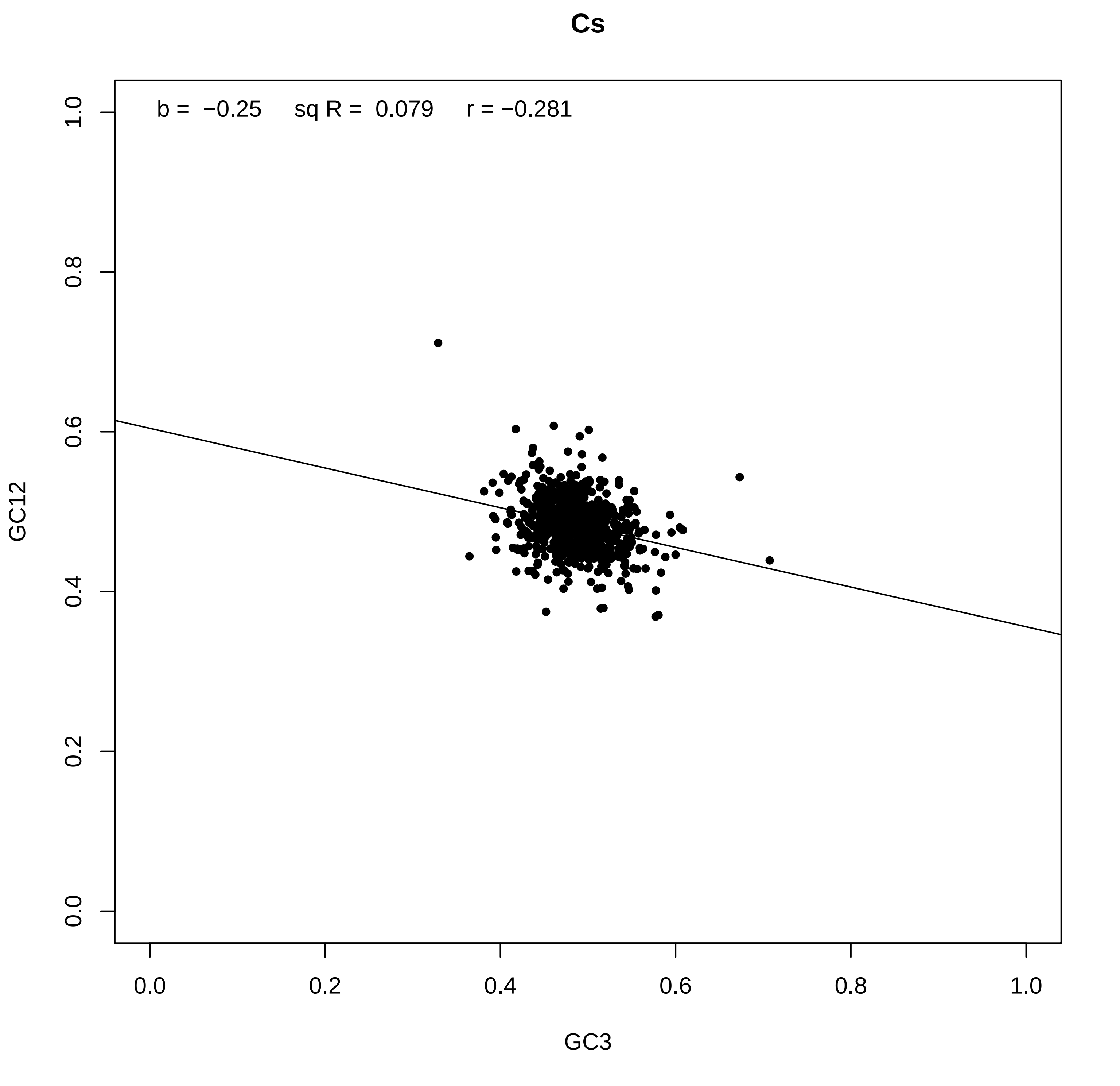
Cs
b = −0.25 sq R = 0.079 r = −0.281
GC12
GC3
0.0
0.2
0.4
0.6
0.8
1.0

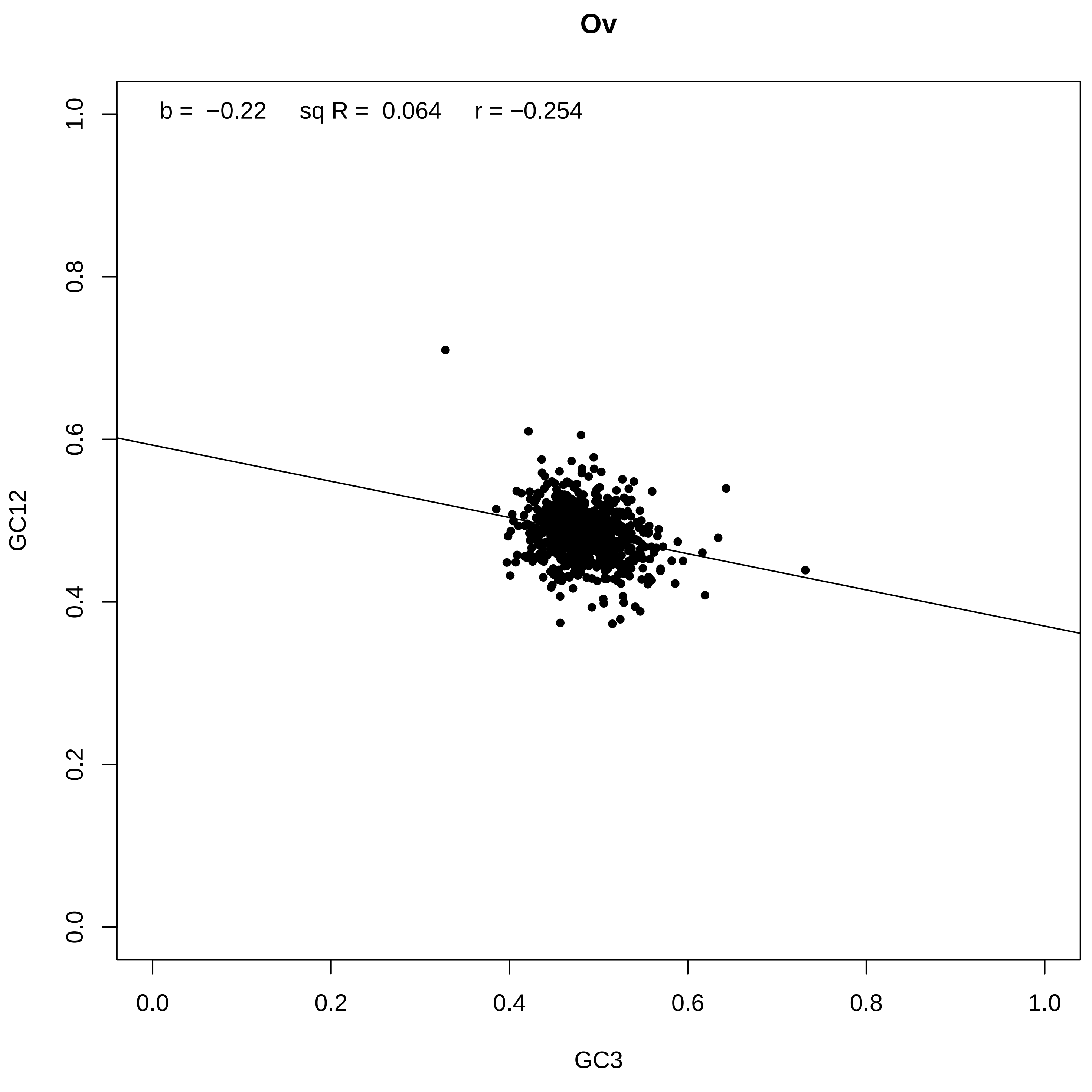
Ov
b = −0.22 sq R = 0.064 r = −0.254
GC12
GC3
0.0
0.2
0.4
0.6
0.8
1.0

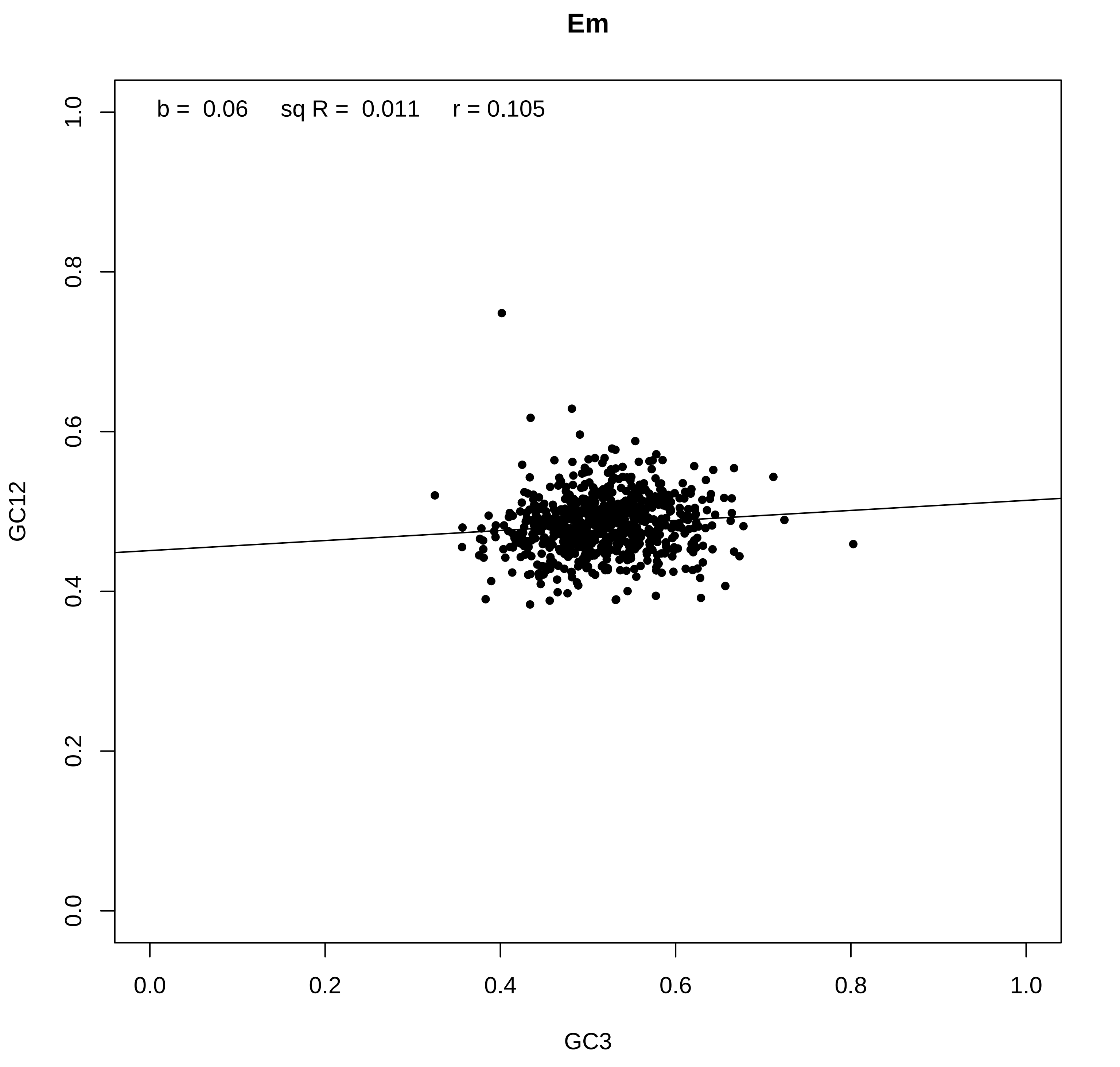

Em
b = 0.06 sq R = 0.011 r = 0.105
GC12
GC3
0.0
0.2
0.4
0.6
0.8
1.0

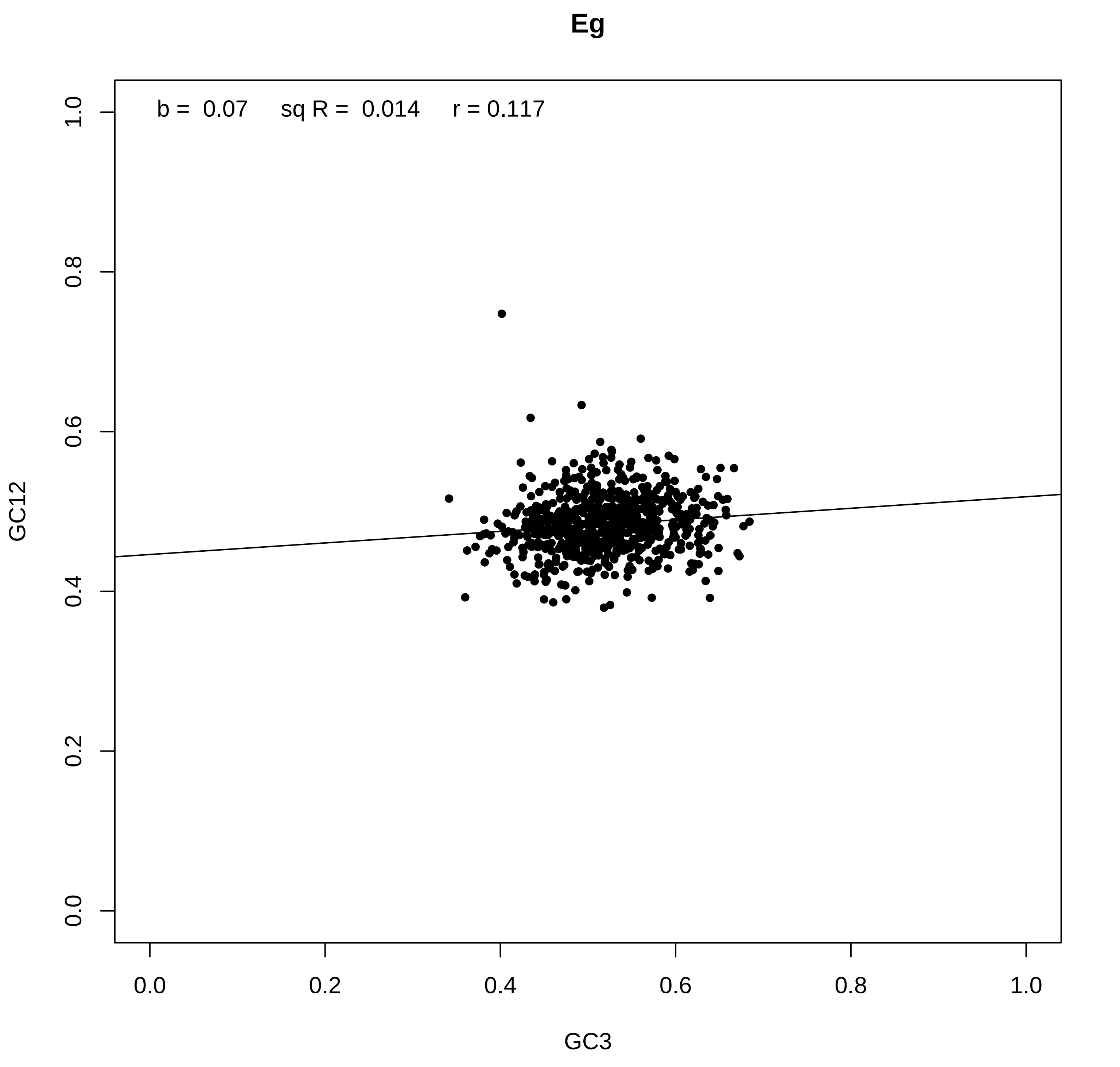
Eg
b = 0.07
sq R = 0.014
r = 0.117
GC12
GC3
0.0
0.2
0.4
0.6
0.8
1.0

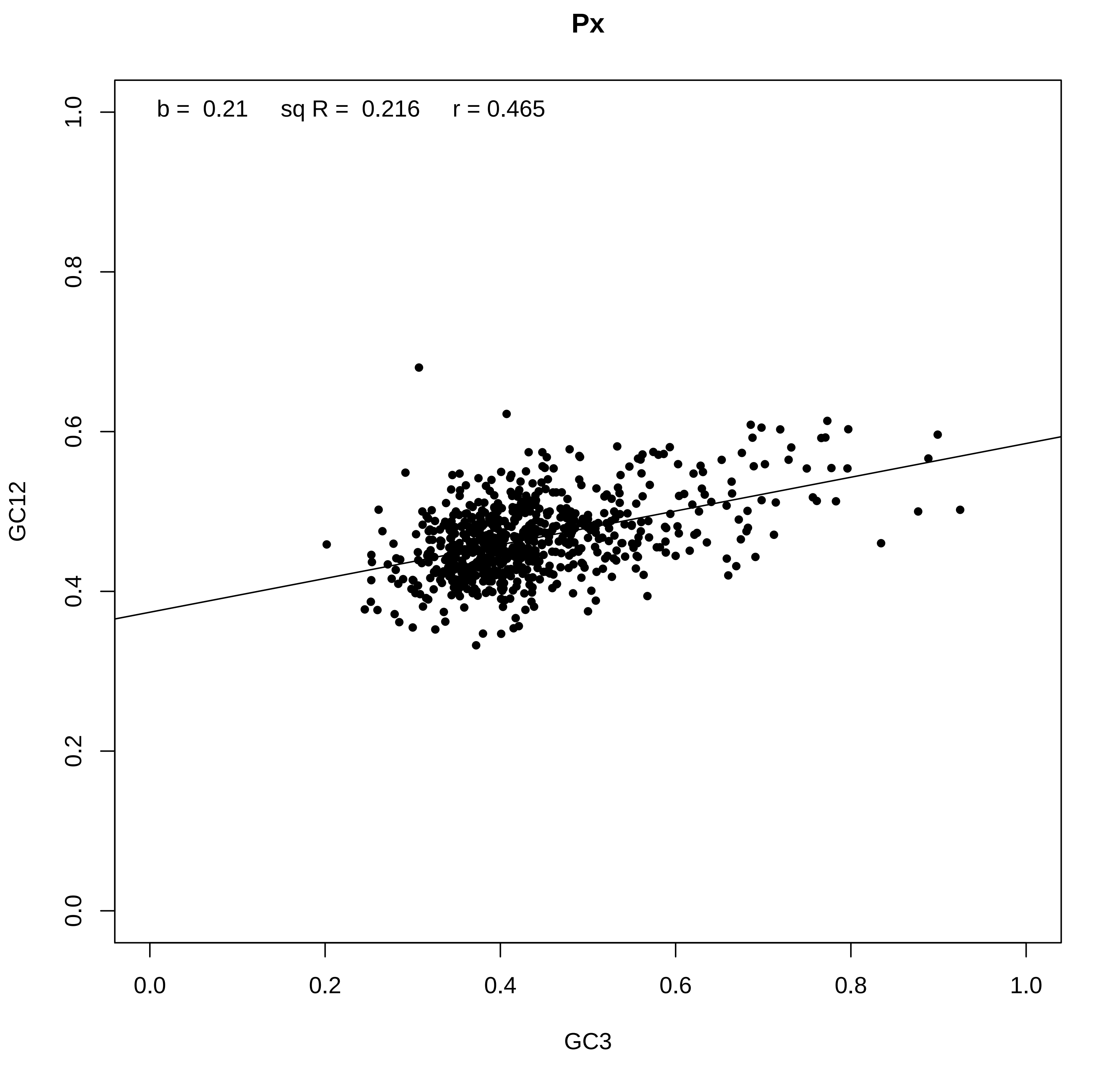
Px
b = 0.21 sq R = 0.216 r = 0.465
GC12
GC3
0.0
0.2
0.4
0.6
0.8
1.0

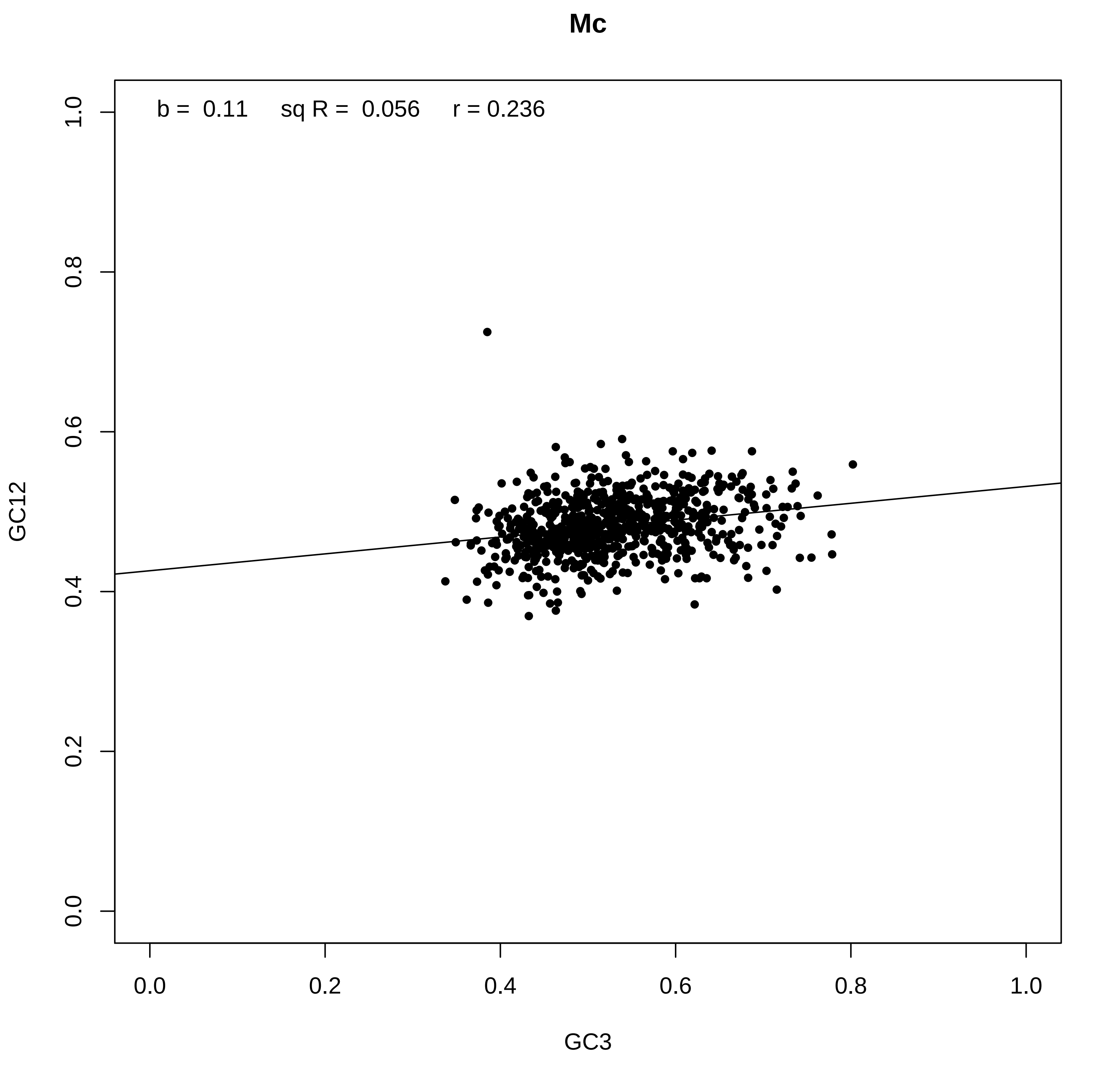

Mc
b = 0.11 sq R = 0.056 r = 0.236
GC12
GC3
0.0
0.2
0.4
0.6
0.8
1.0

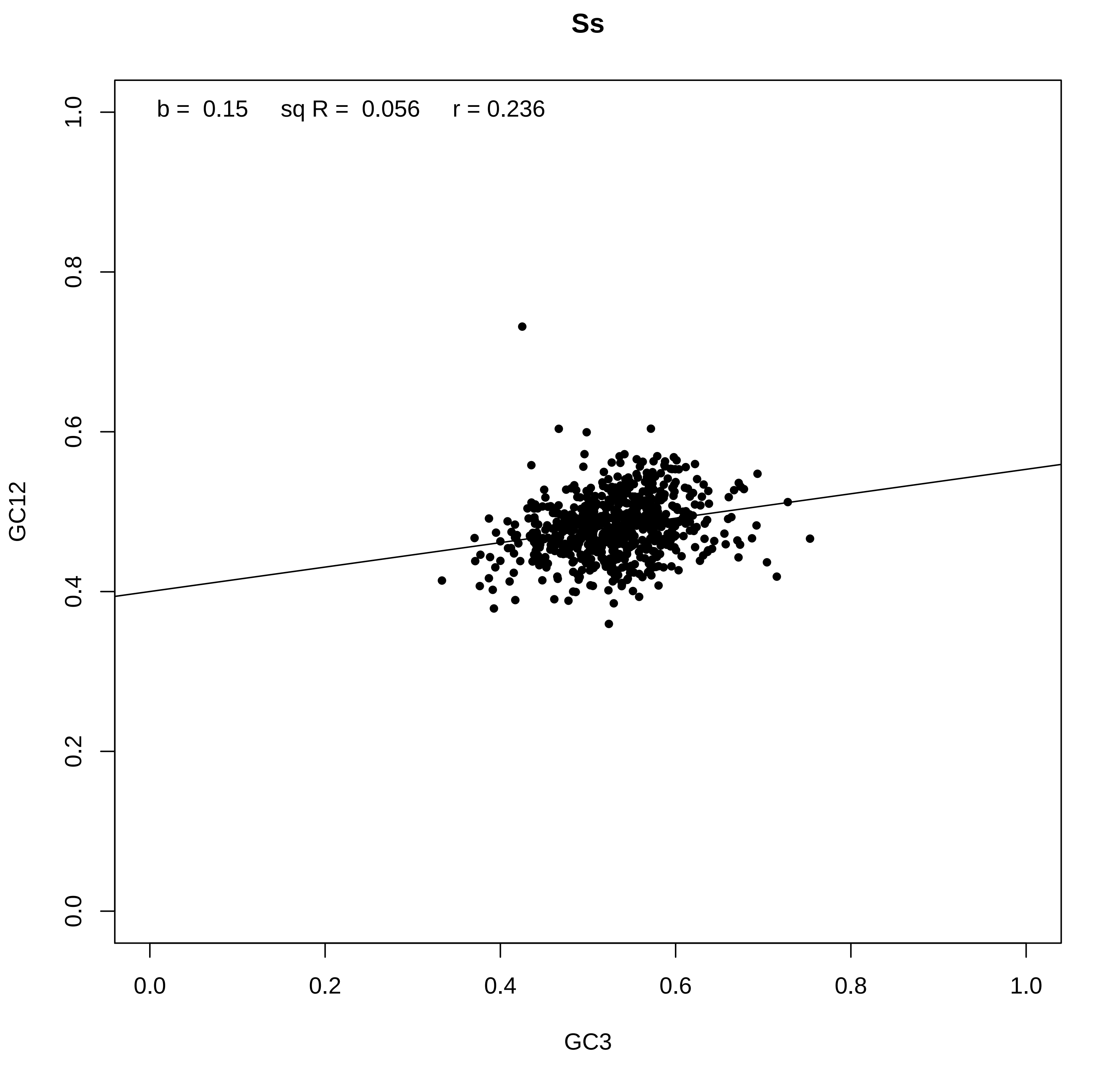
Ss
b = 0.15 sq R = 0.056 r = 0.236
GC12
GC3
0.0
0.2
0.4
0.6
0.8
1.0

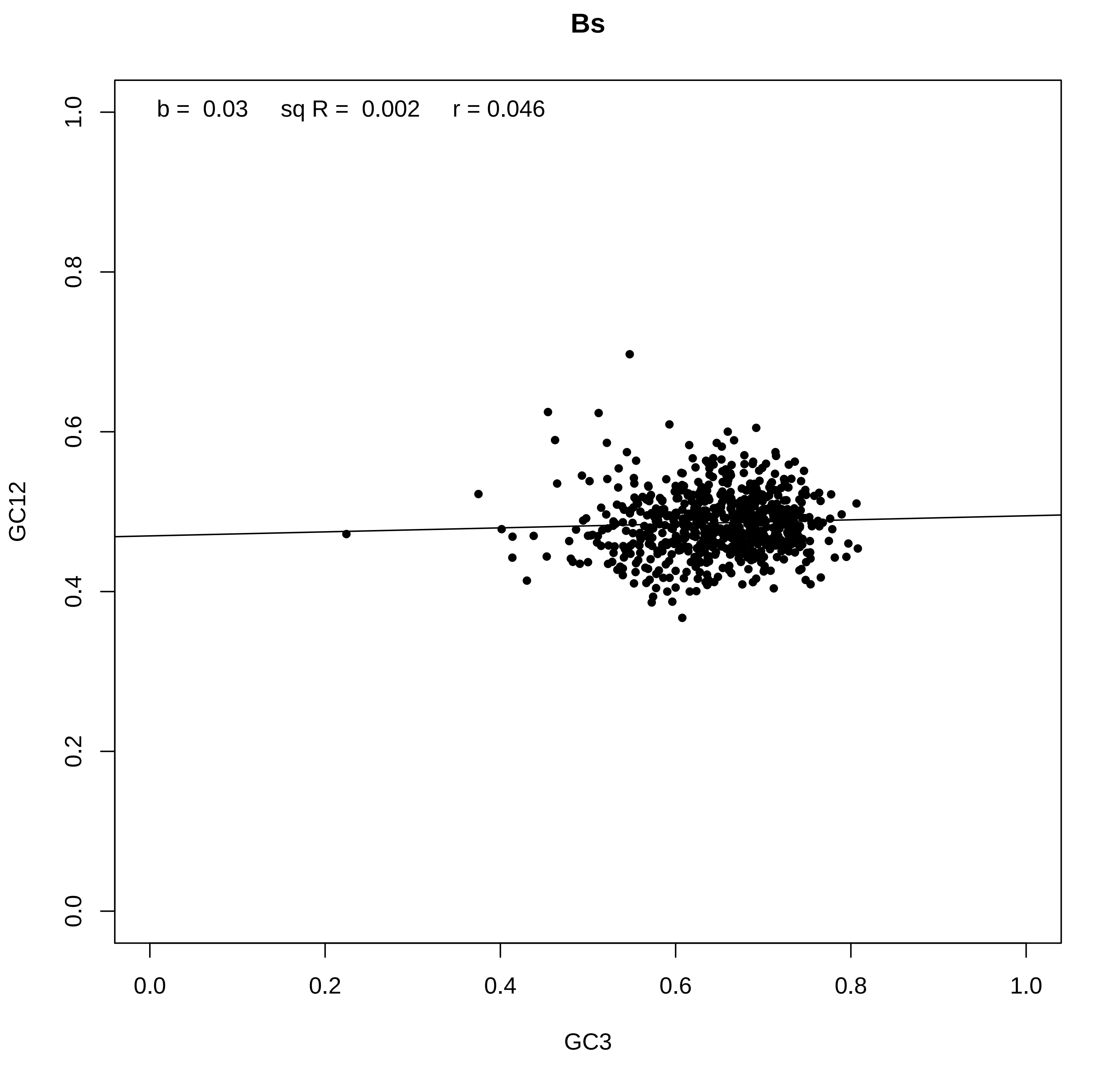
Bs
b = 0.03 sq R = 0.002 r = 0.046
GC12
GC3
0.0
0.2
0.4
0.6
0.8
1.0

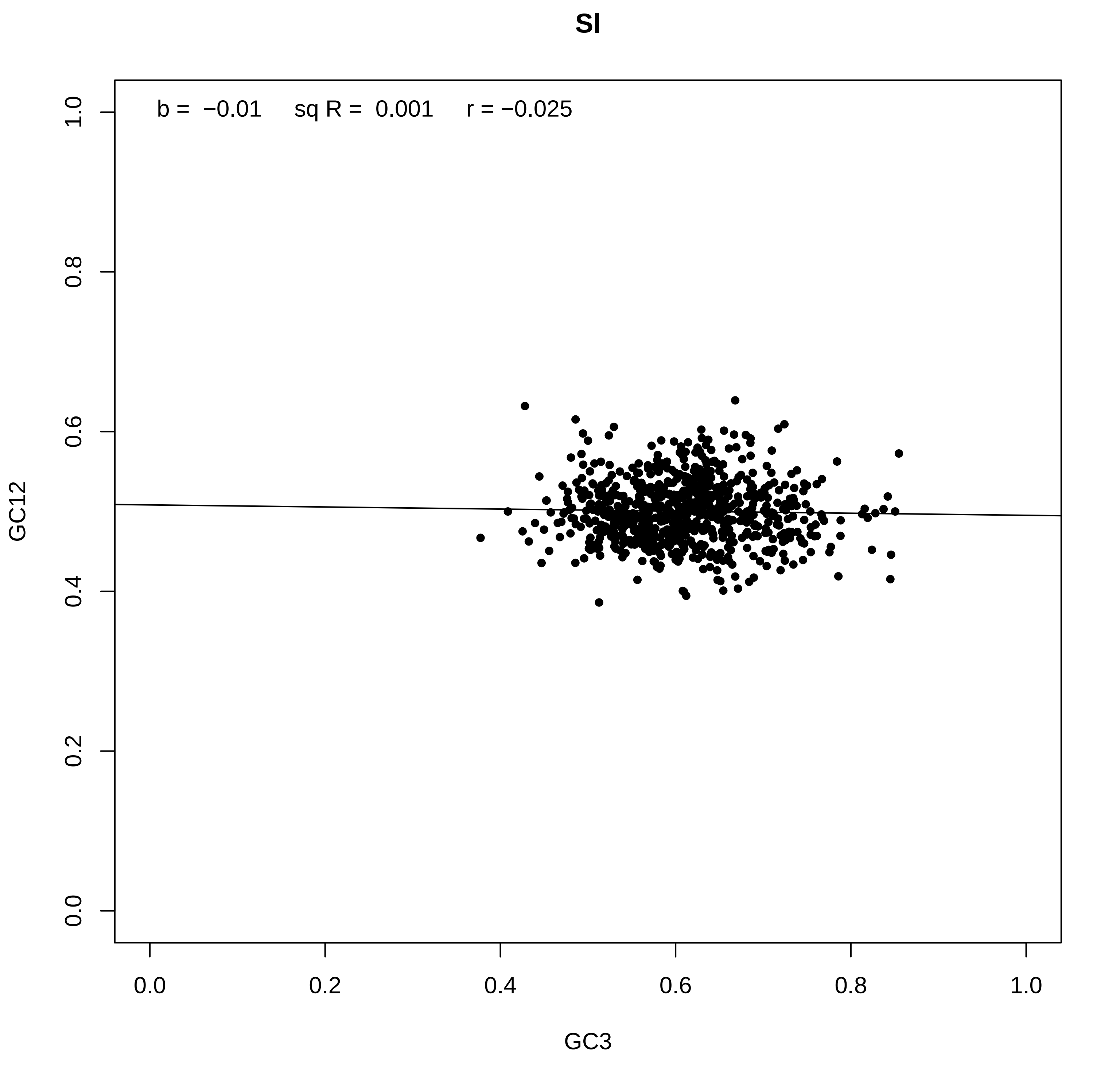

Sl
b = −0.01 sq R = 0.001 r = −0.025
GC12
GC3
0.0
0.2
0.4
0.6
0.8
1.0

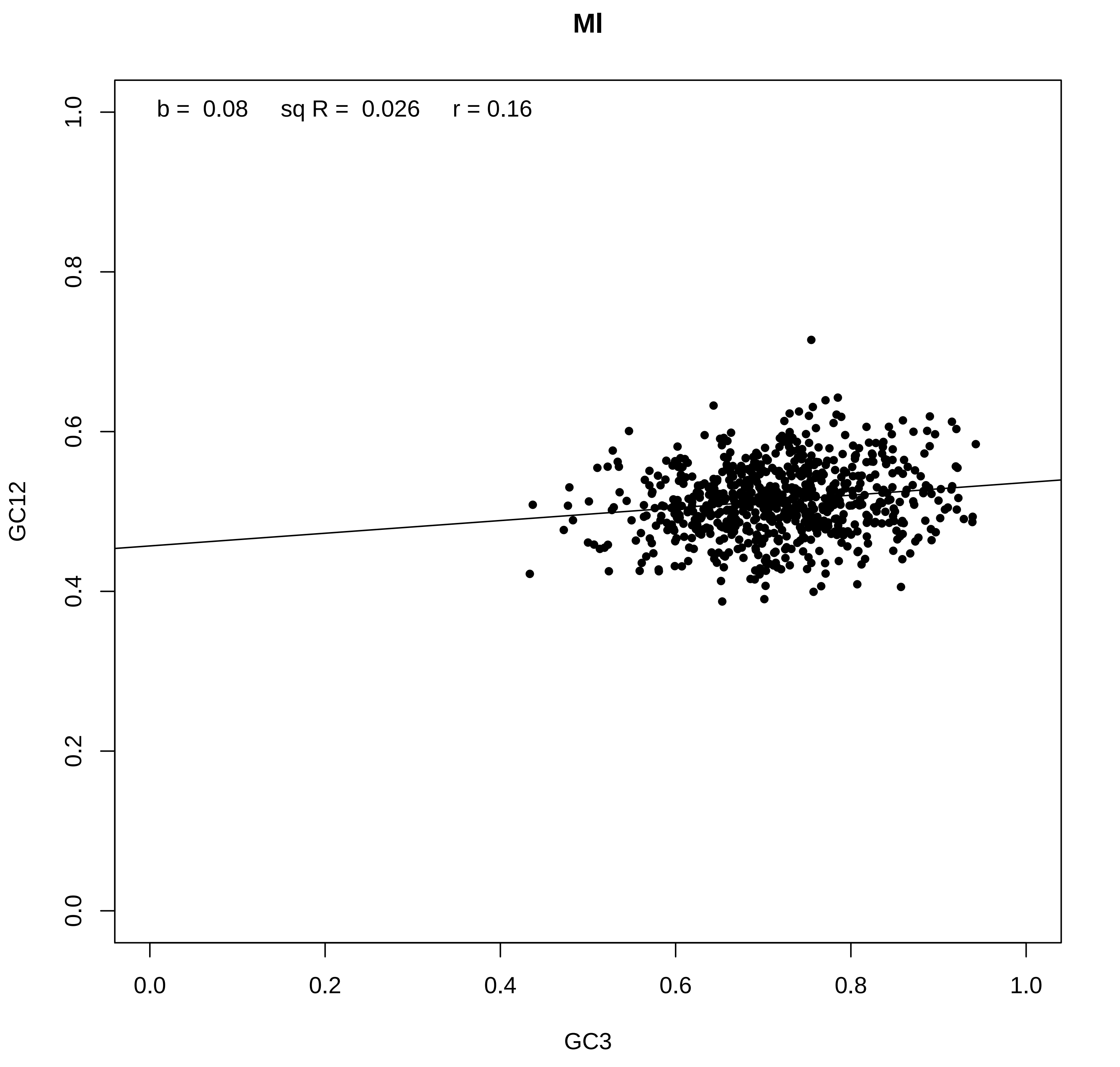

Ml
b = 0.08 sq R = 0.026 r = 0.16
GC12
GC3
0.0
0.2
0.4
0.6
0.8
1.0

SFig2. Box plots showing codon frequencies of the 22 species. Red and grey boxes shows GC and AT-ended codons, respectively. Legend in each plot indicate the species name, GC content and p-value of t test.

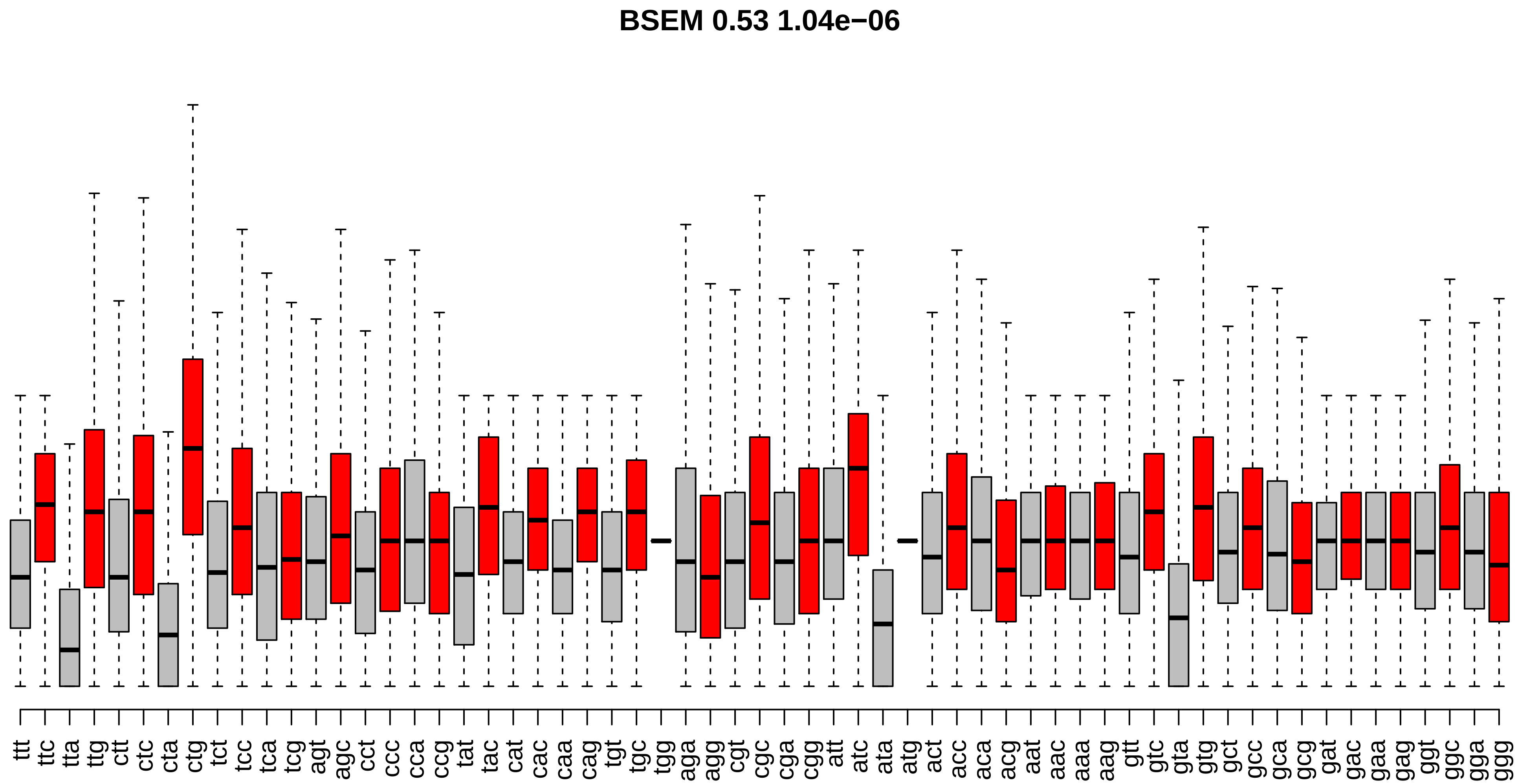

BSEM 0.53 1.04e−06
ttt ttc tta ttg ctt ctc cta ctg tct tcc tca tcg agt agc cct ccc cca ccg tat tac cat cac caa cag tgt tgc tgg aga agg cgt cgc cga cgg att atc ata atg act acc aca acg aat aac aaa aag gtt gtc gta gtg gct gcc gca gcg gat gac gaa gag ggt ggc gga ggg

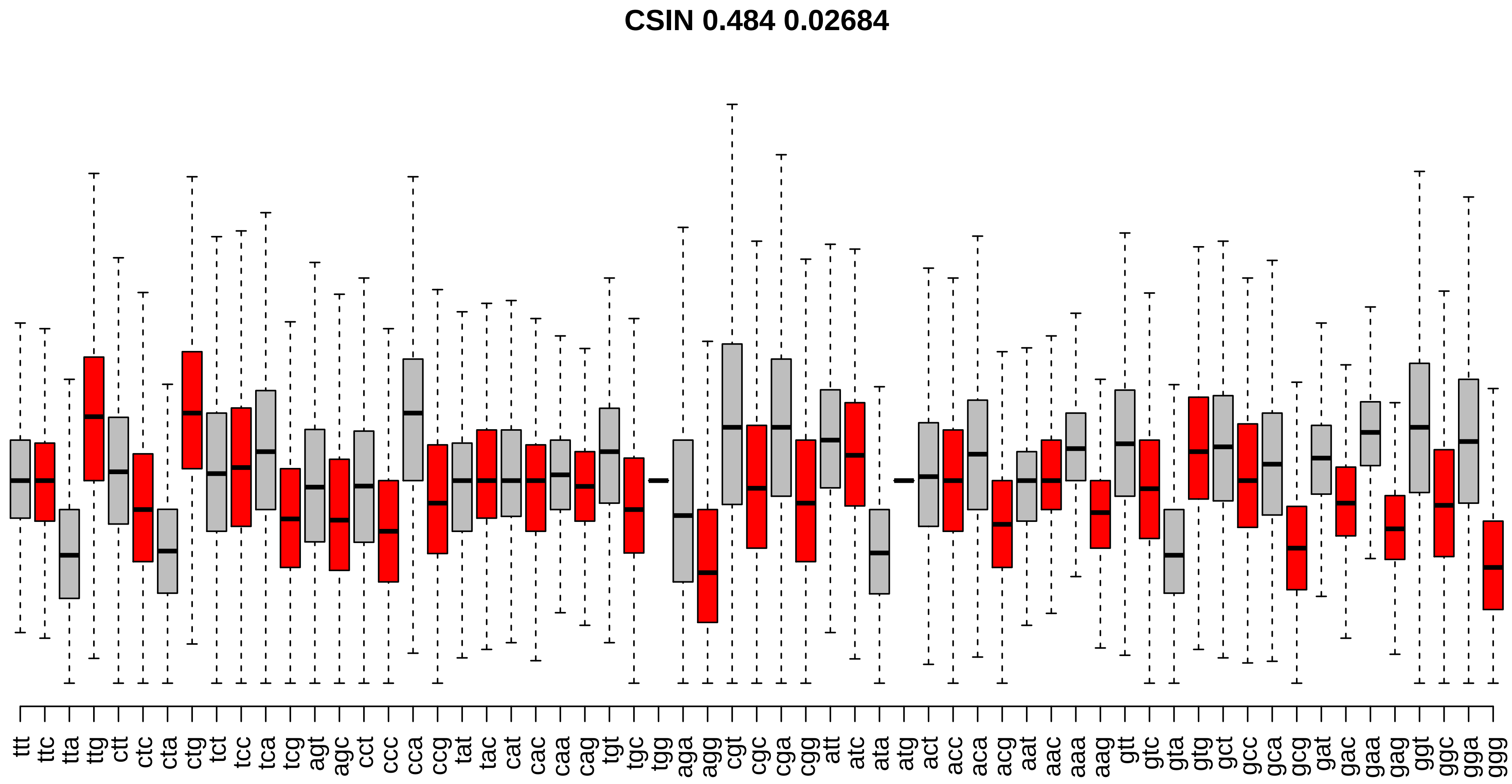
CSIN 0.484 0.02684
ttt
ttc
tta
ttg
ctt
ctc
cta
ctg
tct
tcc
tca
tcg
agt
agc
cct
ccc
cca
ccg
tat
tac
cat
cac
caa
cag
tgt
tgc
tgg
aga
agg
cgt
cgc
cga
cgg
att
atc
ata
atg
act
acc
aca
acg
aat
aac
aaa
aag
gtt
gtc
gta
gtg
gct
gcc
gca
gcg
gat
gac
gaa
gag
ggt
ggc
gga
ggg

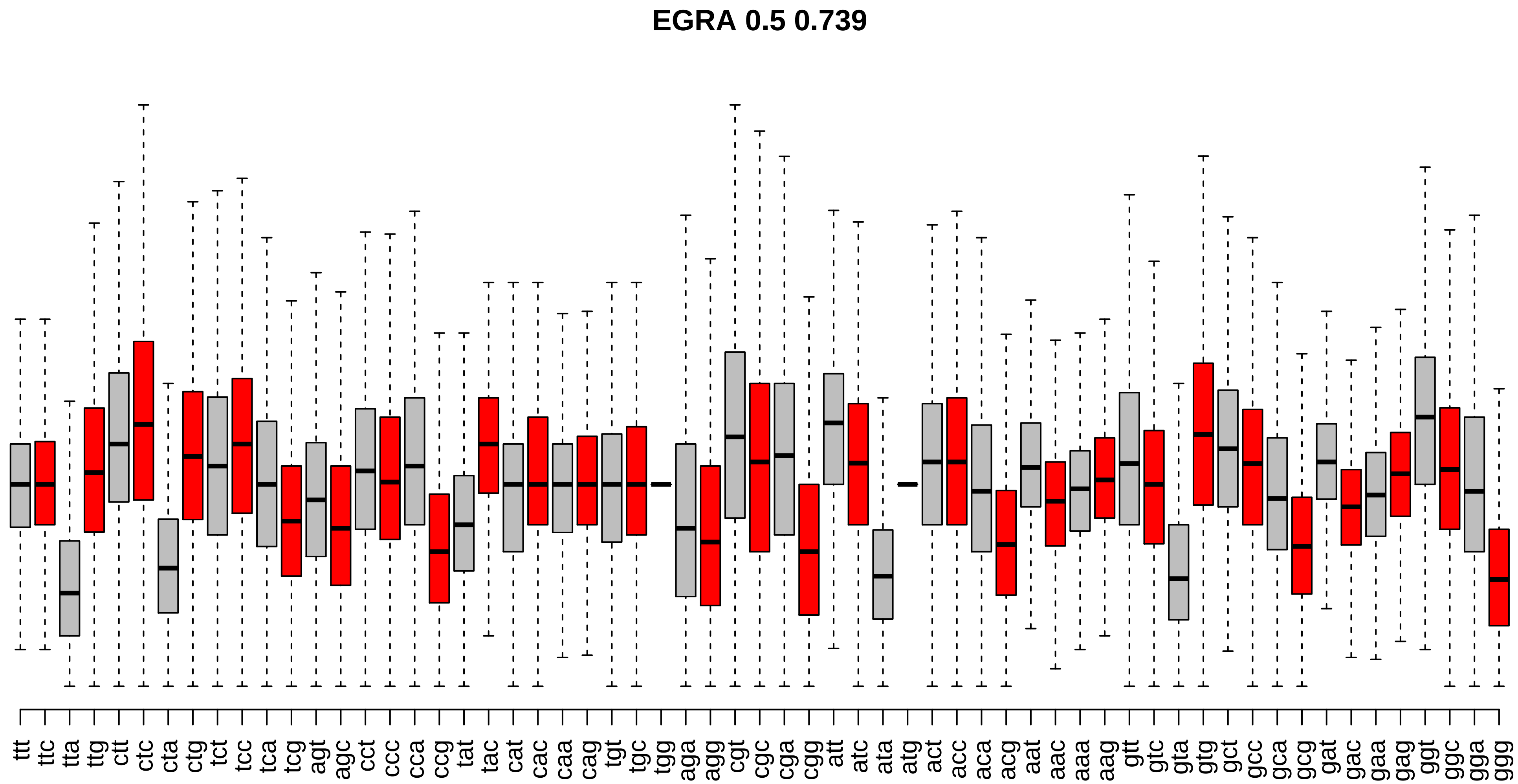
EGRA 0.5 0.739
ttt ttc tta ttg ctt ctc cta ctg tct tcc tca tcg agt agc cct ccc cca ccg tat tac cat cac caa cag tgt tgc tgg aga agg cgt cgc cga cgg att atc ata atg act acc aca acg aat aac aaa aag gtt gtc gta gtg gct gcc gca gcg gat gac gaa gag ggt ggc gga ggg

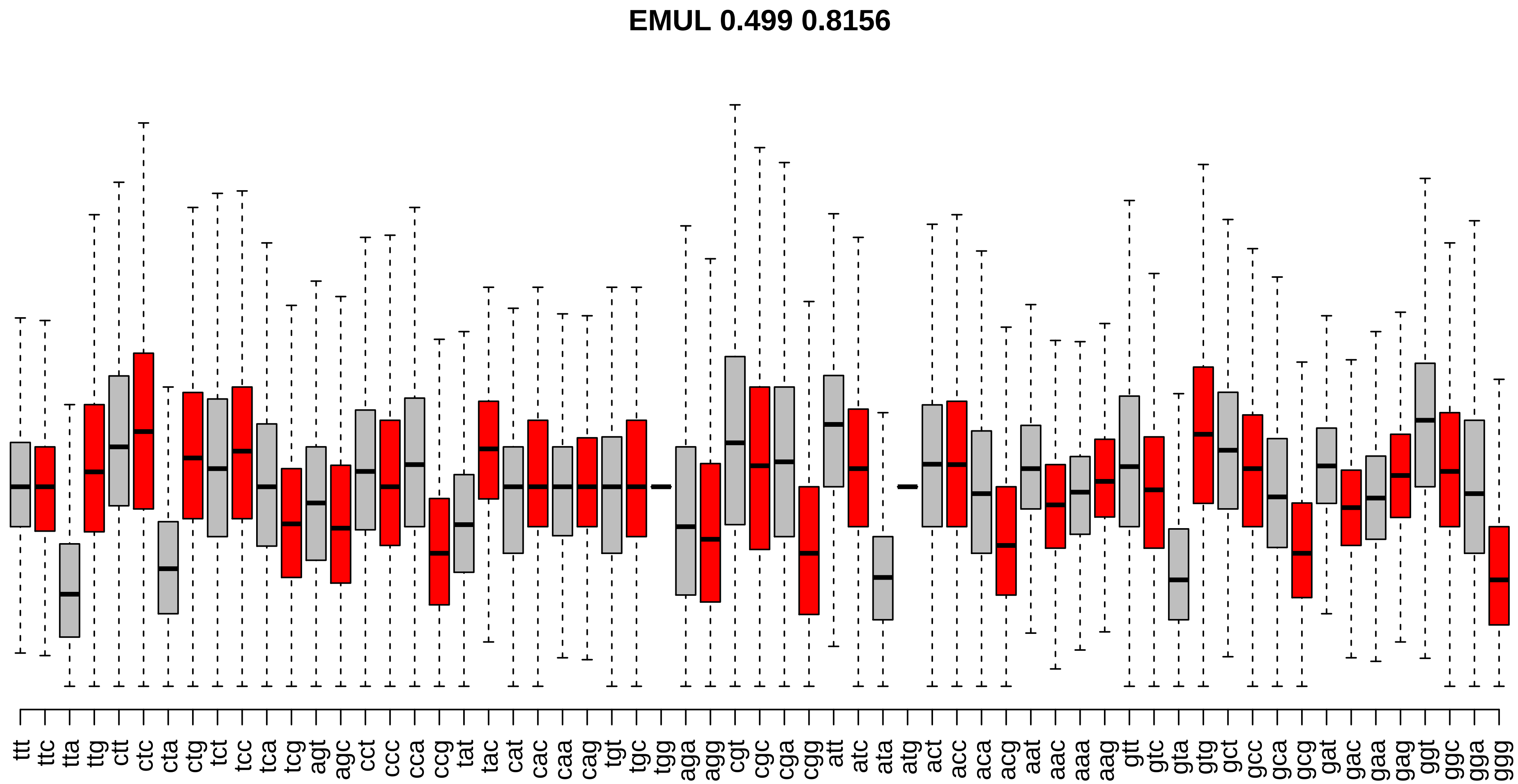
EMUL 0.499 0.8156
ttt ttc tta ttg ctt ctc cta ctg tct tcc tca tcg agt agc cct ccc cca ccg tat tac cat cac caa cag tgt tgc tgg aga agg cgt cgc cga cgg att atc ata atg act acc aca acg aat aac aaa aag gtt gtc gta gtg gct gcc gca gcg gat gac gaa gag ggt ggc gga ggg

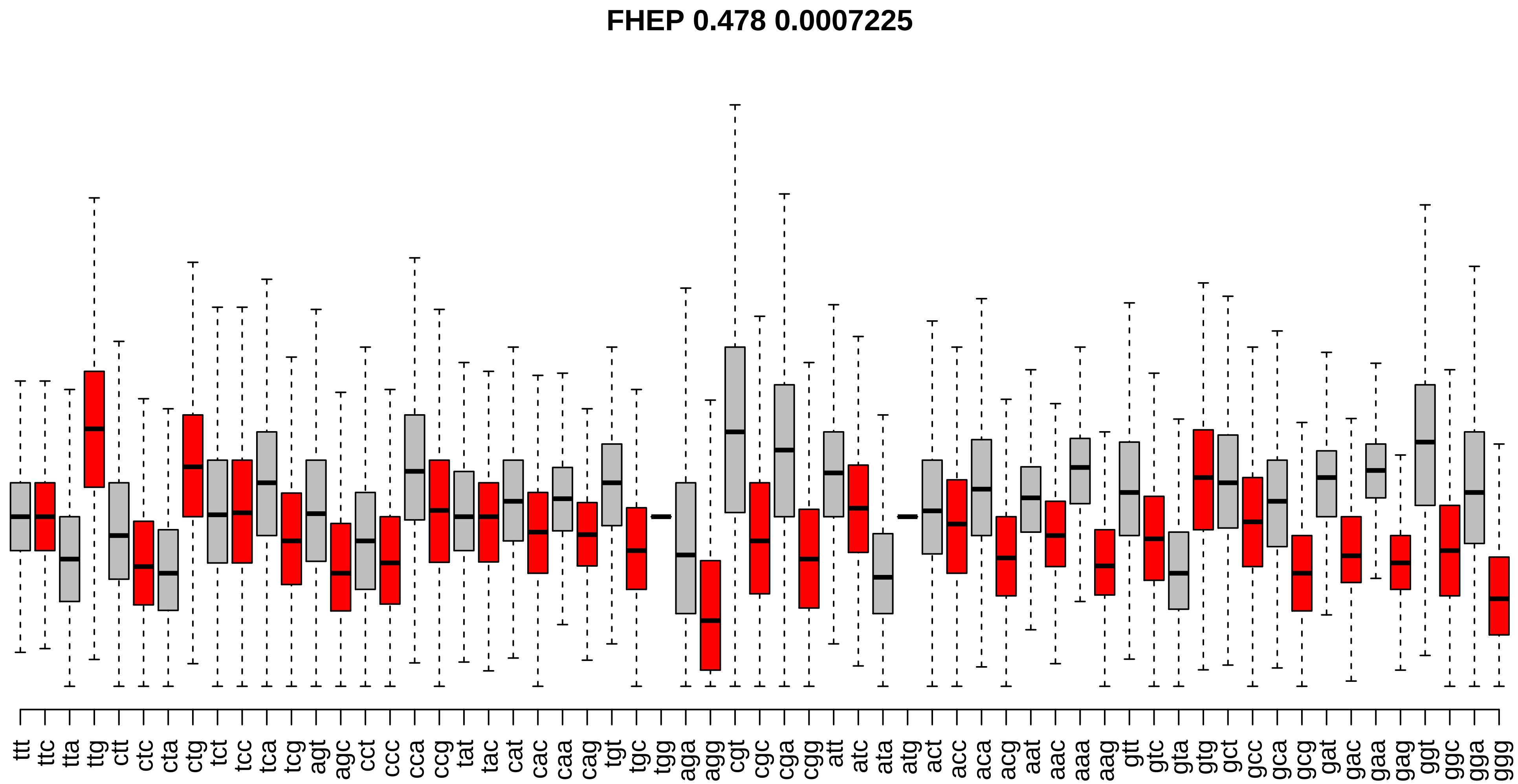
FHEP 0.478 0.0007225
ttt ttc tta ttg ctt ctc cta ctg tct tcc tca tcg agt agc cct ccc cca ccg tat tac cat cac caa cag tgt tgc tgg aga agg cgt cgc cga cgg att atc ata atg act acc aca acg aat aac aaa aag gtt gtc gta gtg gct gcc gca gcg gat gac gaa gag ggt ggc gga ggg

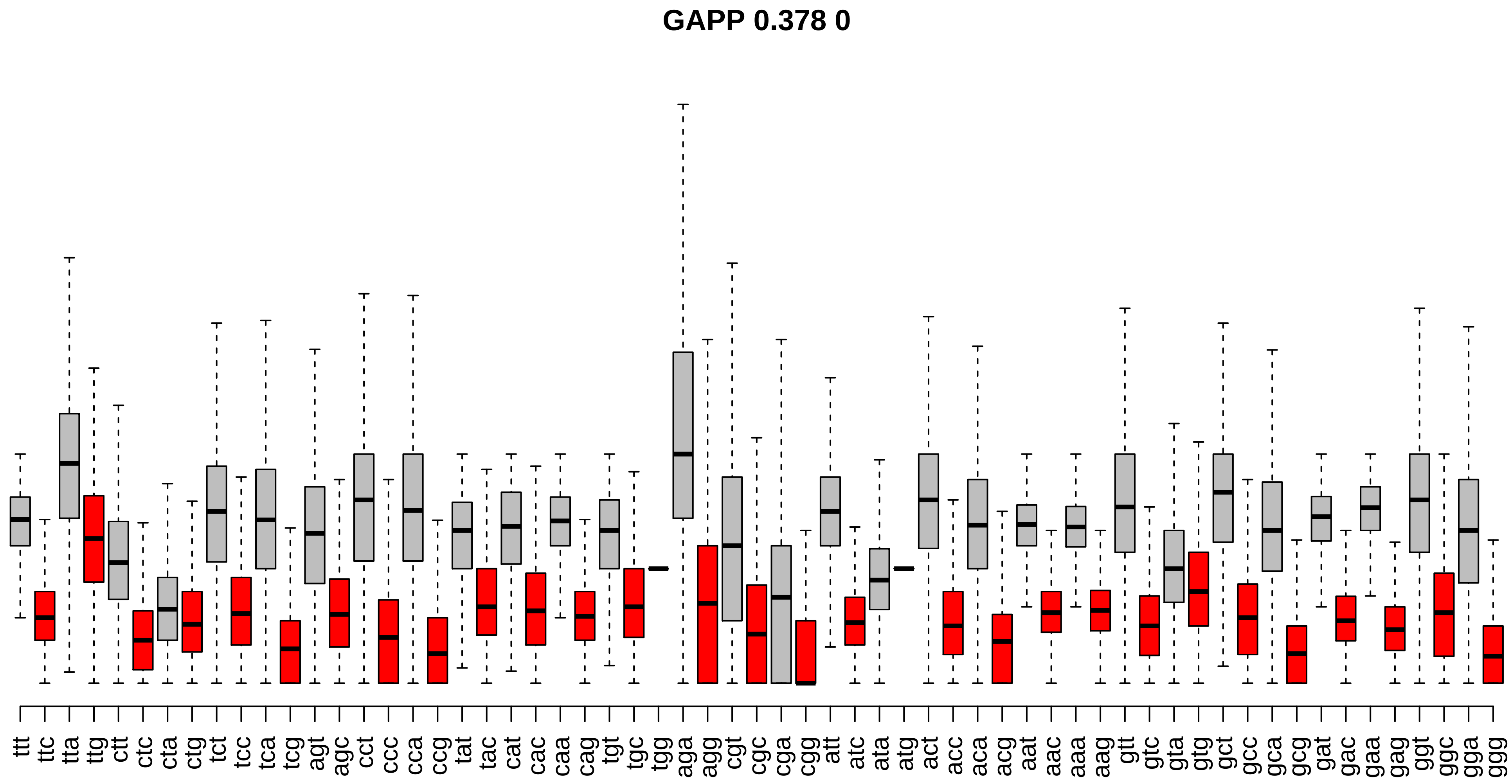
GAPP 0.378 0
ttt
ttc
tta
ttg
ctt
ctc
cta
ctg
tct
tcc
tca
tcg
agt
agc
cct
ccc
cca
ccg
tat
tac
cat
cac
caa
cag
tgt
tgc
tgg
aga
agg
cgt
cgc
cga
cgg
att
atc
ata
atg
act
acc
aca
acg
aat
aac
aaa
aag
gtt
gtc
gta
gtg
gct
gcc
gca
gcg
gat
gac
gaa
gag
ggt
ggc
gga
ggg

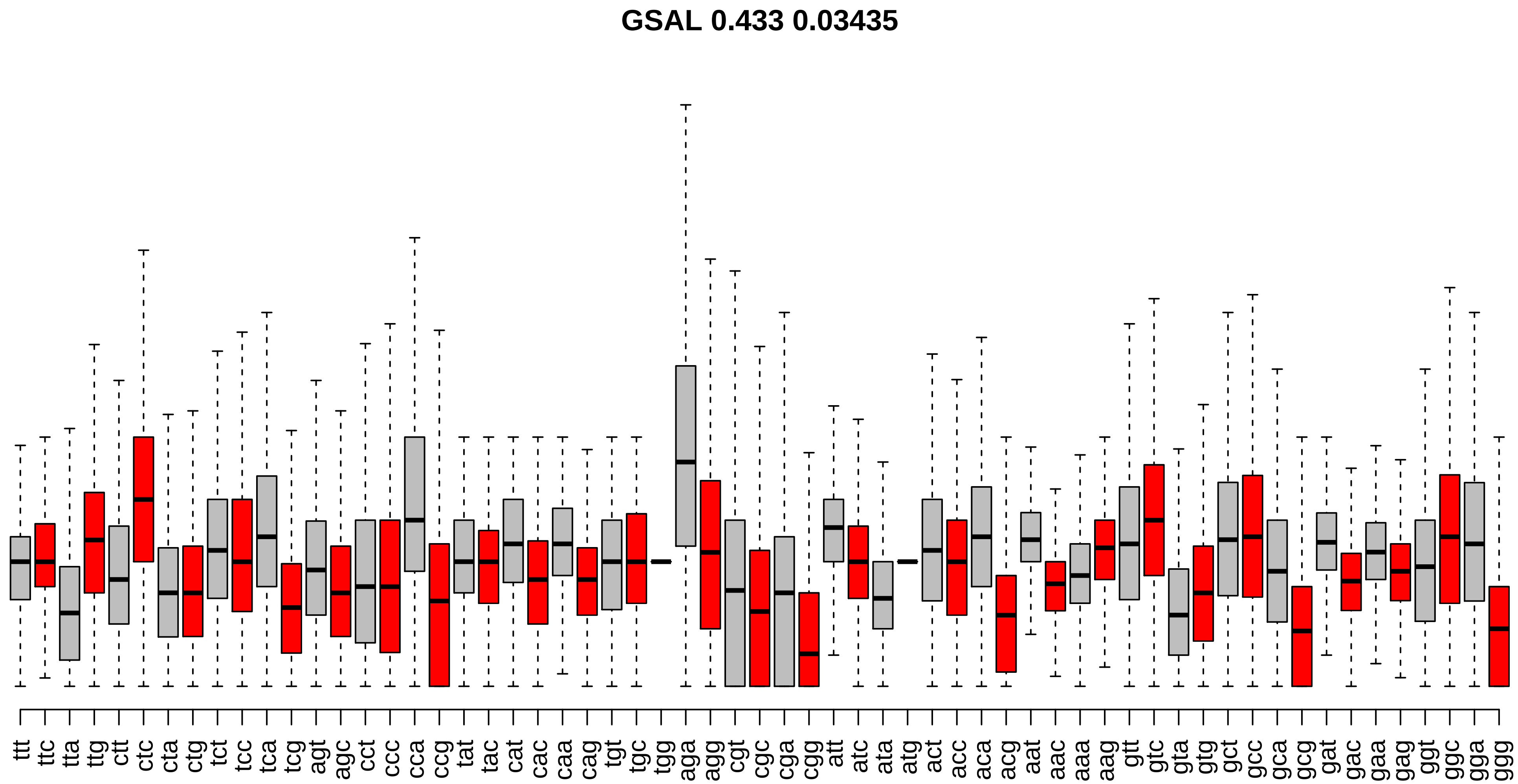
GSAL 0.433 0.03435
ttt ttc tta ttg ctt ctc cta ctg tct tcc tca tcg agt agc cct ccc cca ccg tat tac cat cac caa cag tgt tgc tgg aga agg cgt cgc cga cgg att atc ata atg act acc aca acg aat aac aaa aag gtt gtc gta gtg gct gcc gca gcg gat gac gaa gag ggt ggc gga ggg

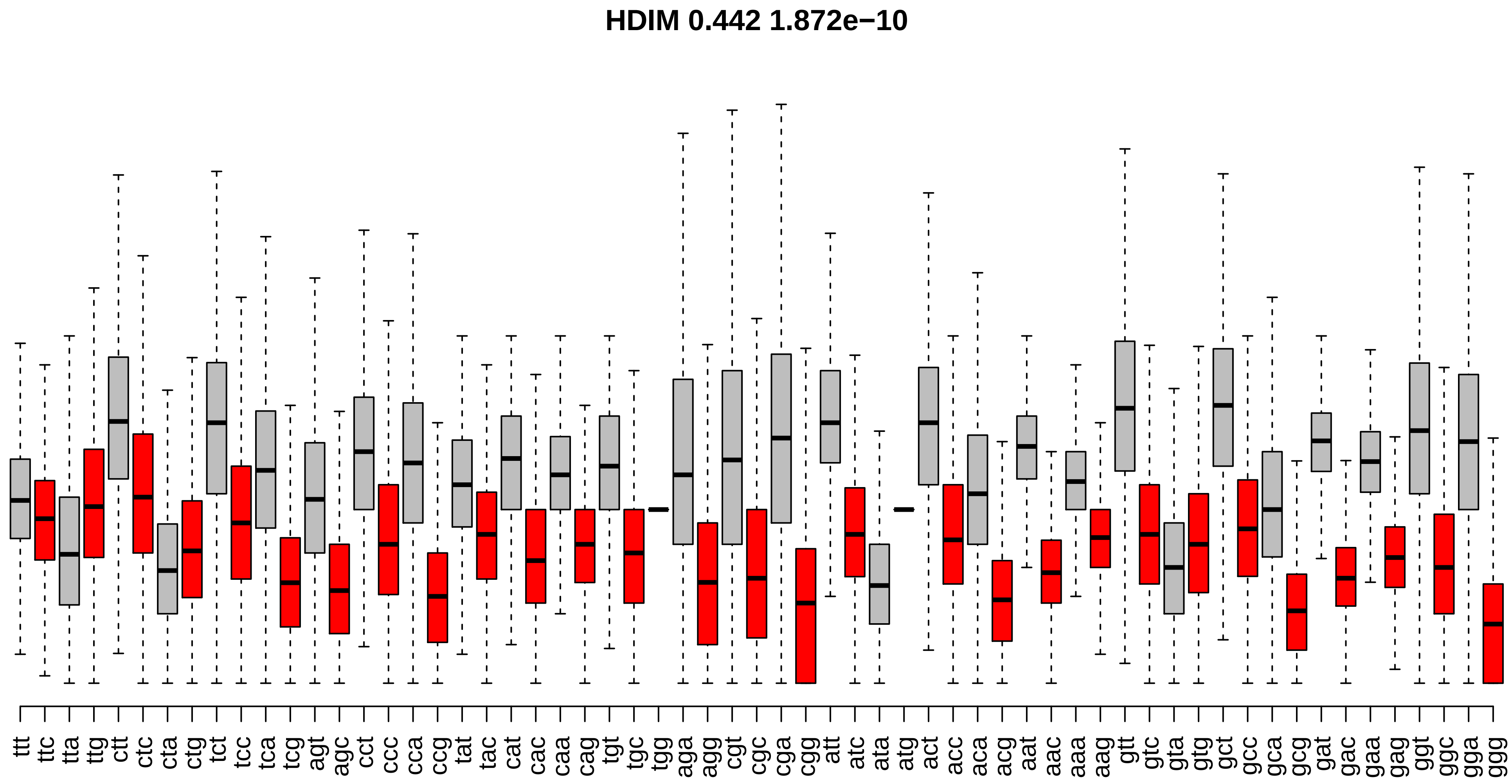
HDIM 0.442 1.872e−10
ttt
ttc
tta
ttg
ctt
ctc
cta
ctg
tct
tcc
tca
tcg
agt
agc
cct
ccc
cca
ccg
tat
tac
cat
cac
caa
cag
tgt
tgc
tgg
aga
agg
cgt
cgc
cga
cgg
att
atc
ata
atg
act
acc
aca
acg
aat
aac
aaa
aag
gtt
gtc
gta
gtg
gct
gcc
gca
gcg
gat
gac
gaa
gag
ggt
ggc
gga
ggg

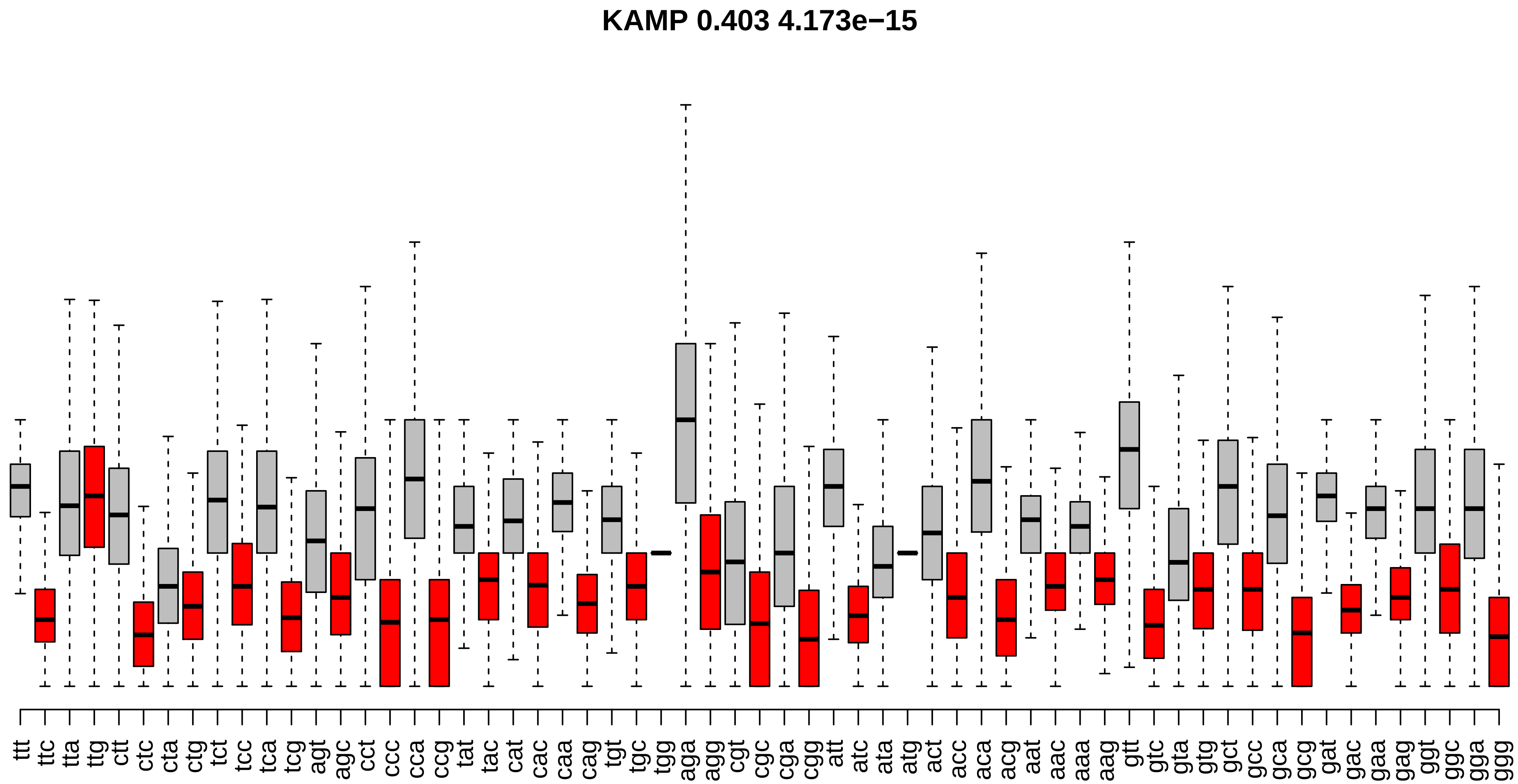

KAMP 0.403 4.173e−15
ttt ttc tta ttg ctt ctc cta ctg tct tcc tca tcg agt agc cct ccc cca ccg tat tac cat cac caa cag tgt tgc tgg aga agg cgt cgc cga cgg att atc ata atg act acc aca acg aat aac aaa aag gtt gtc gta gtg gct gcc gca gcg gat gac gaa gag ggt ggc gga ggg

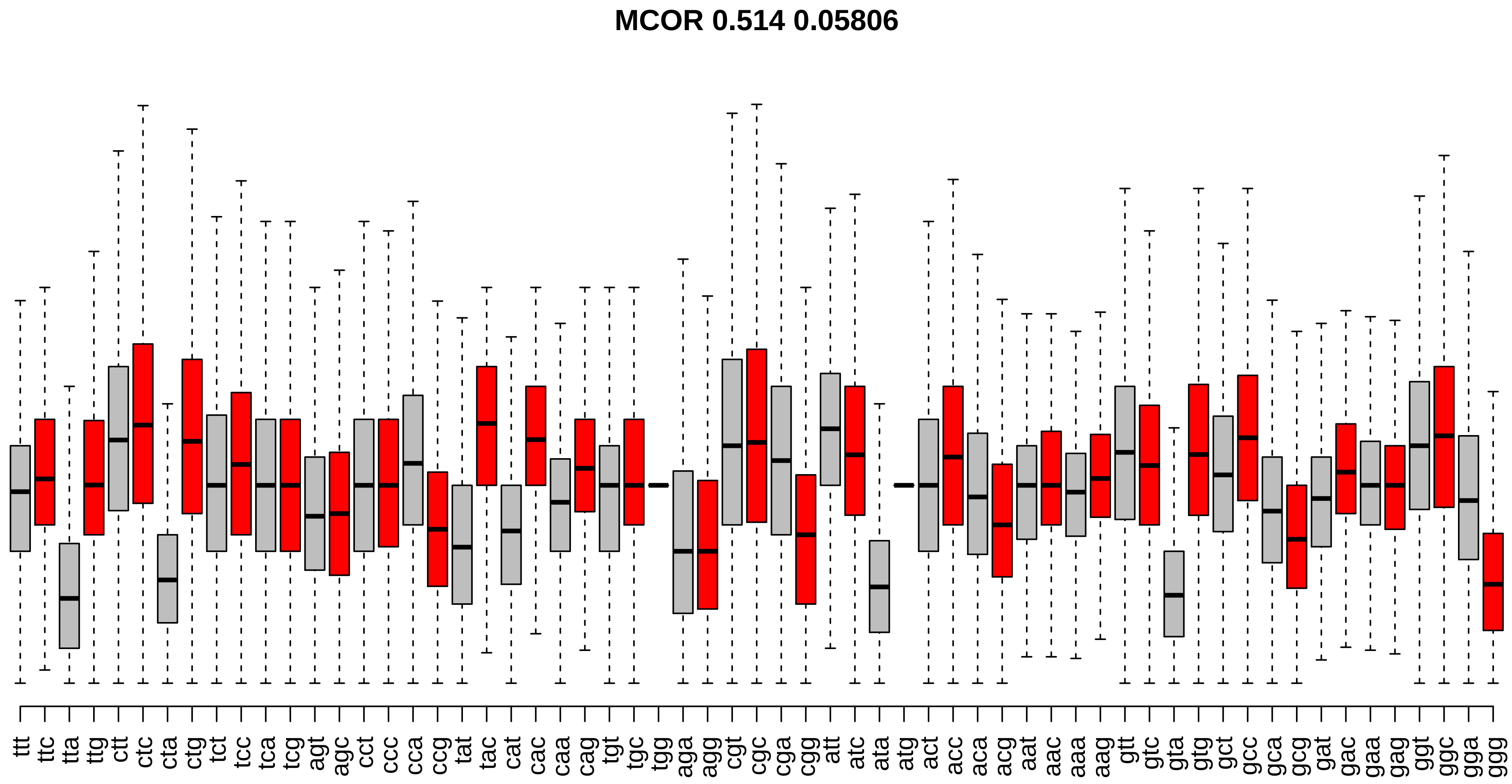
MCOR 0.514 0.05806
ttt
ttc
tta
ttg
ctt
ctc
cta
ctg
tct
tcc
tca
tcg
agt
agc
cct
ccc
cca
ccg
tat
tac
cat
cac
caa
cag
tgt
tgc
tgg
aga
agg
cgt
cgc
cga
cgg
att
atc
ata
atg
act
acc
aca
acg
aat
aac
aaa
aag
gtt
gtc
gta
gtg
gct
gcc
gca
gcg
gat
gac
gaa
gag
ggt
ggc
gga
ggg

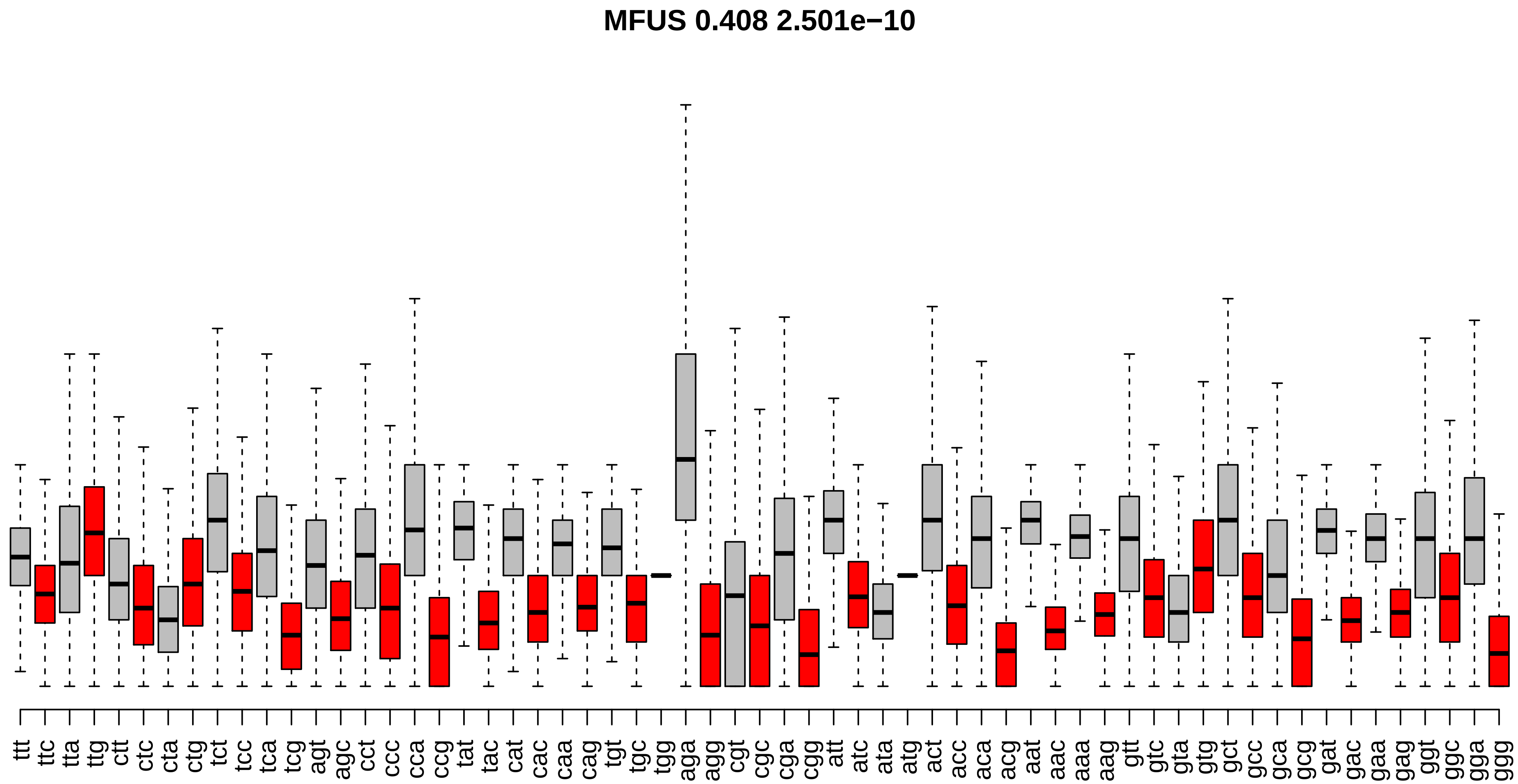

MFUS 0.408 2.501e−10
ttt
ttc
tta
ttg
ctt
ctc
cta
ctg
tct
tcc
tca
tcg
agt
agc
cct
ccc
cca
ccg
tat
tac
cat
cac
caa
cag
tgt
tgc
tgg
aga
agg
cgt
cgc
cga
cgg
att
atc
ata
atg
act
acc
aca
acg
aat
aac
aaa
aag
gtt
gtc
gta
gtg
gct
gcc
gca
gcg
gat
gac
gaa
gag
ggt
ggc
gga
ggg

MLIG 0.587 1.87e−08

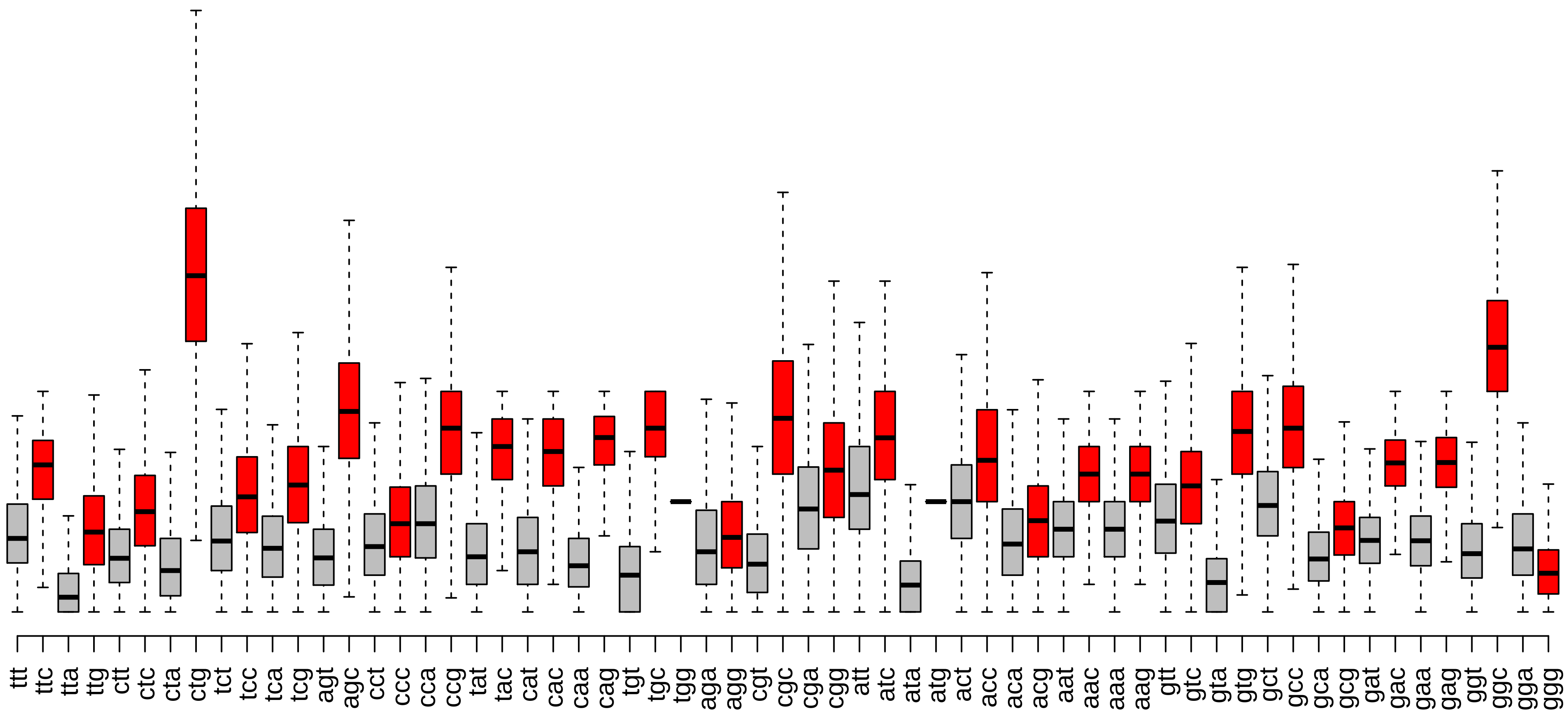

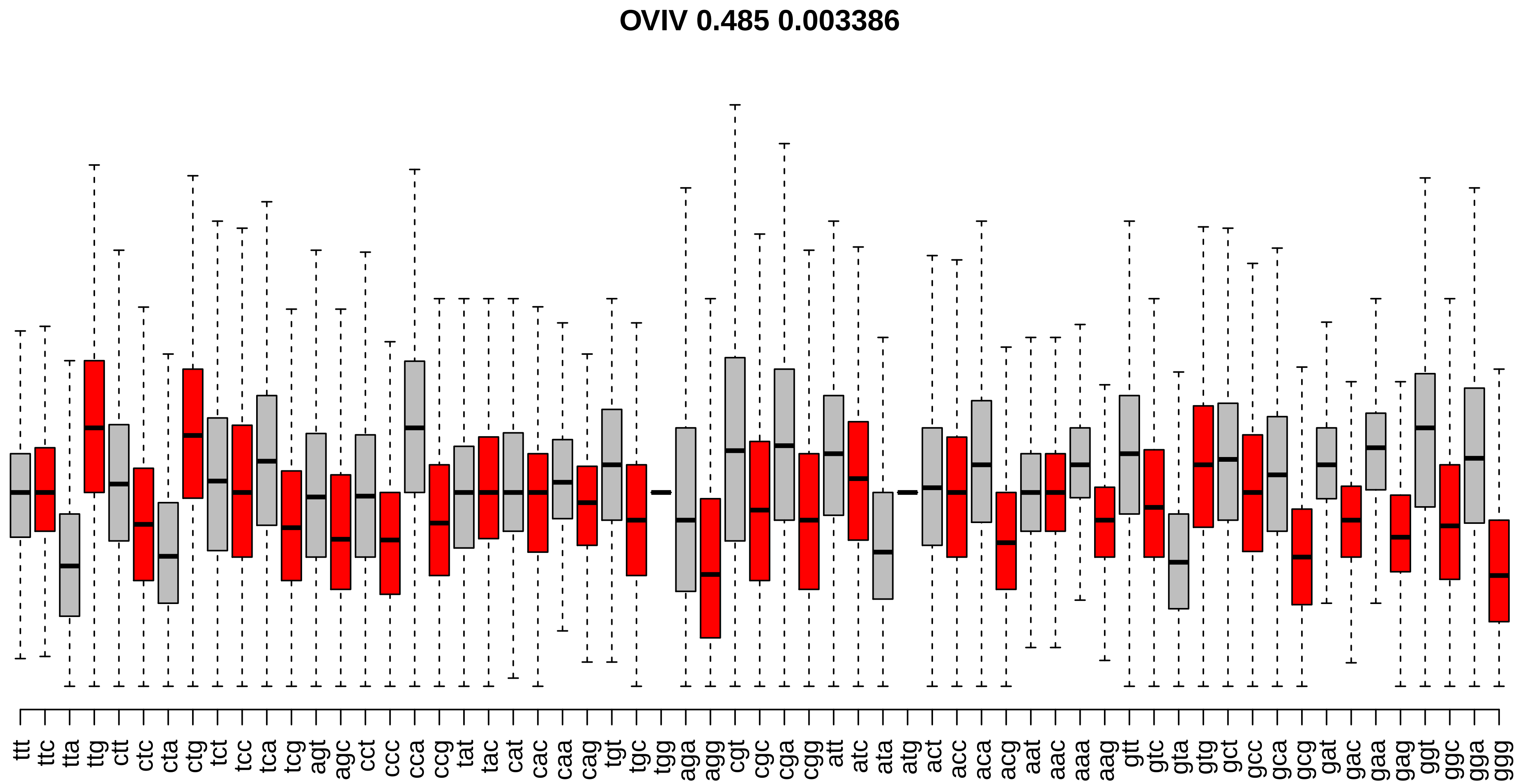

OVIV 0.485 0.003386
ttt
ttc
tta
ttg
ctt
ctc
cta
ctg
tct
tcc
tca
tcg
agt
agc
cct
ccc
cca
ccg
tat
tac
cat
cac
caa
cag
tgt
tgc
tgg
aga
agg
cgt
cgc
cga
cgg
att
atc
ata
atg
act
acc
aca
acg
aat
aac
aaa
aag
gtt
gtc
gta
gtg
gct
gcc
gca
gcg
gat
gac
gaa
gag
ggt
ggc
gga
ggg

PVIT 0.464 0.00565

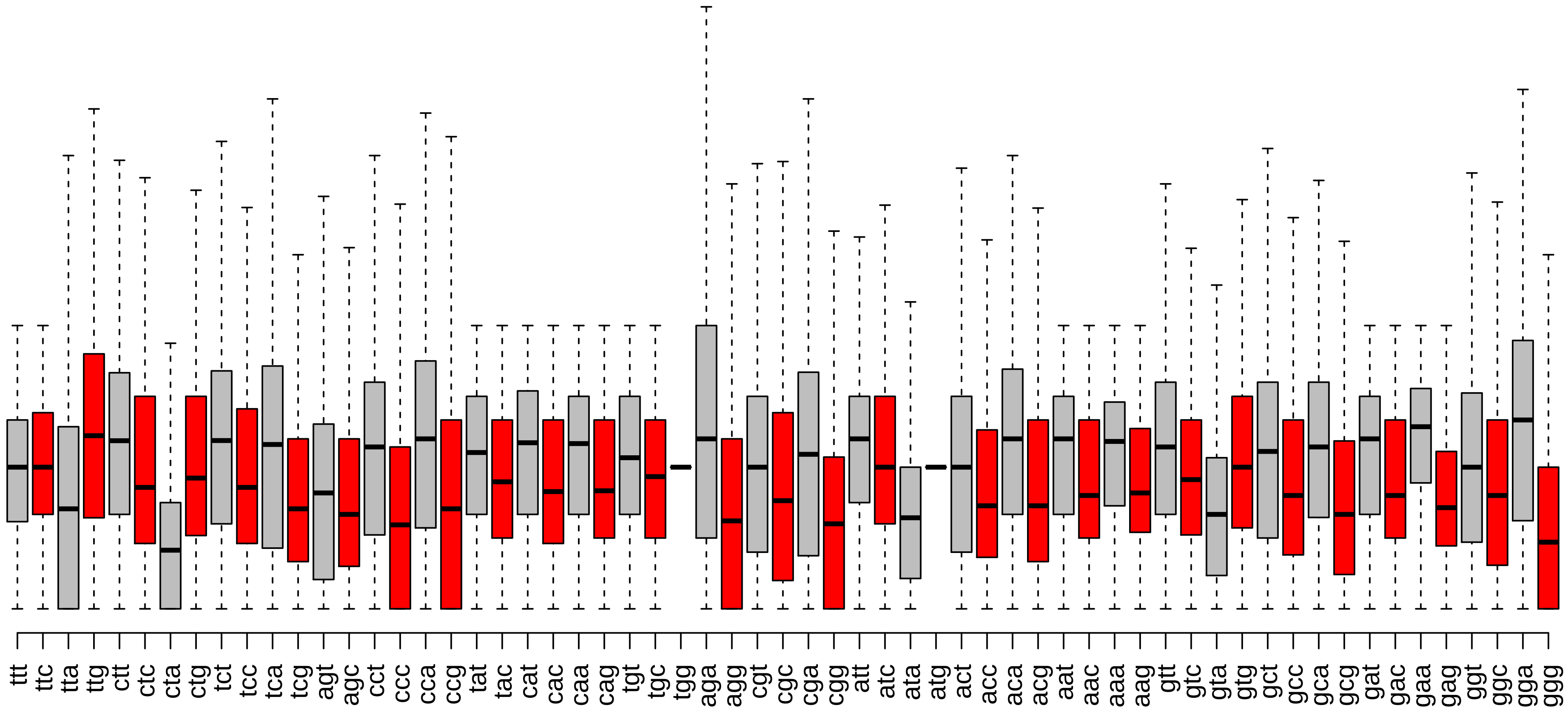

PXEN 0.502 0.08709

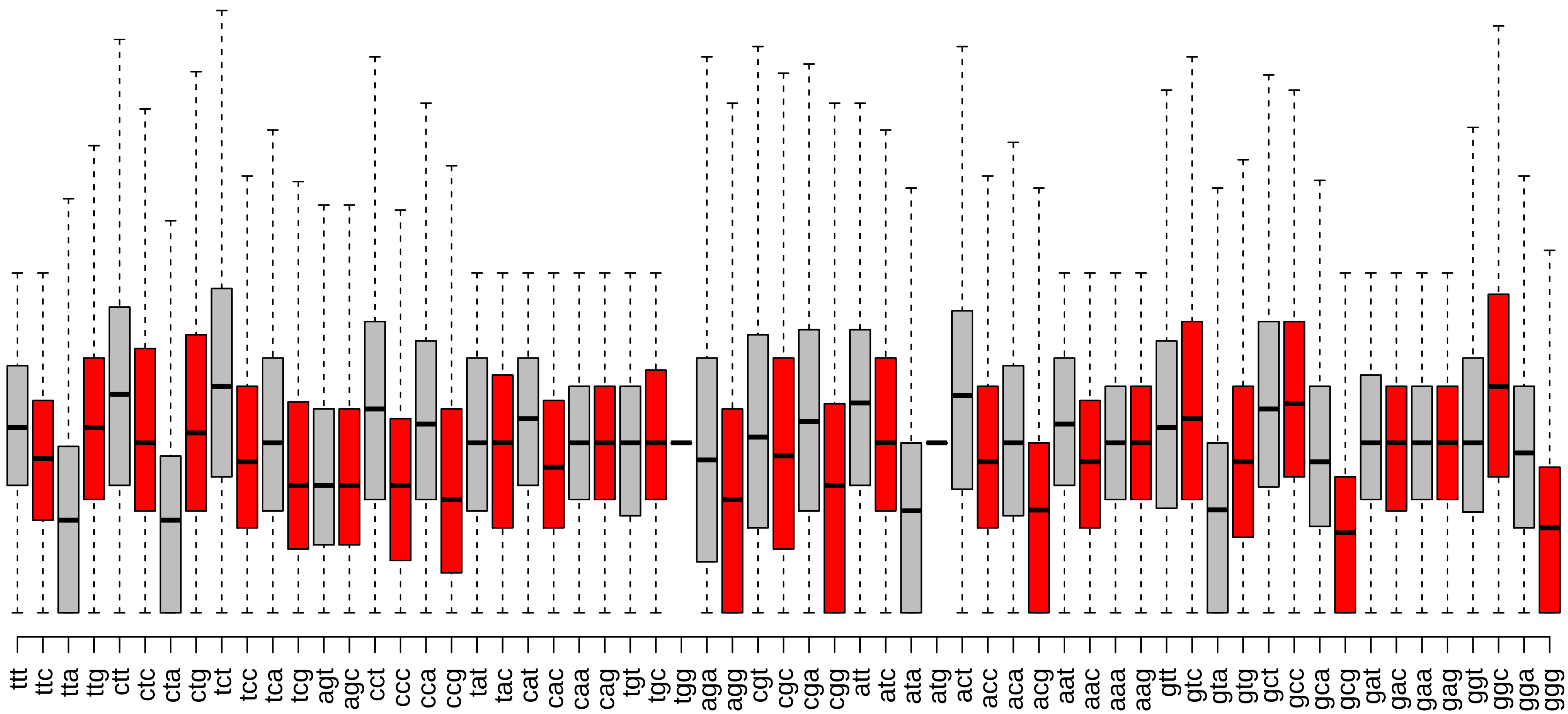

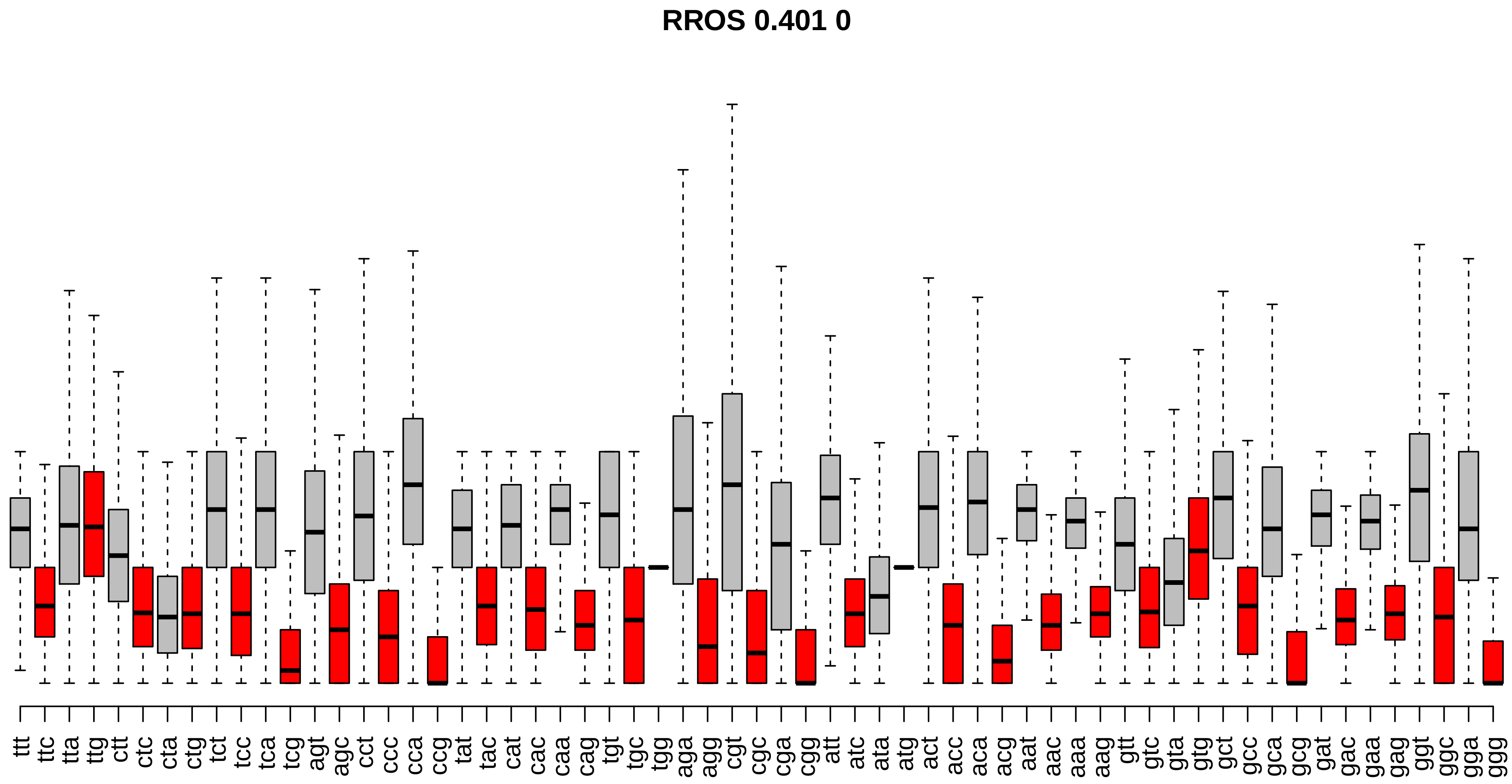
RROS 0.401 0
ttt ttc tta ttg ctt ctc cta ctg tct tcc tca tcg agt agc cct ccc cca ccg tat tac cat cac caa cag tgt tgc tgg aga agg cgt cgc cga cgg att atc ata atg act acc aca acg aat aac aaa aag gtt gtc gta gtg gct gcc gca gcg gat gac gaa gag ggt ggc gga ggg

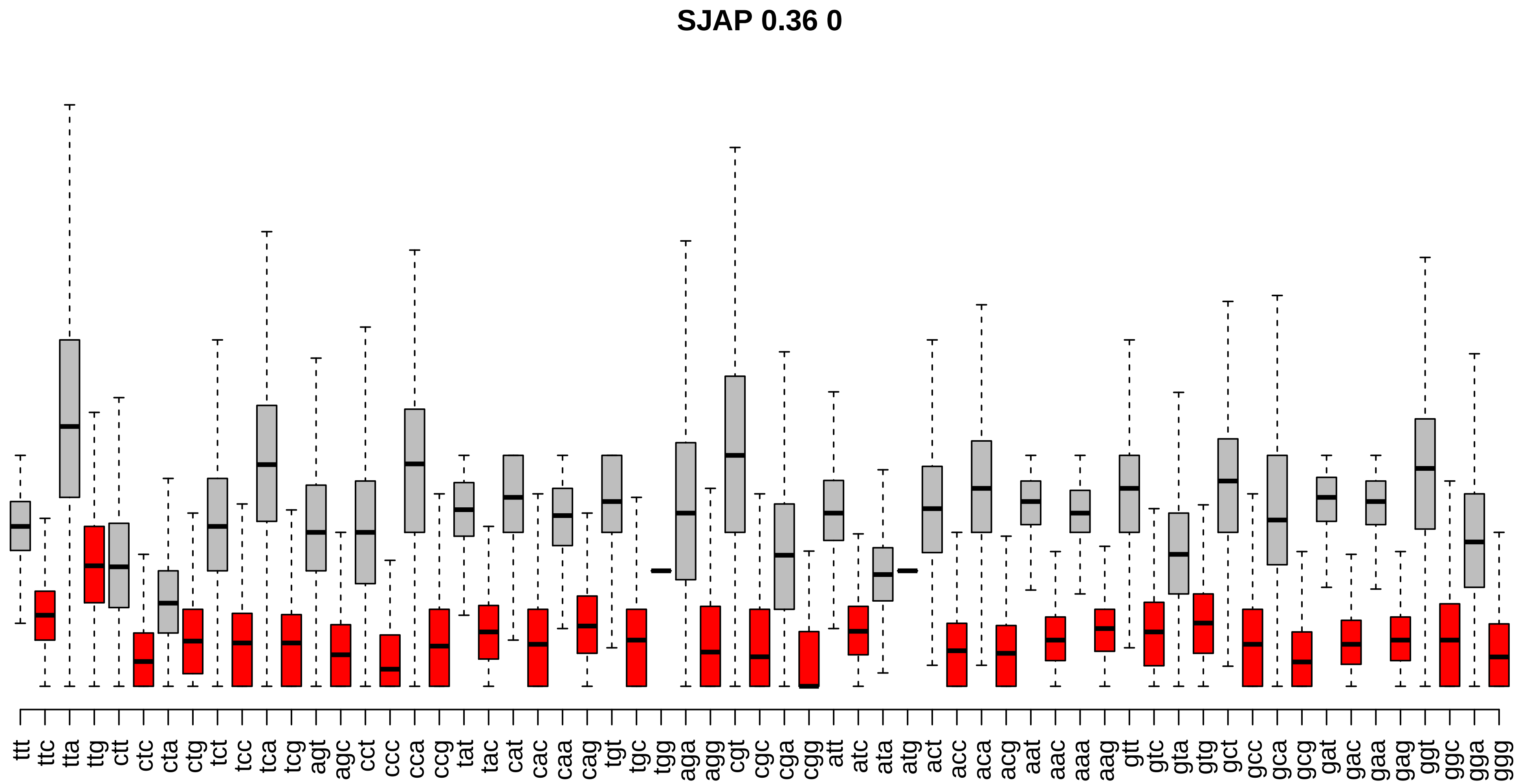
SJAP 0.36 0
ttt ttc tta ttg ctt ctc cta ctg tct tcc tca tcg agt agc cct ccc cca ccg tat tac cat cac caa cag tgt tgc tgg aga agg cgt cgc cga cgg att atc ata atg act acc aca acg aat aac aaa aag gtt gtc gta gtg gct gcc gca gcg gat gac gaa gag ggt ggc gga ggg

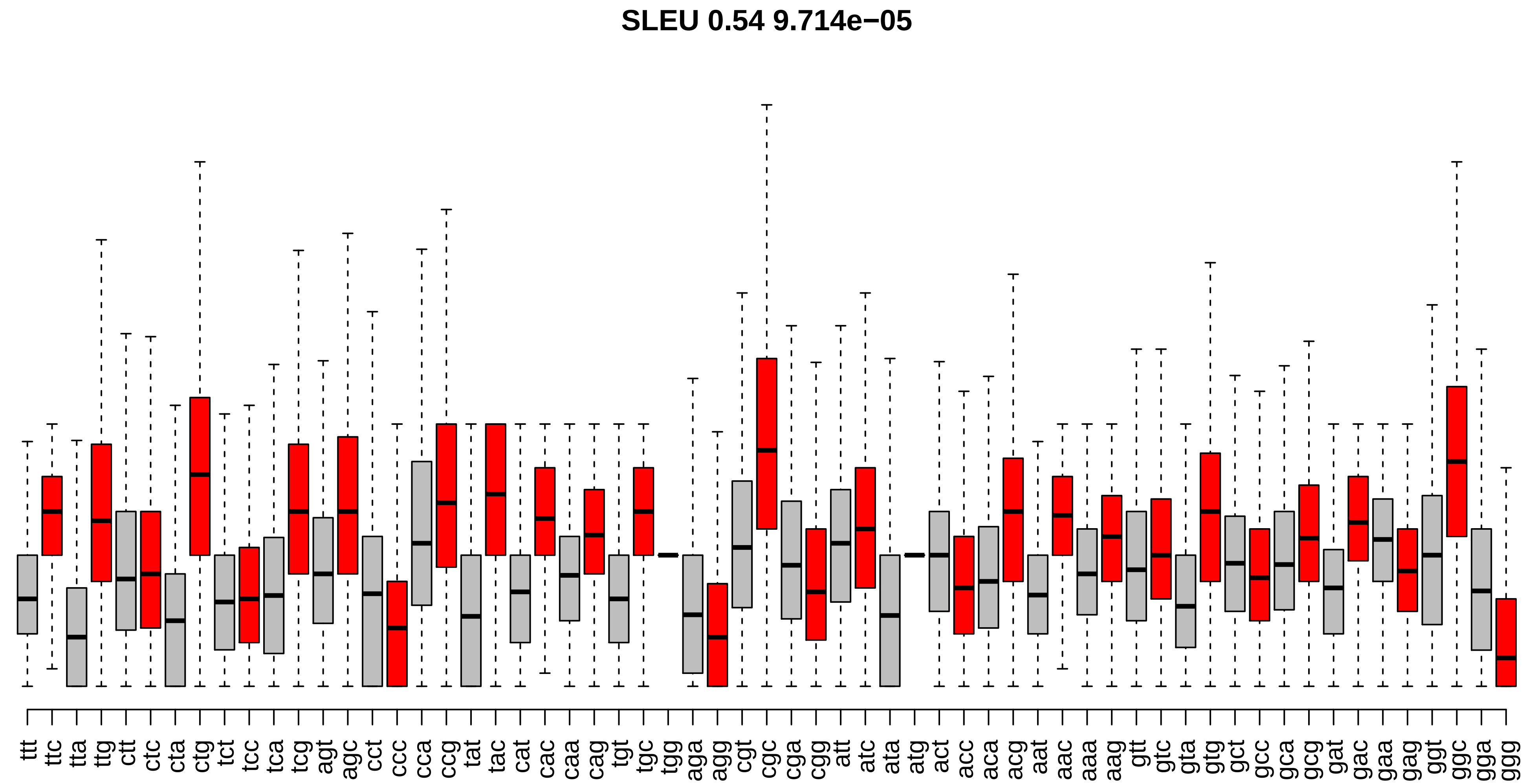
SLEU 0.54 9.714e−05
ttt ttc tta ttg ctt ctc cta ctg tct tcc tca tcg agt agc cct ccc cca ccg tat tac cat cac caa cag tgt tgc tgg aga agg cgt cgc cga cgg att atc ata atg act acc aca acg aat aac aaa aag gtt gtc gta gtg gct gcc gca gcg gat gac gaa gag ggt ggc gga ggg

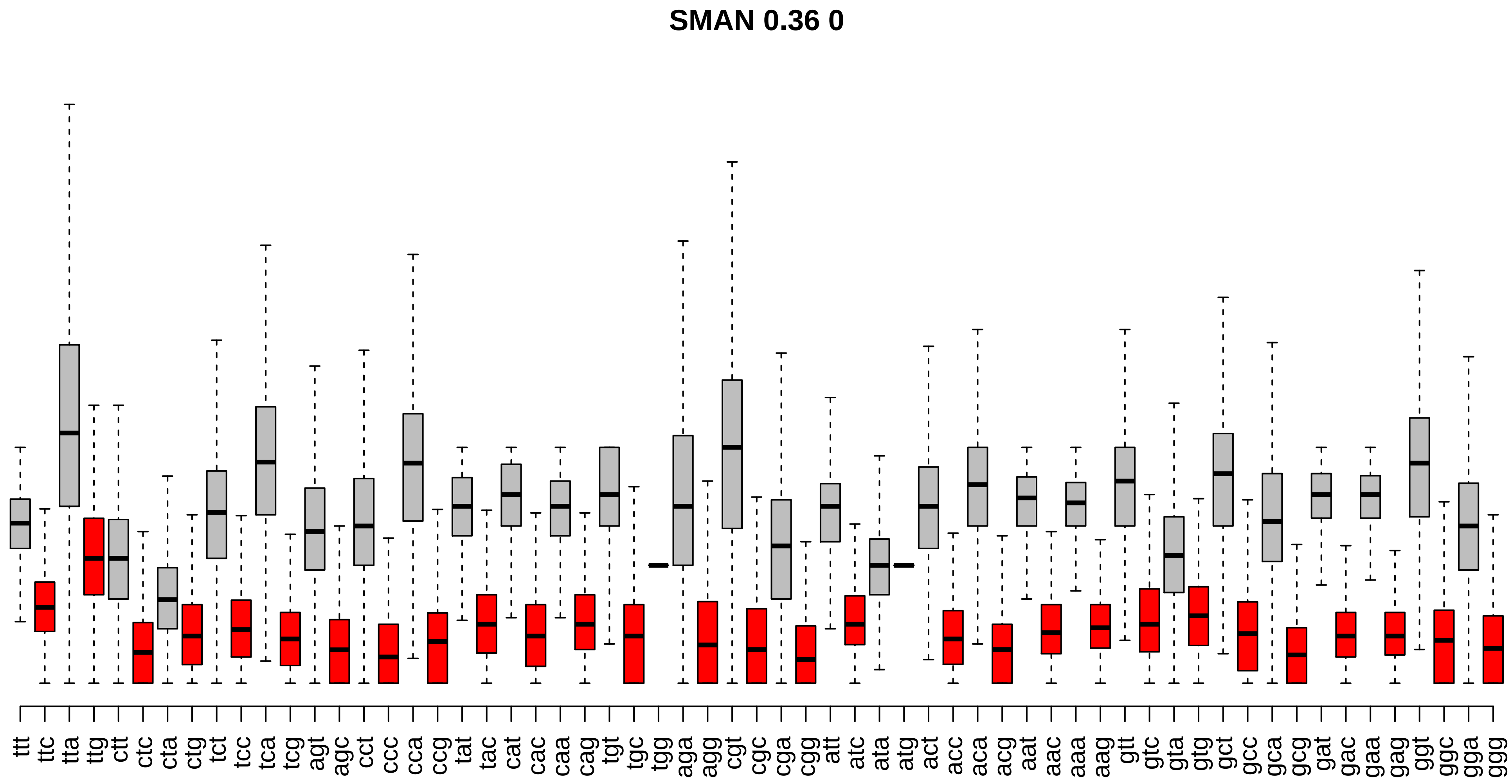
SMAN 0.36 0
ttt
ttc
tta
ttg
ctt
ctc
cta
ctg
tct
tcc
tca
tcg
agt
agc
cct
ccc
cca
ccg
tat
tac
cat
cac
caa
cag
tgt
tgc
tgg
aga
agg
cgt
cgc
cga
cgg
att
atc
ata
atg
act
acc
aca
acg
aat
aac
aaa
aag
gtt
gtc
gta
gtg
gct
gcc
gca
gcg
gat
gac
gaa
gag
ggt
ggc
gga
ggg

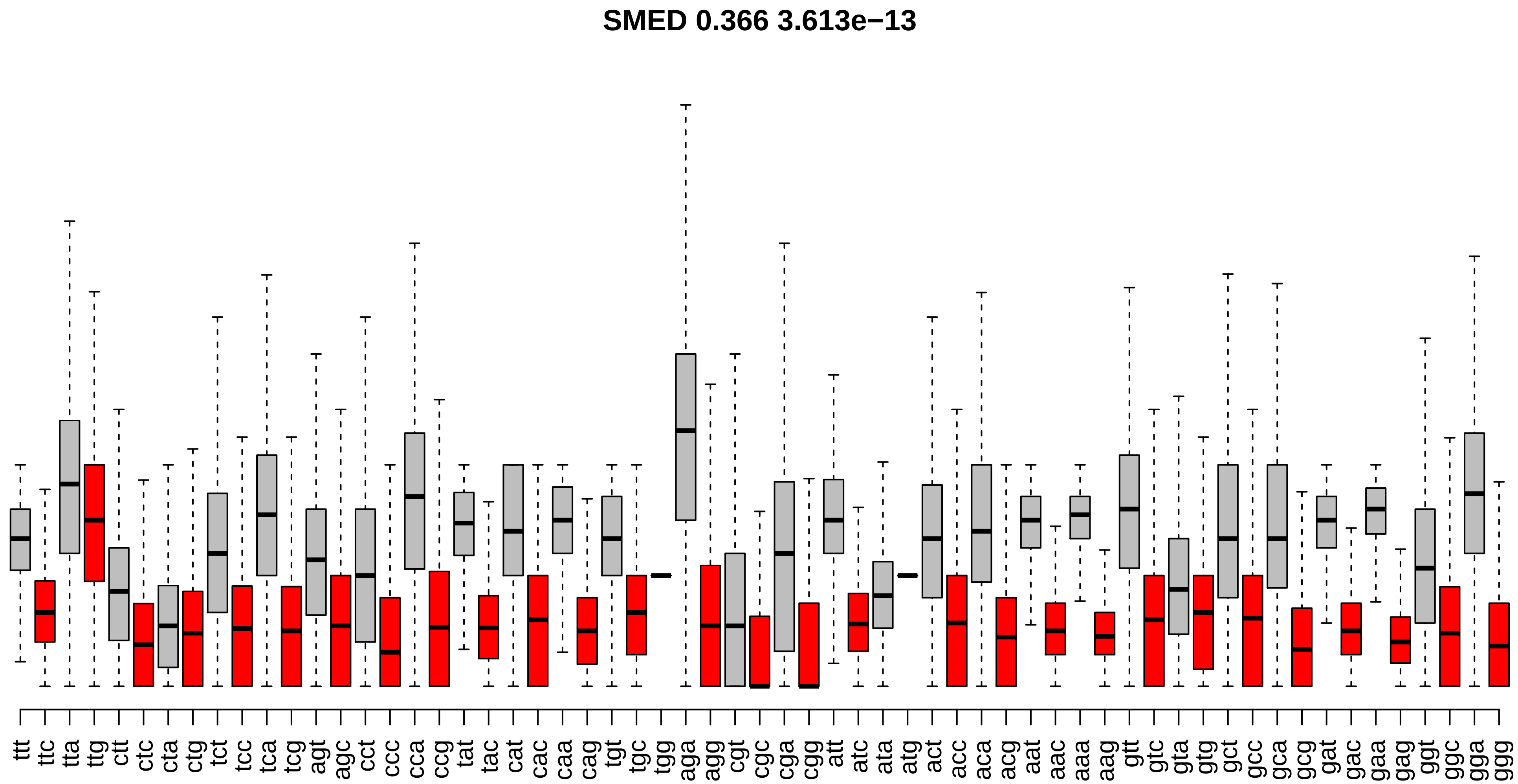
SMED 0.366 3.613e−13
ttt ttc tta ttg ctt ctc cta ctg tct tcc tca tcg agt agc cct ccc cca ccg tat tac cat cac caa cag tgt tgc tgg aga agg cgt cgc cga cgg att atc ata atg act acc aca acg aat aac aaa aag gtt gtc gta gtg gct gcc gca gcg gat gac gaa gag ggt ggc gga ggg

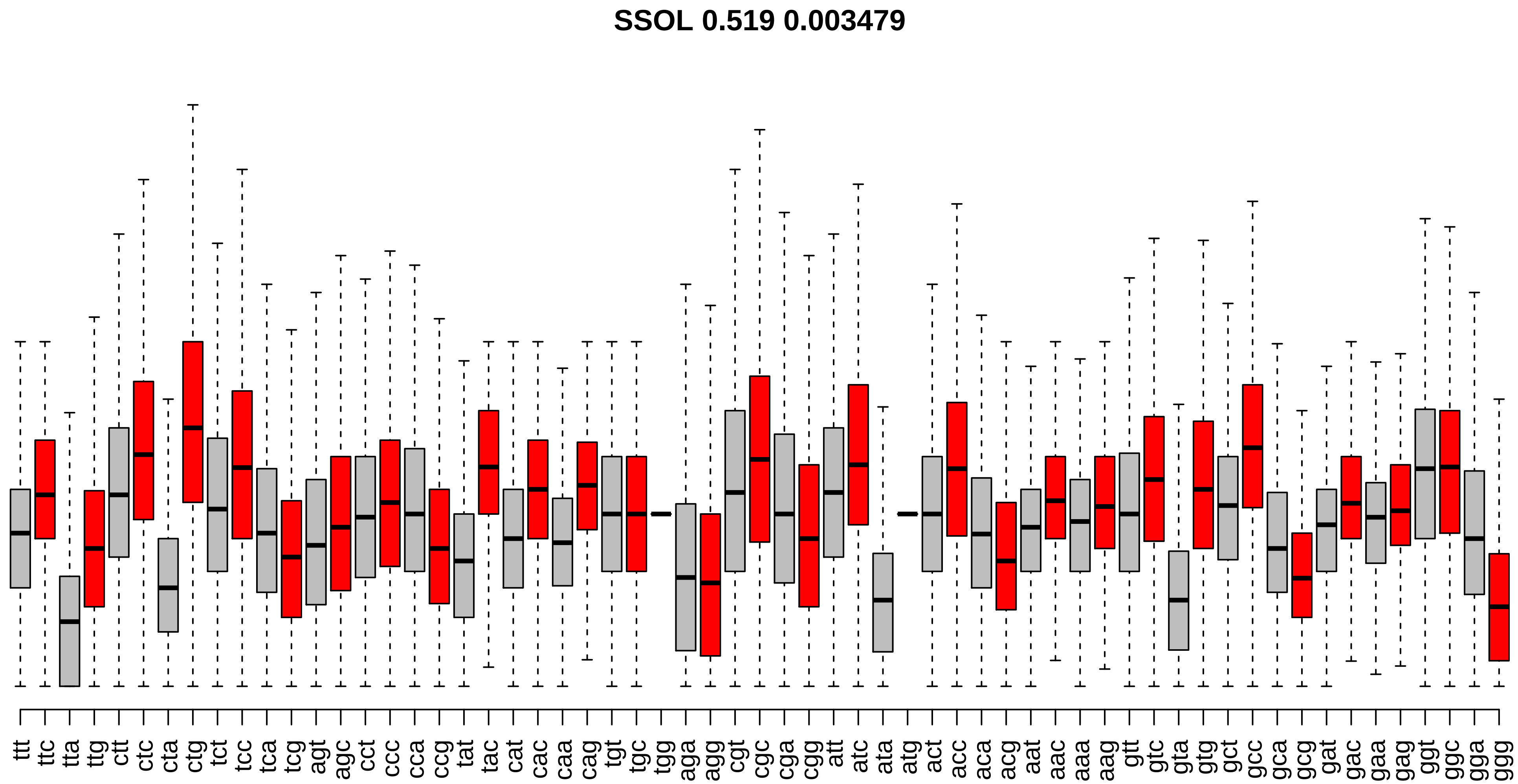
SSOL 0.519 0.003479
ttt
ttc
tta
ttg
ctt
ctc
cta
ctg
tct
tcc
tca
tcg
agt
agc
cct
ccc
cca
ccg
tat
tac
cat
cac
caa
cag
tgt
tgc
tgg
aga
agg
cgt
cgc
cga
cgg
att
atc
ata
atg
act
acc
aca
acg
aat
aac
aaa
aag
gtt
gtc
gta
gtg
gct
gcc
gca
gcg
gat
gac
gaa
gag
ggt
ggc
gga
ggg

TREG 0.372 0

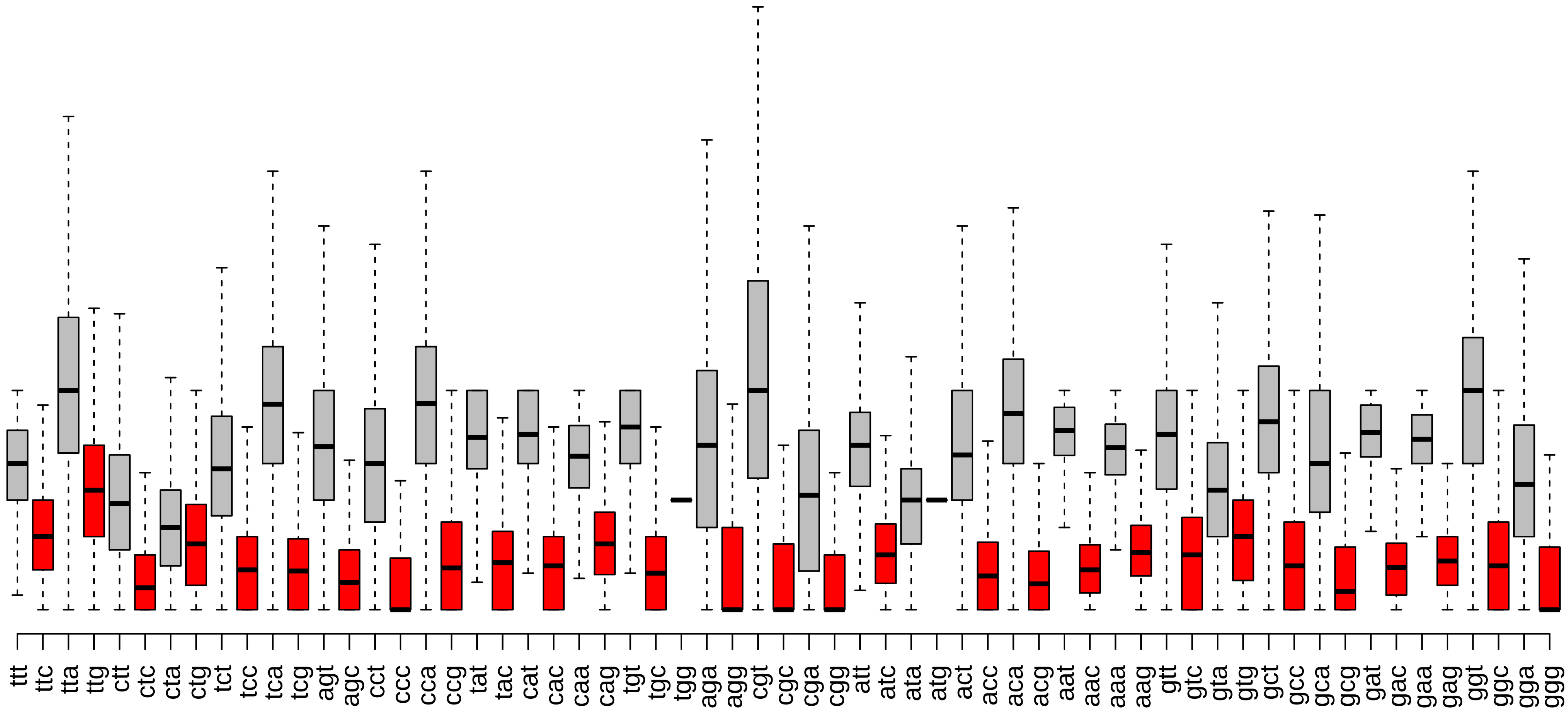

ttt
ttc
tta
ttg
ctt
ctc
cta
ctg
tct
tcc
tca
tcg
agt
agc
cct
ccc
cca
ccg
tat
tac
cat
cac
caa
cag
tgt
tgc
tgg
aga
agg
cgt
cgc
cga
cgg
att
atc
ata
atg
act
acc
aca
acg
aat
aac
aaa
aag
gtt
gtc
gta
gtg
gct
gcc
gca
gcg
gat
gac
gaa
gag
ggt
ggc
gga
ggg

SFig3. GC3-ENC plots of the 22 species (for details see Materials & Methods).

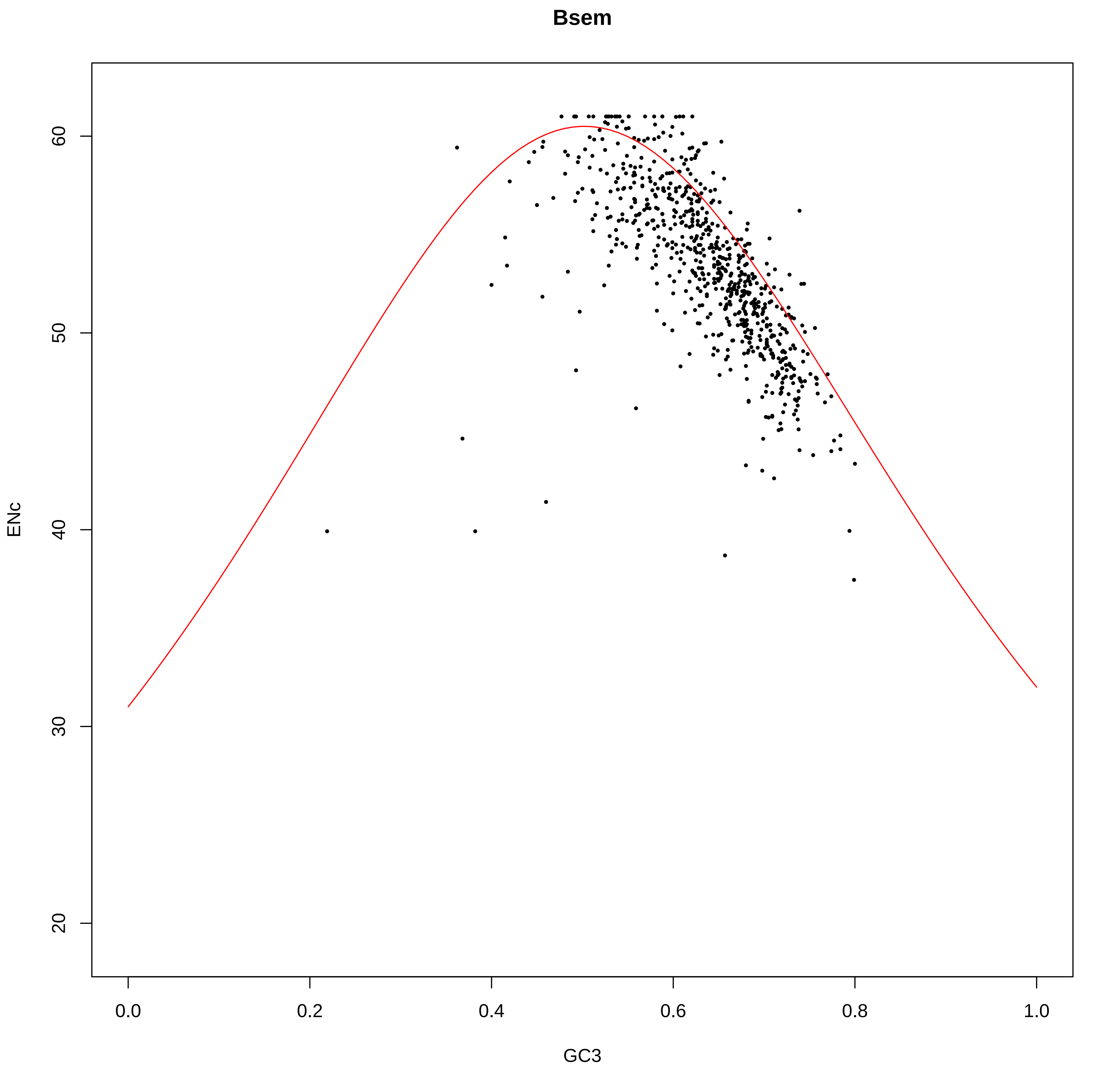

Bsem
ENc
GC3
20
30
40
50
60
0.0
0.2
0.4
0.6
0.8
1.0

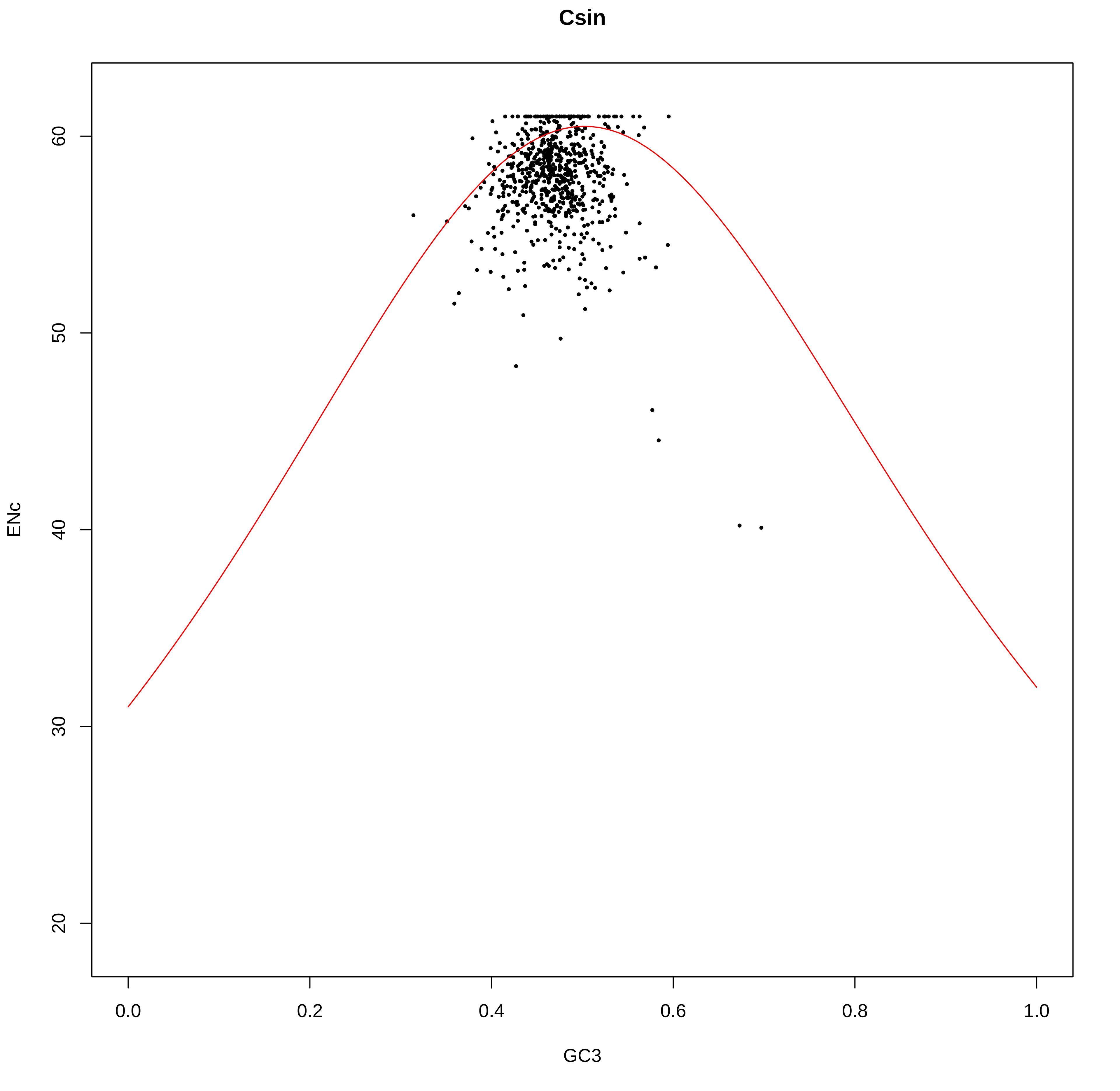

Csin
ENc
GC3
60
50
40
30
20
0.0
0.2
0.4
0.6
0.8
1.0

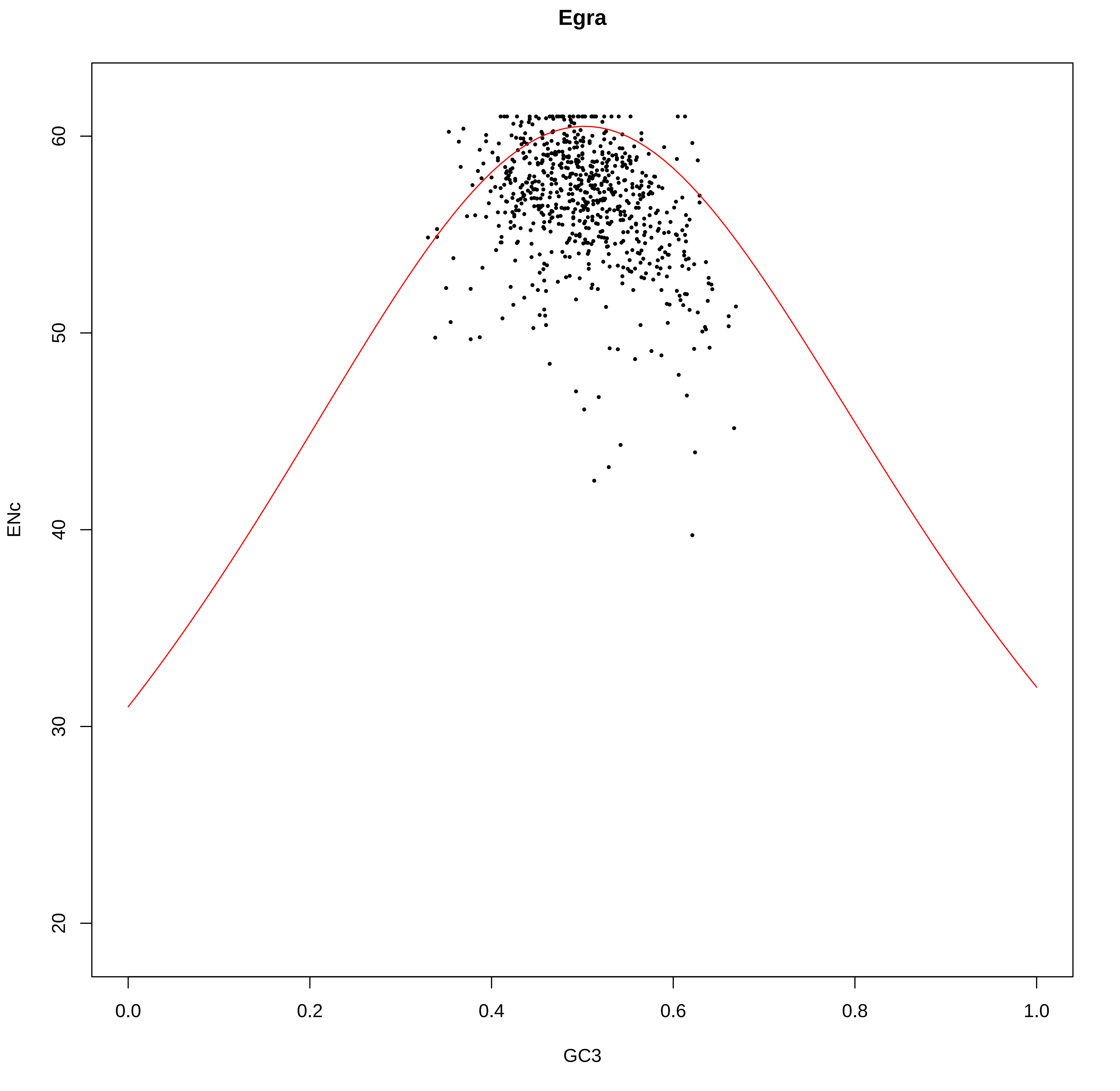

Egra
ENc
GC3
20
30
40
50
60
0.0
0.2
0.4
0.6
0.8
1.0

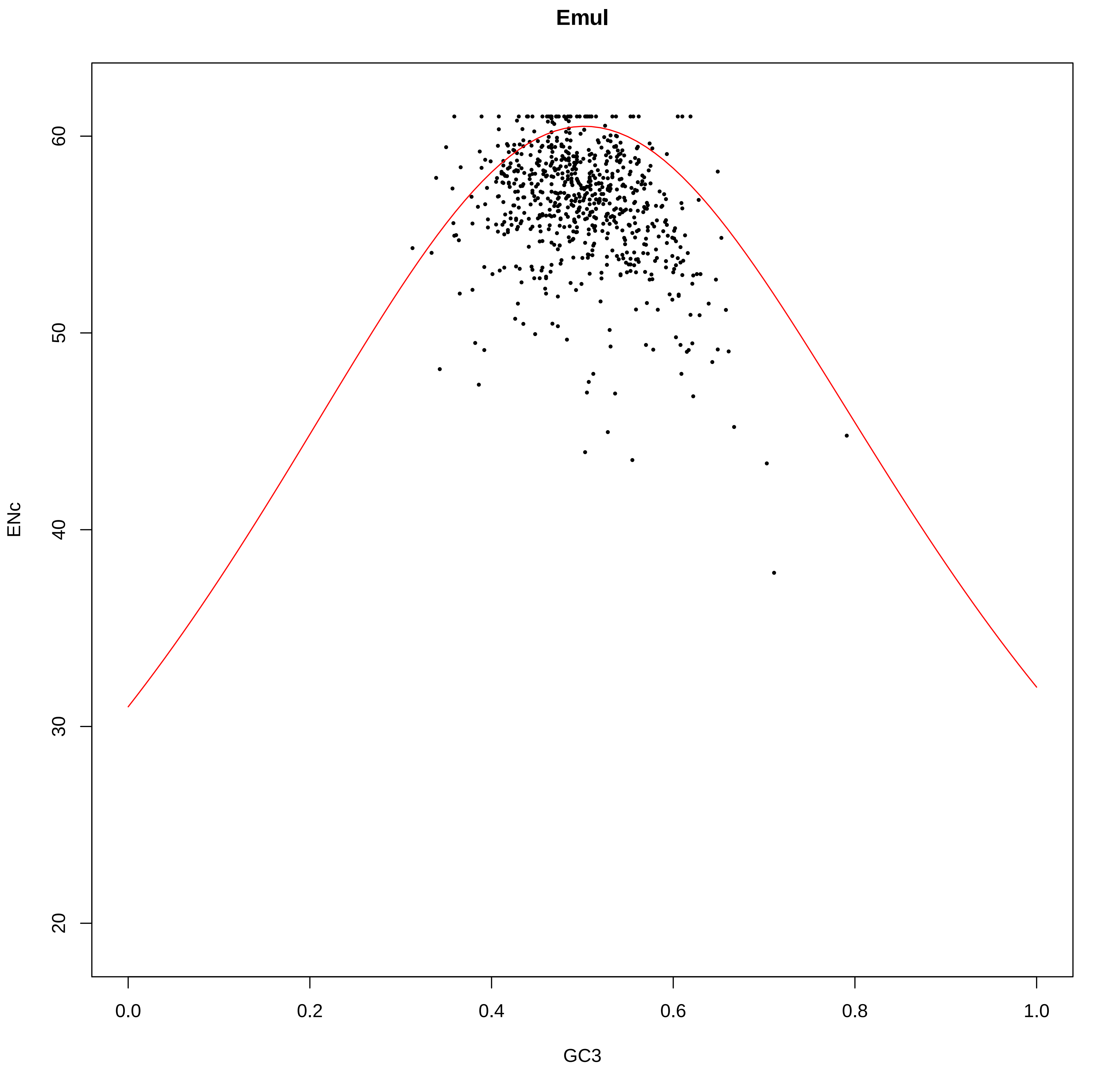

Emul
ENc
GC3
60
50
40
30
20
0.0
0.2
0.4
0.6
0.8
1.0

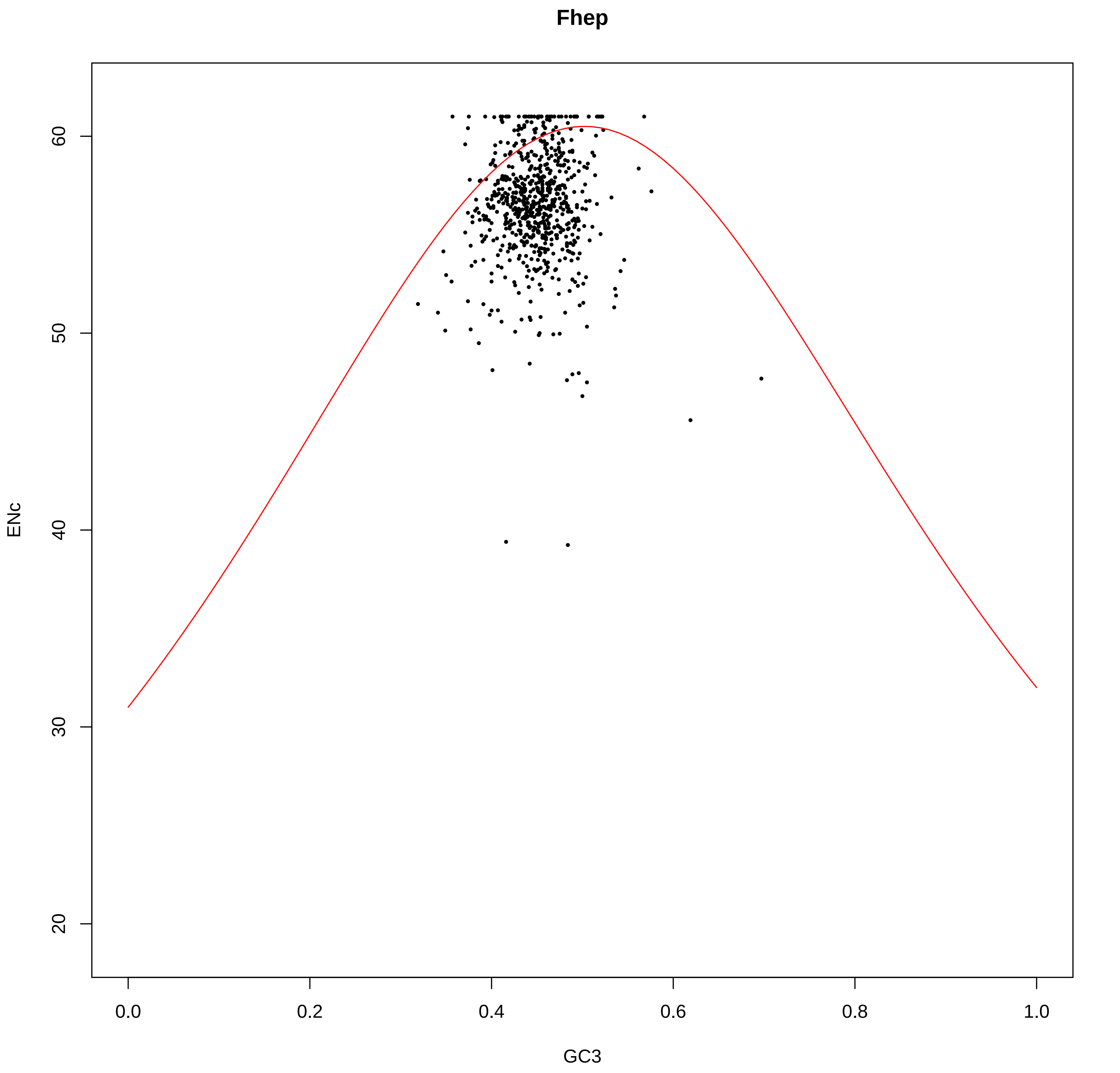

Fhep
ENc
GC3
20
30
40
50
60
0.0
0.2
0.4
0.6
0.8
1.0

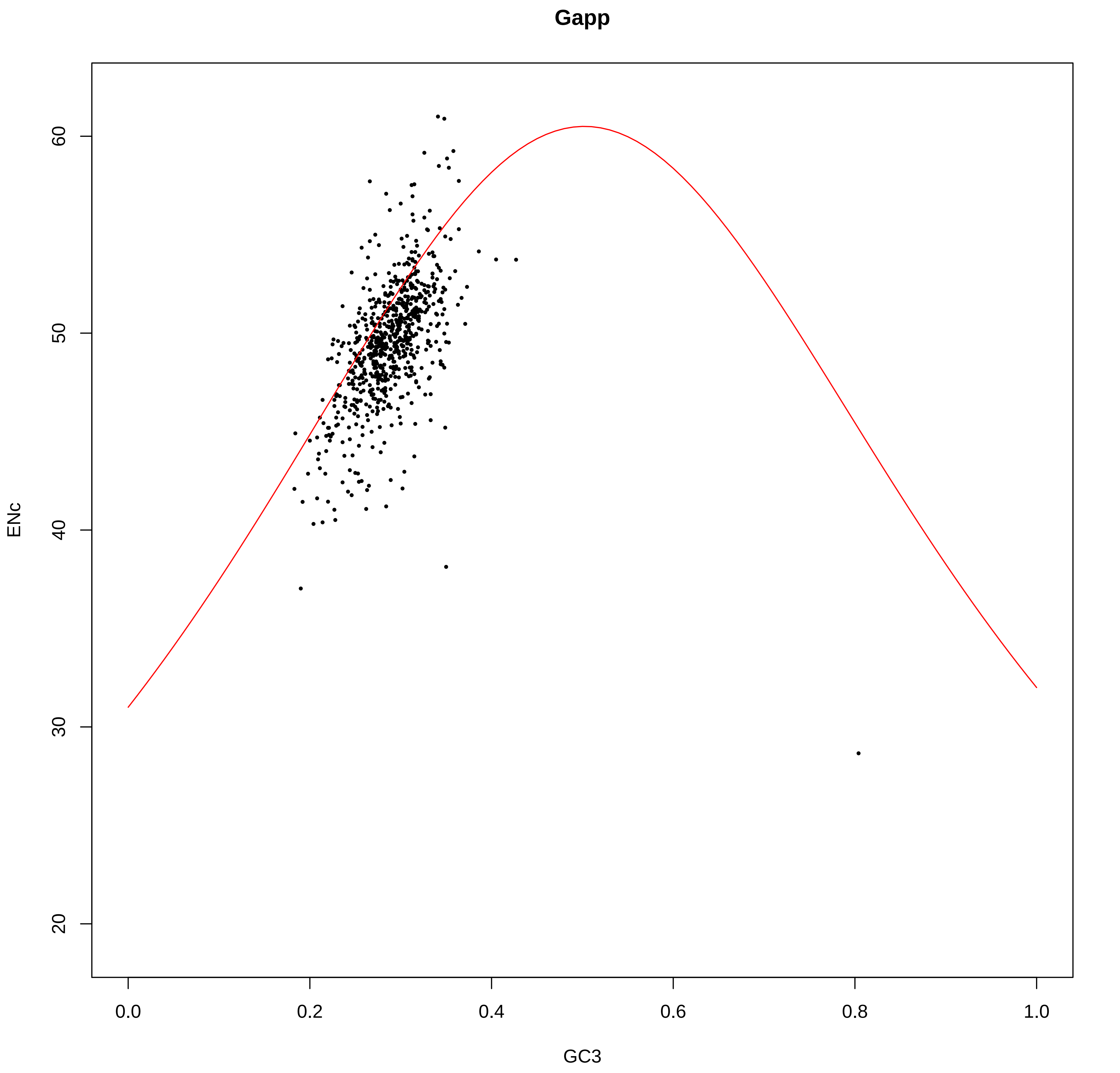

Gapp
ENc
GC3
60
50
40
30
20
0.0
0.2
0.4
0.6
0.8
1.0

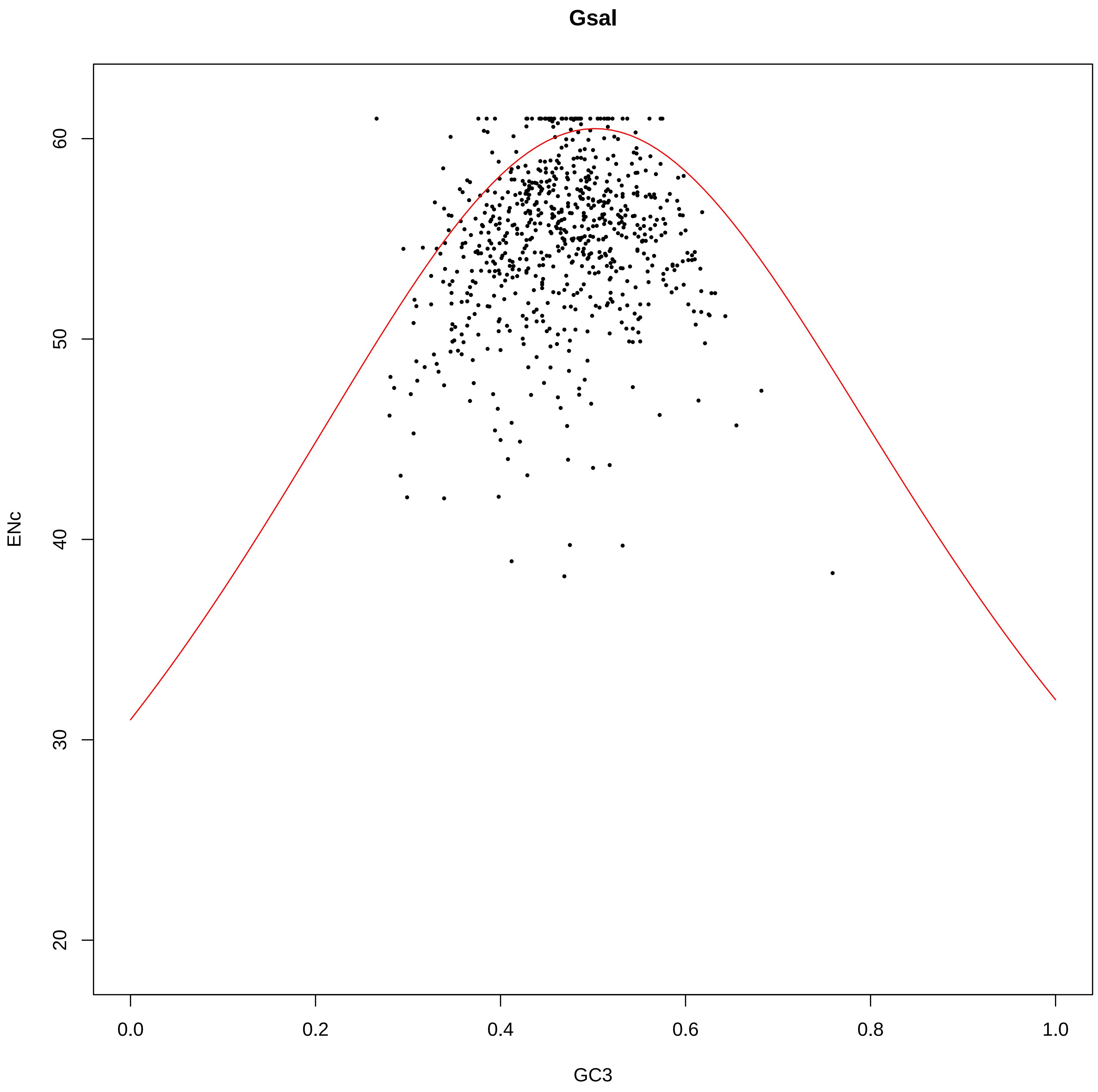
Gsal
ENc
GC3
60
50
40
30
20
0.0
0.2
0.4
0.6
0.8
1.0

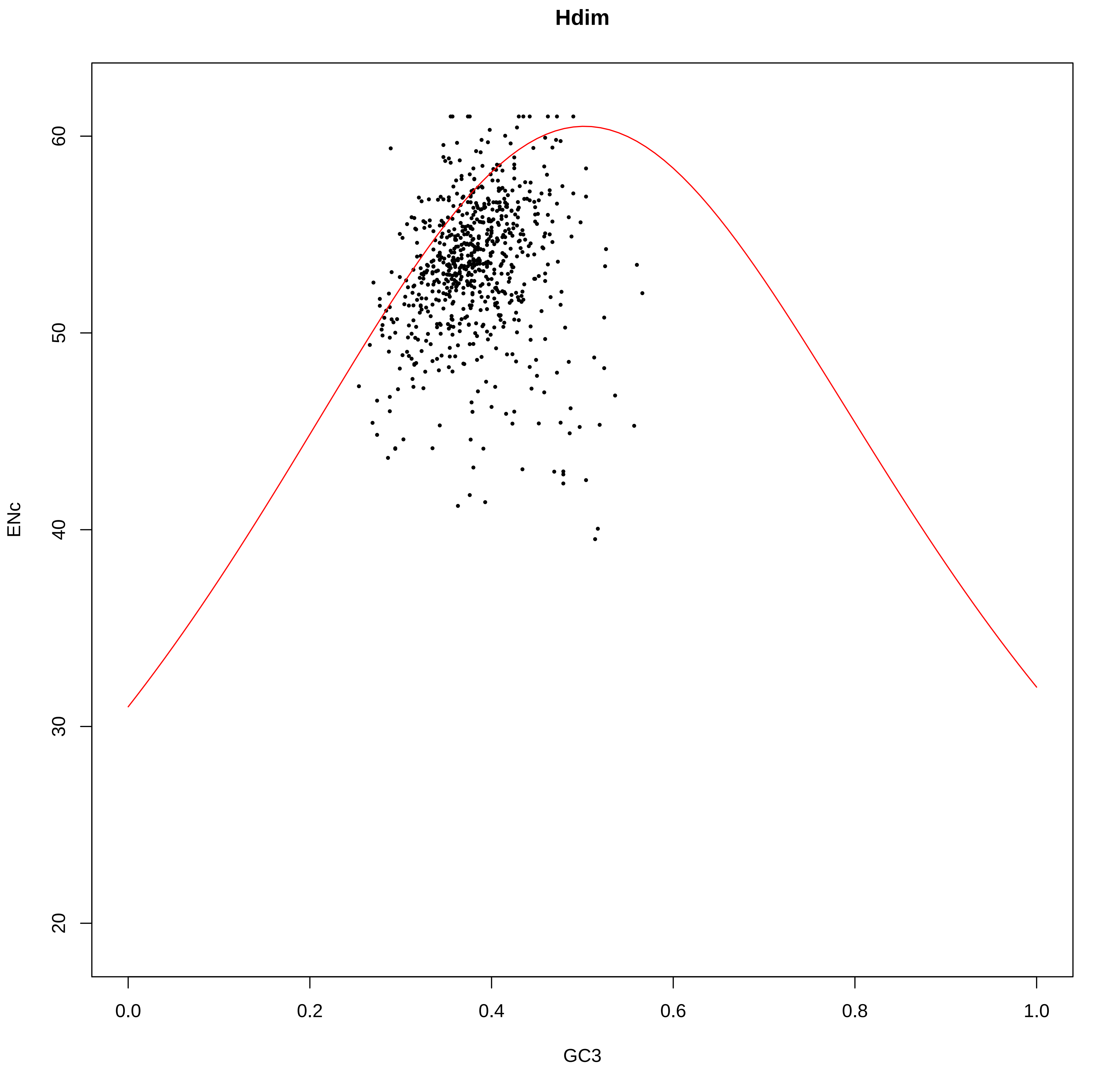
Hdim
ENc
GC3
20
30
40
50
60
0.0
0.2
0.4
0.6
0.8
1.0

Kamp

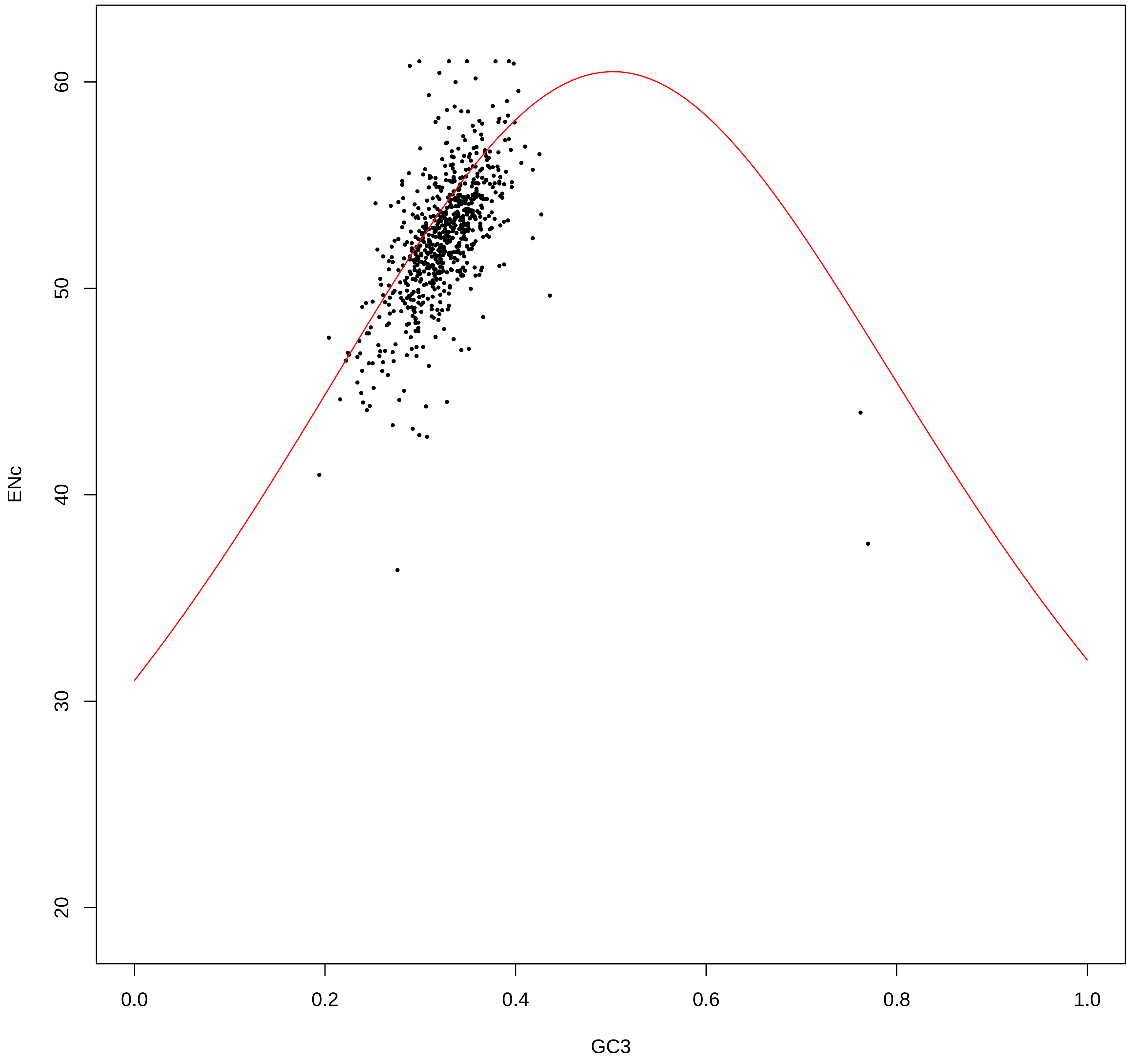

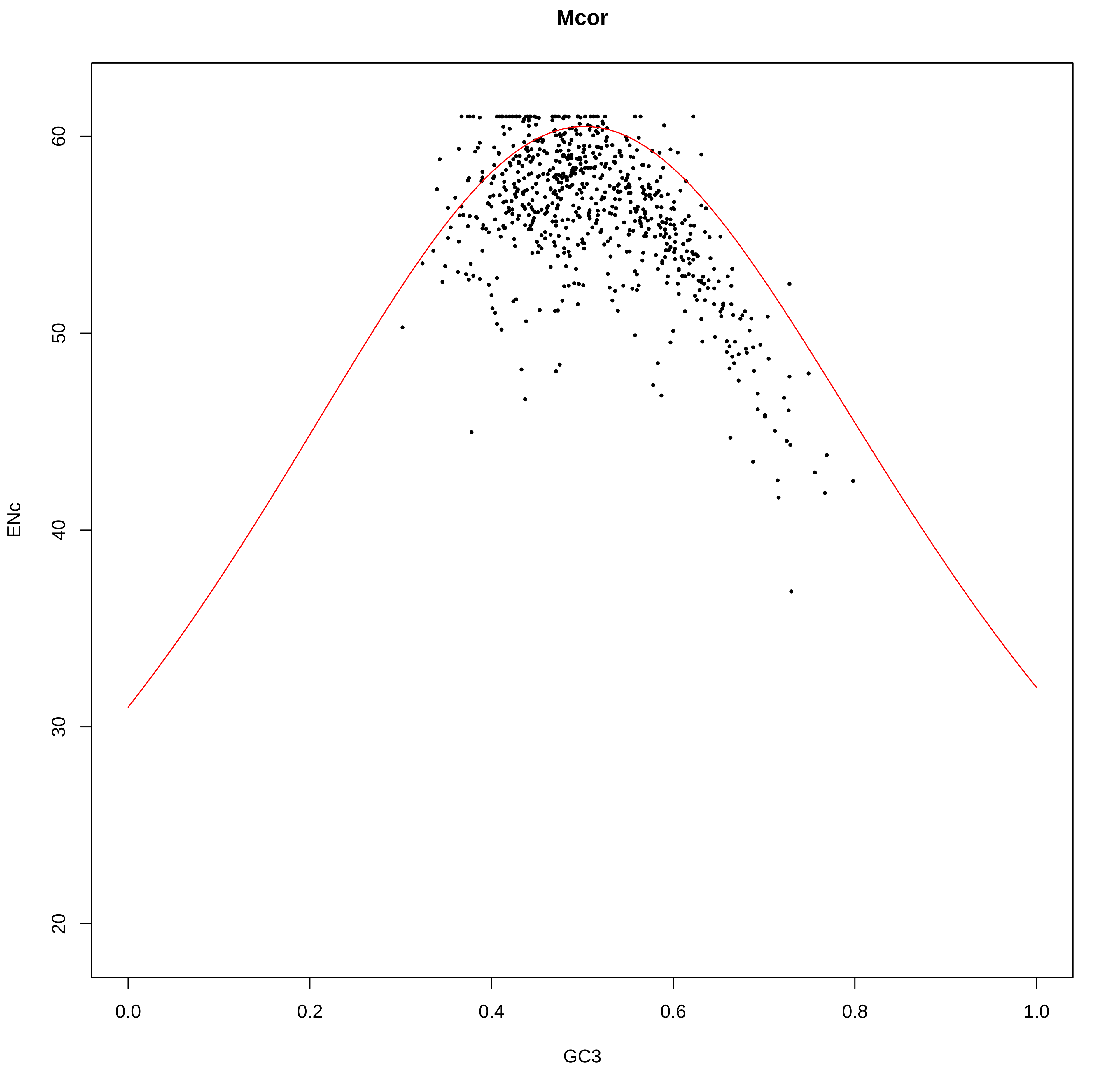
Mcor
ENc
GC3
60
50
40
30
20
0.0
0.2
0.4
0.6
0.8
1.0

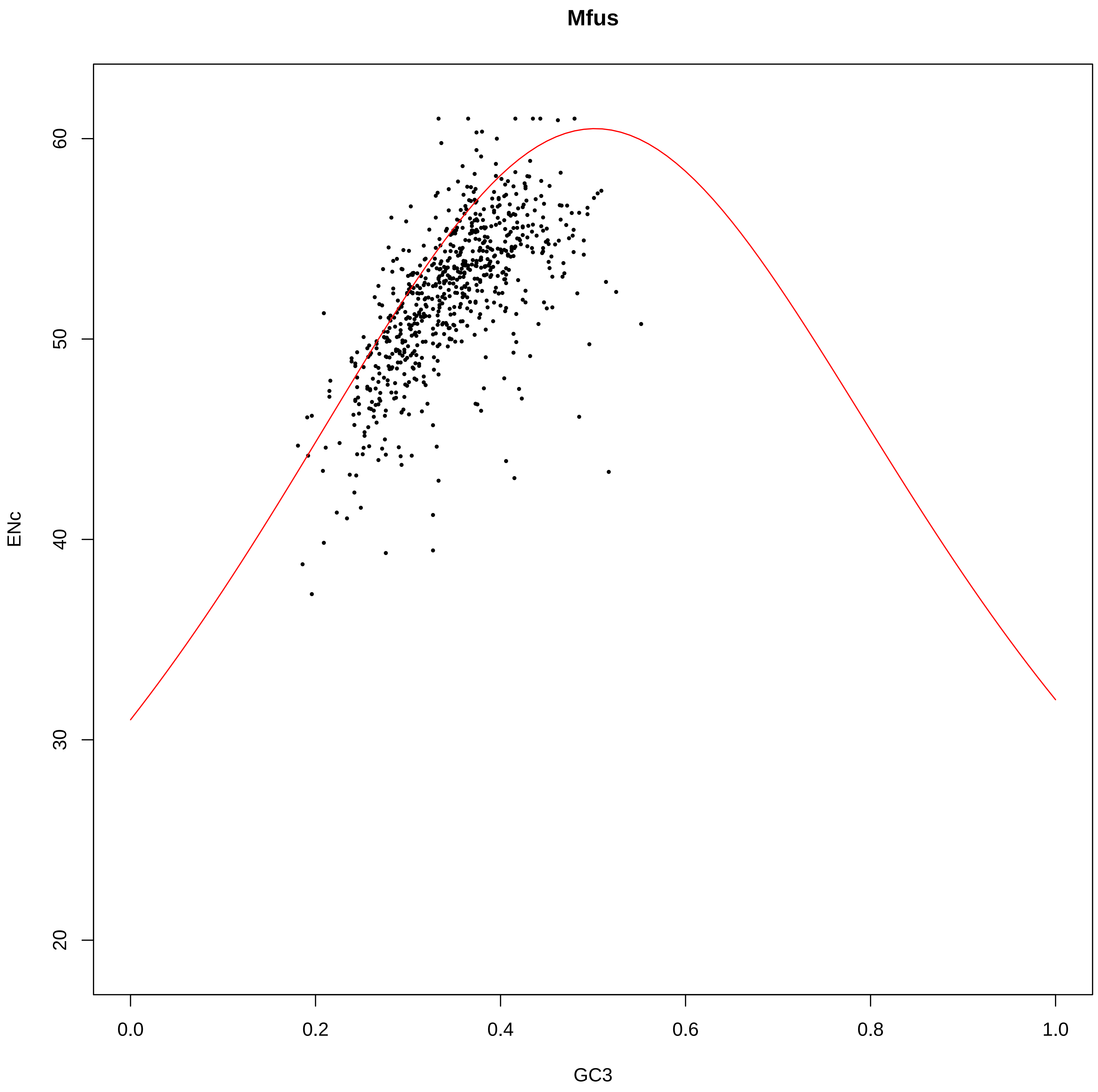

Mfus
ENc
GC3
20
30
40
50
60
0.0
0.2
0.4
0.6
0.8
1.0

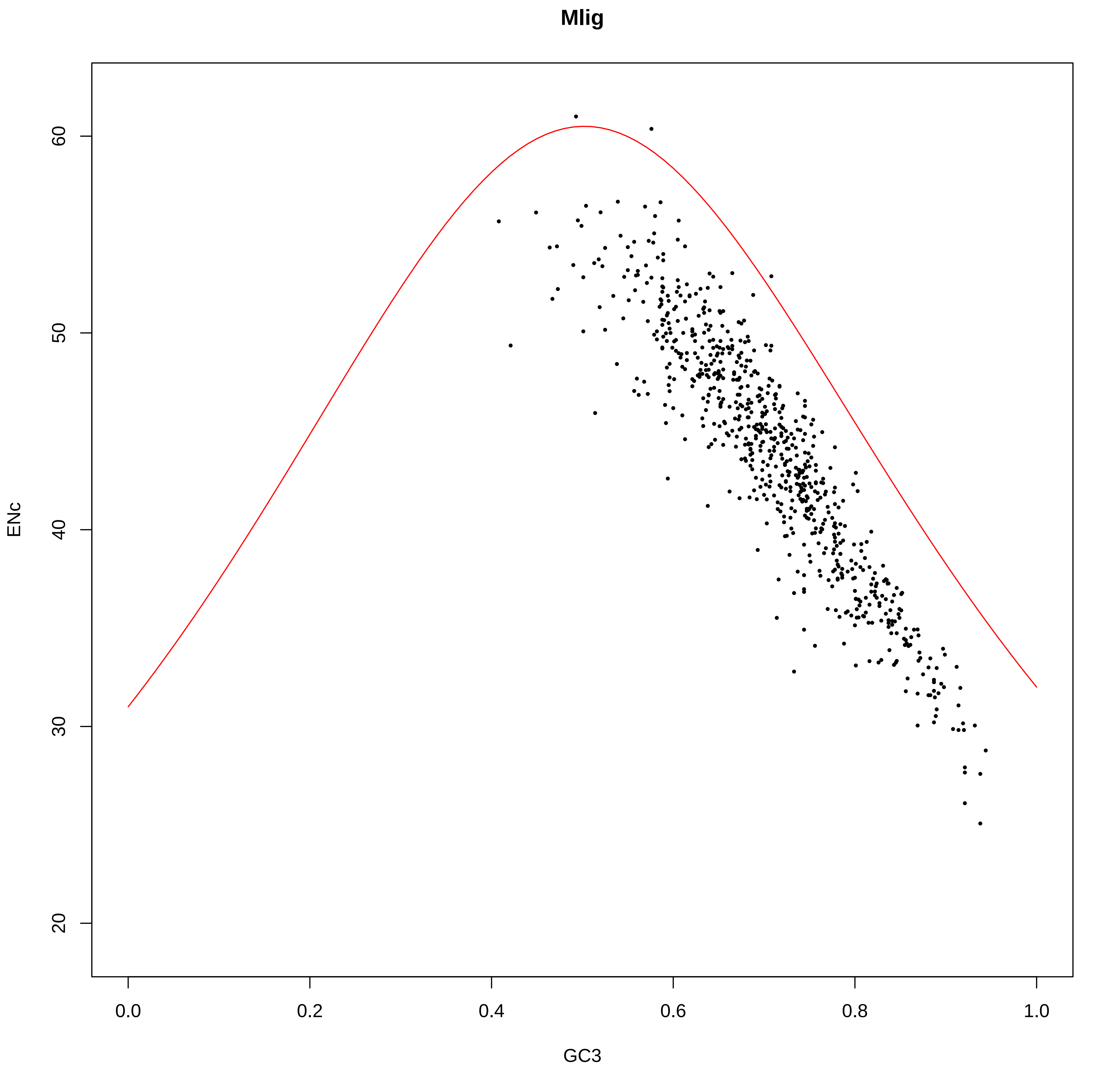

Mlig
ENc
GC3
60
50
40
30
20
0.0
0.2
0.4
0.6
0.8
1.0

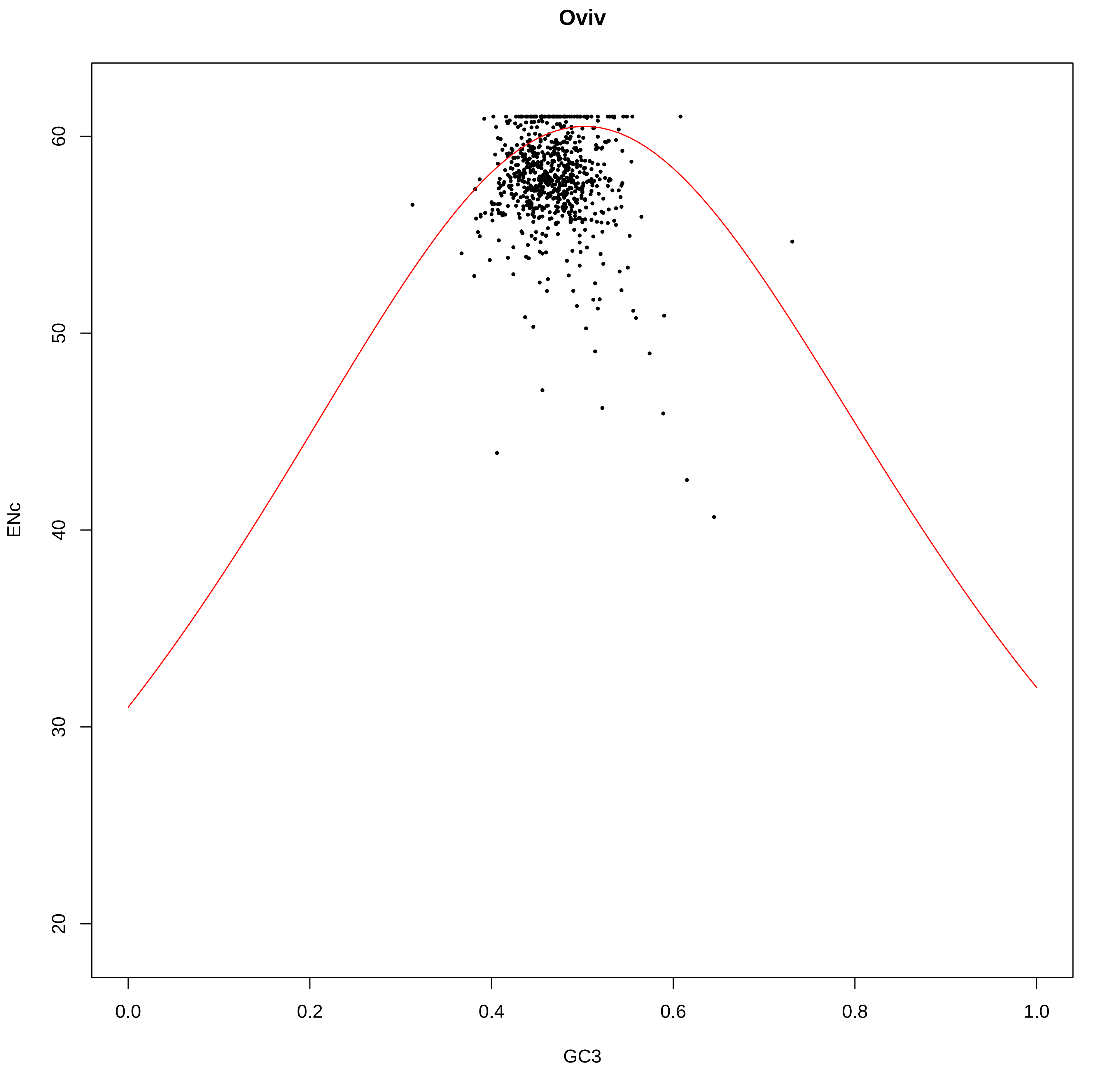

Oviv
60
50
40
30
20
ENc
0.0
0.2
0.4
0.6
0.8
1.0
GC3

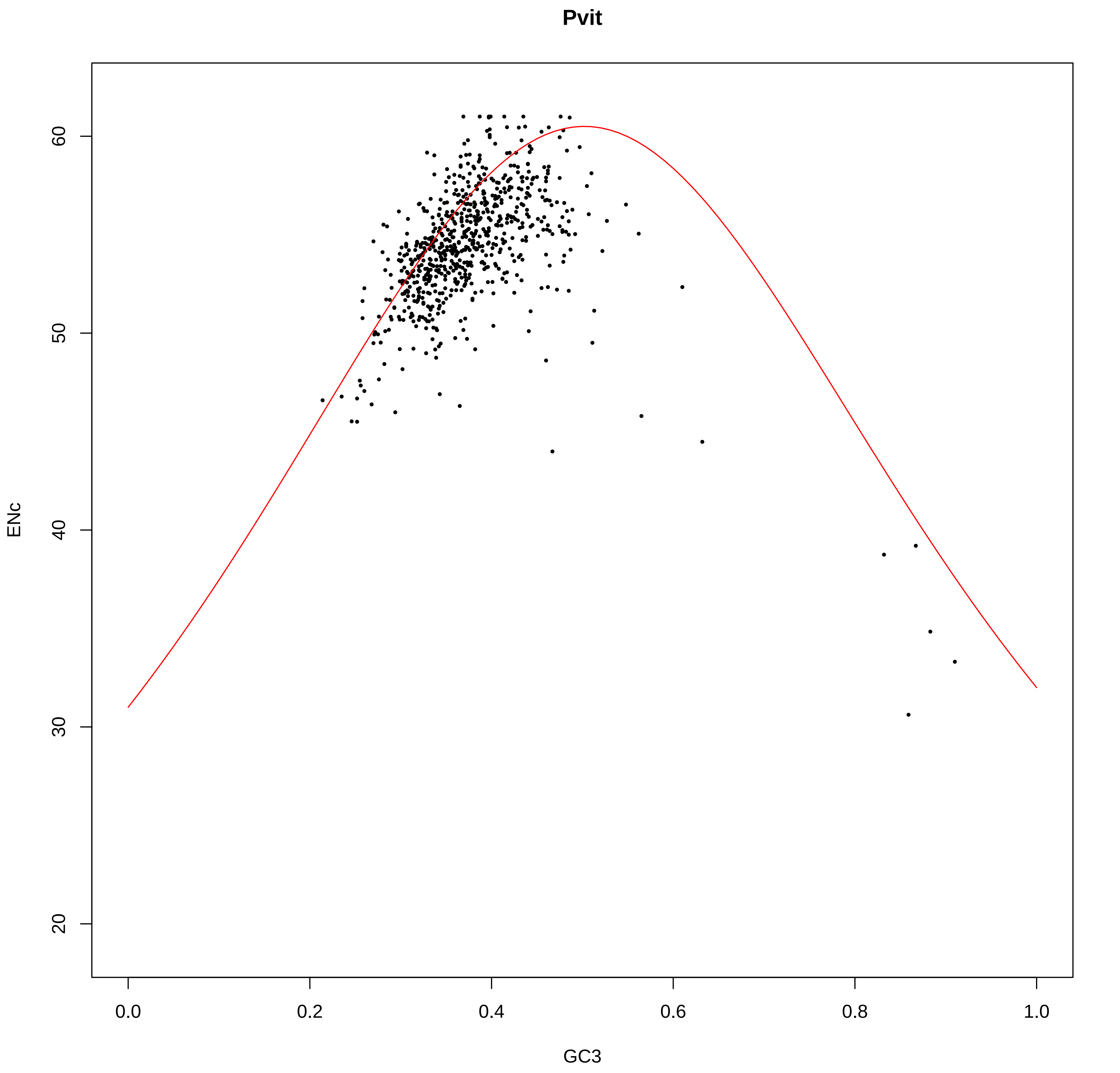

Pvit
ENc
GC3
60
50
40
30
20
0.0
0.2
0.4
0.6
0.8
1.0

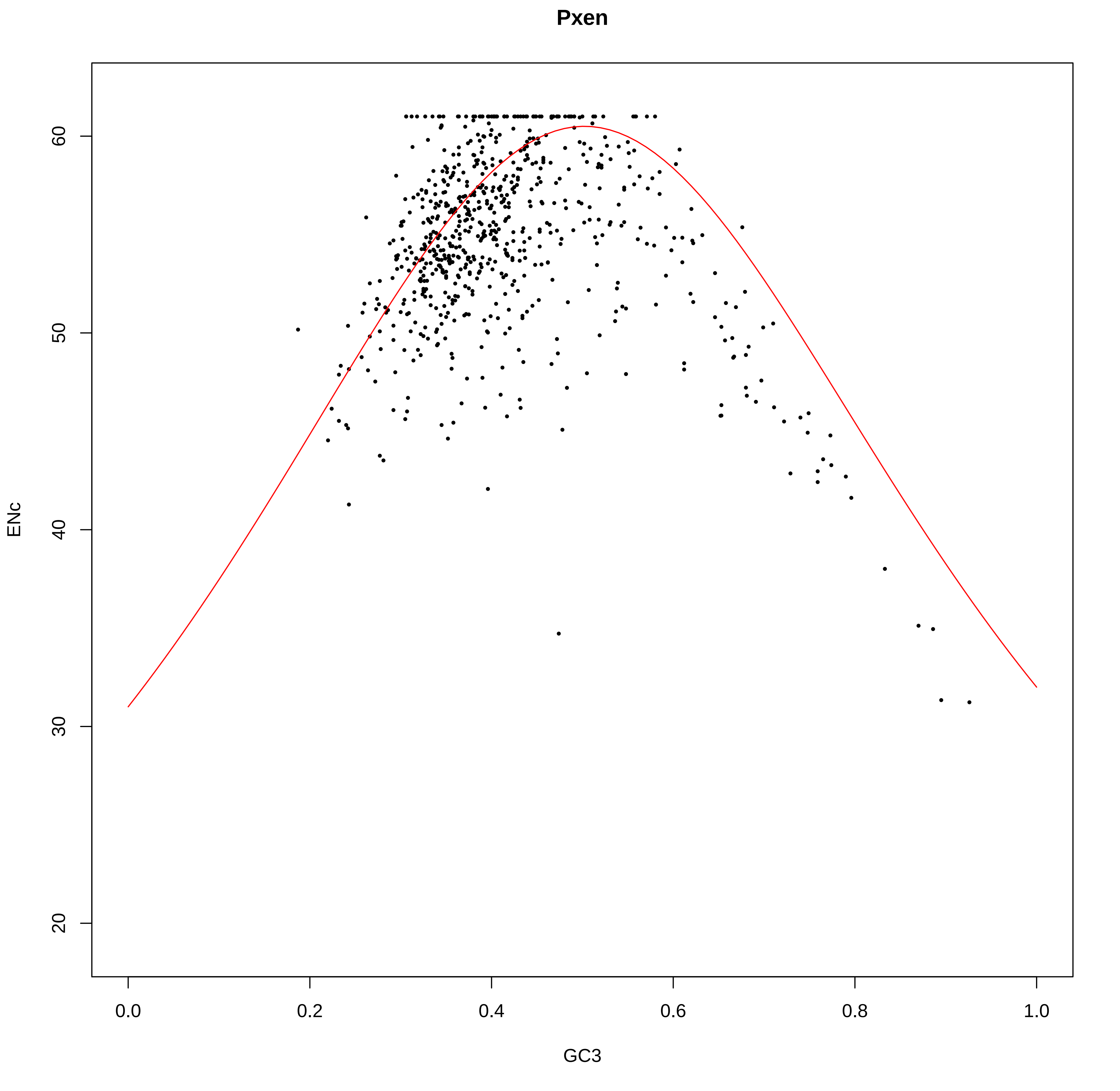
Pxen
ENc
GC3
20
30
40
50
60
0.0
0.2
0.4
0.6
0.8
1.0

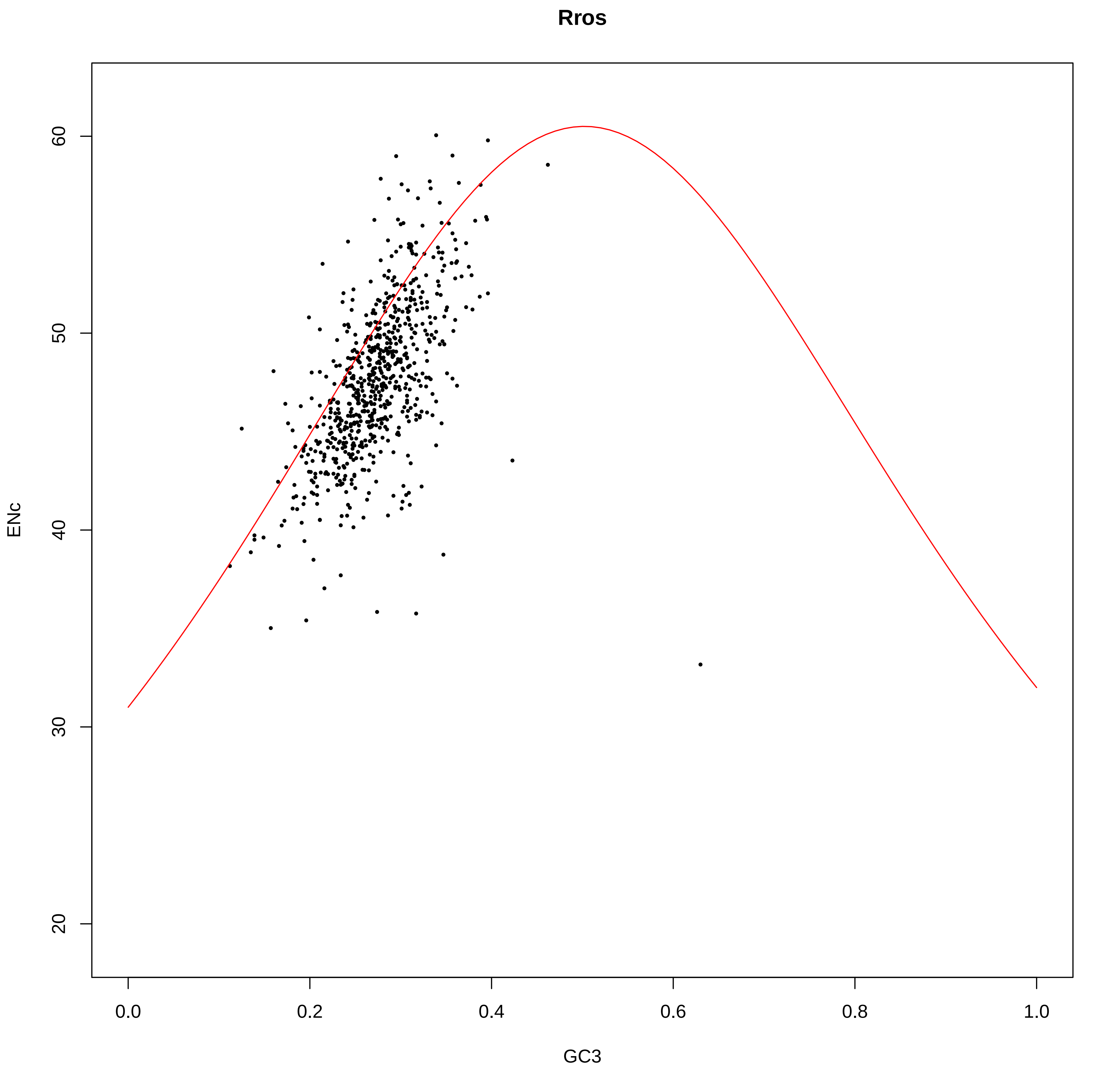
Rros
ENc
GC3
60
50
40
30
20
0.0
0.2
0.4
0.6
0.8
1.0

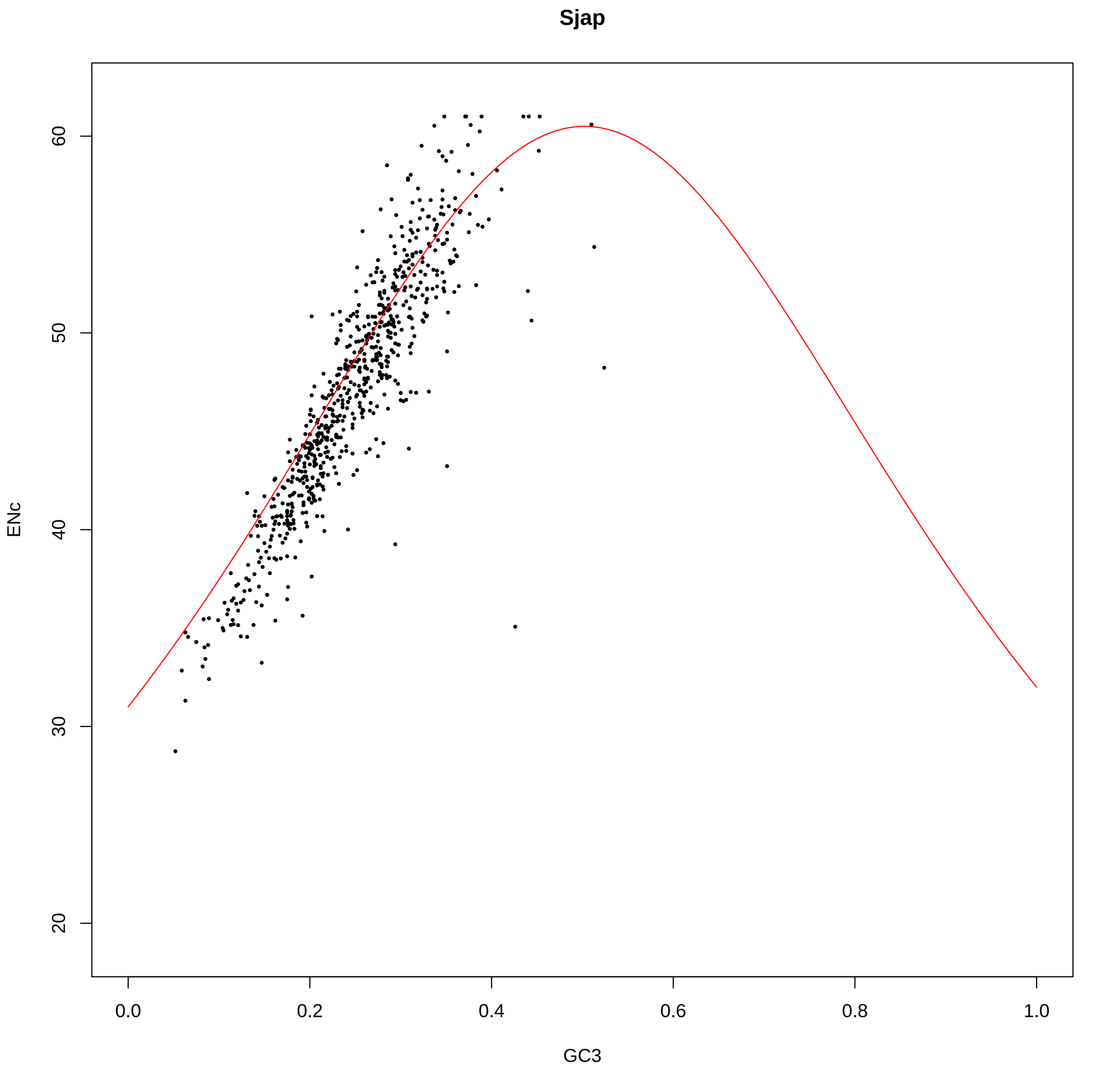

Sjap
ENc
GC3
20
30
40
50
60
0.0
0.2
0.4
0.6
0.8
1.0

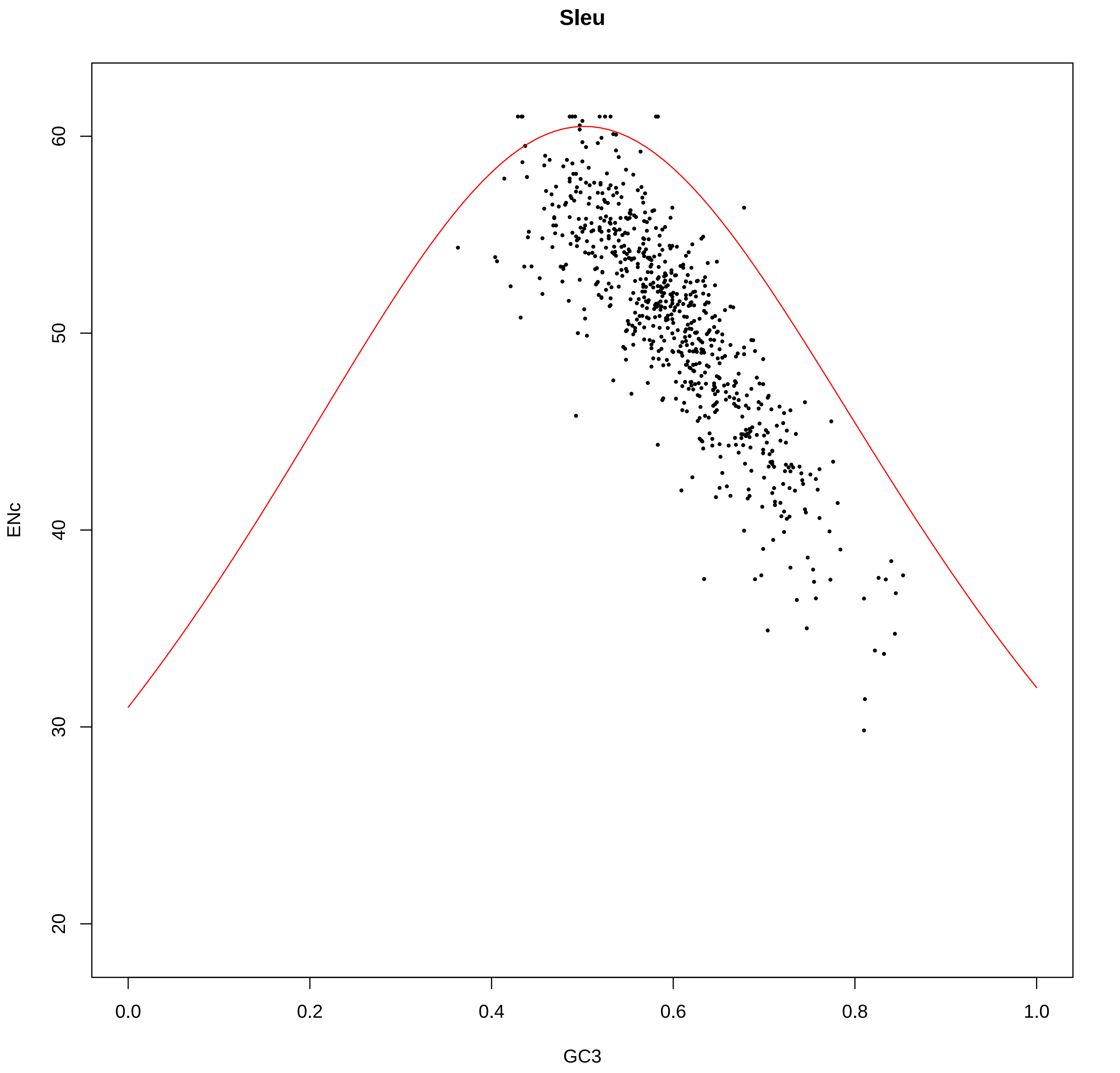
Sleu
ENc
GC3
60
50
40
30
20
0.0
0.2
0.4
0.6
0.8
1.0

Sman

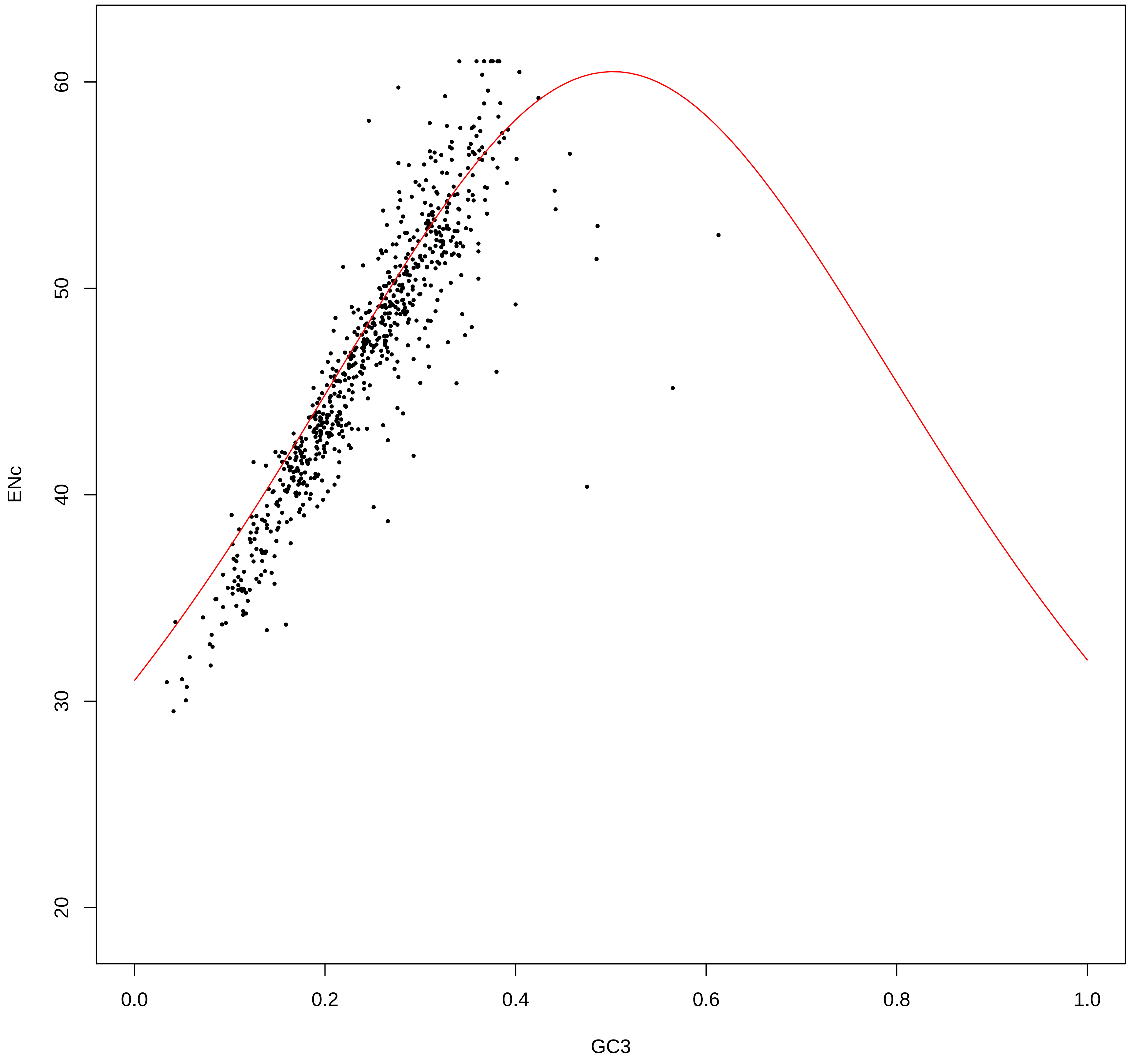

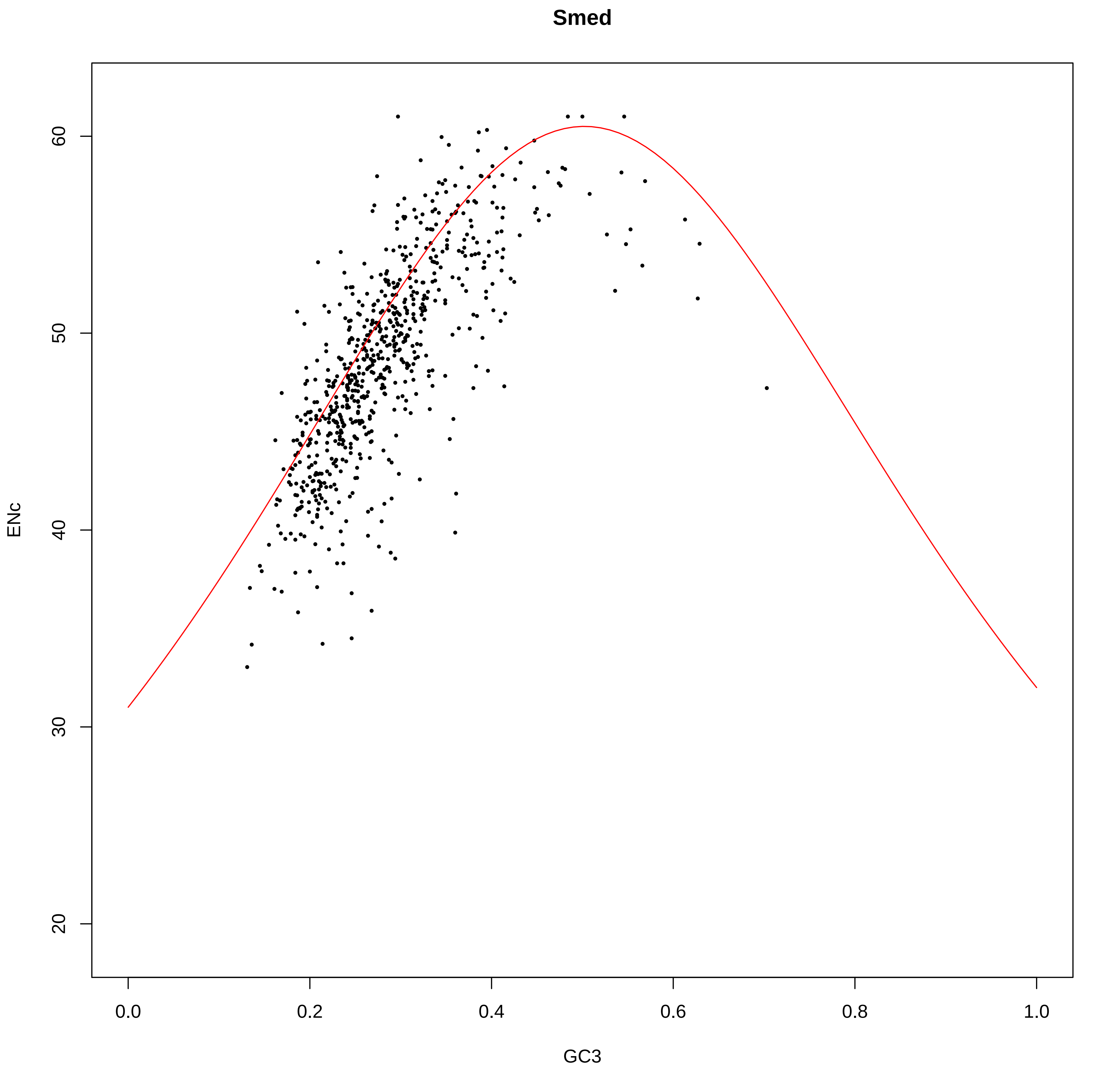

Smed
ENc
GC3
20
30
40
50
60
0.0
0.2
0.4
0.6
0.8
1.0

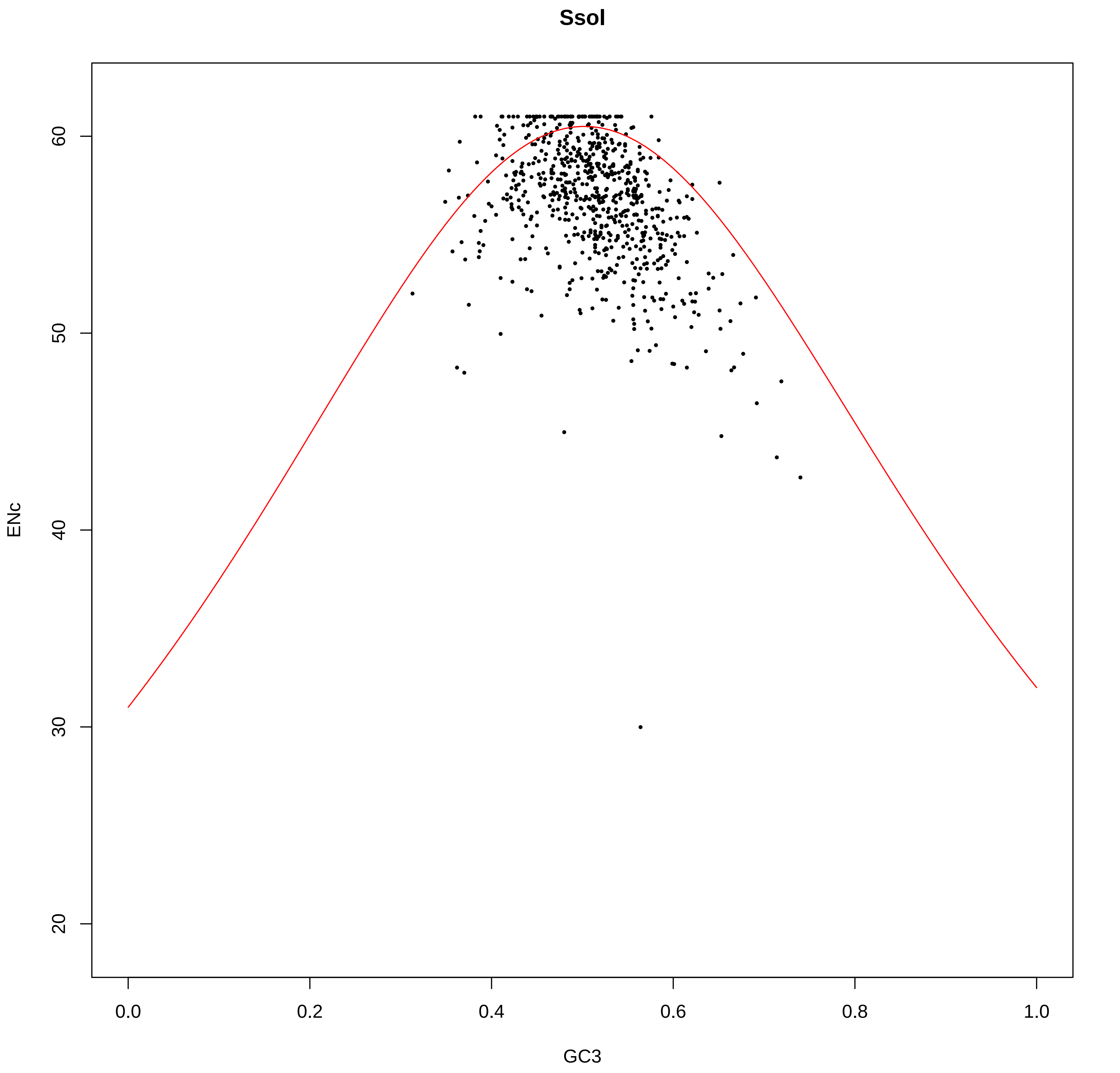
Ssol
ENc
GC3
20
30
40
50
60
0.0
0.2
0.4
0.6
0.8
1.0

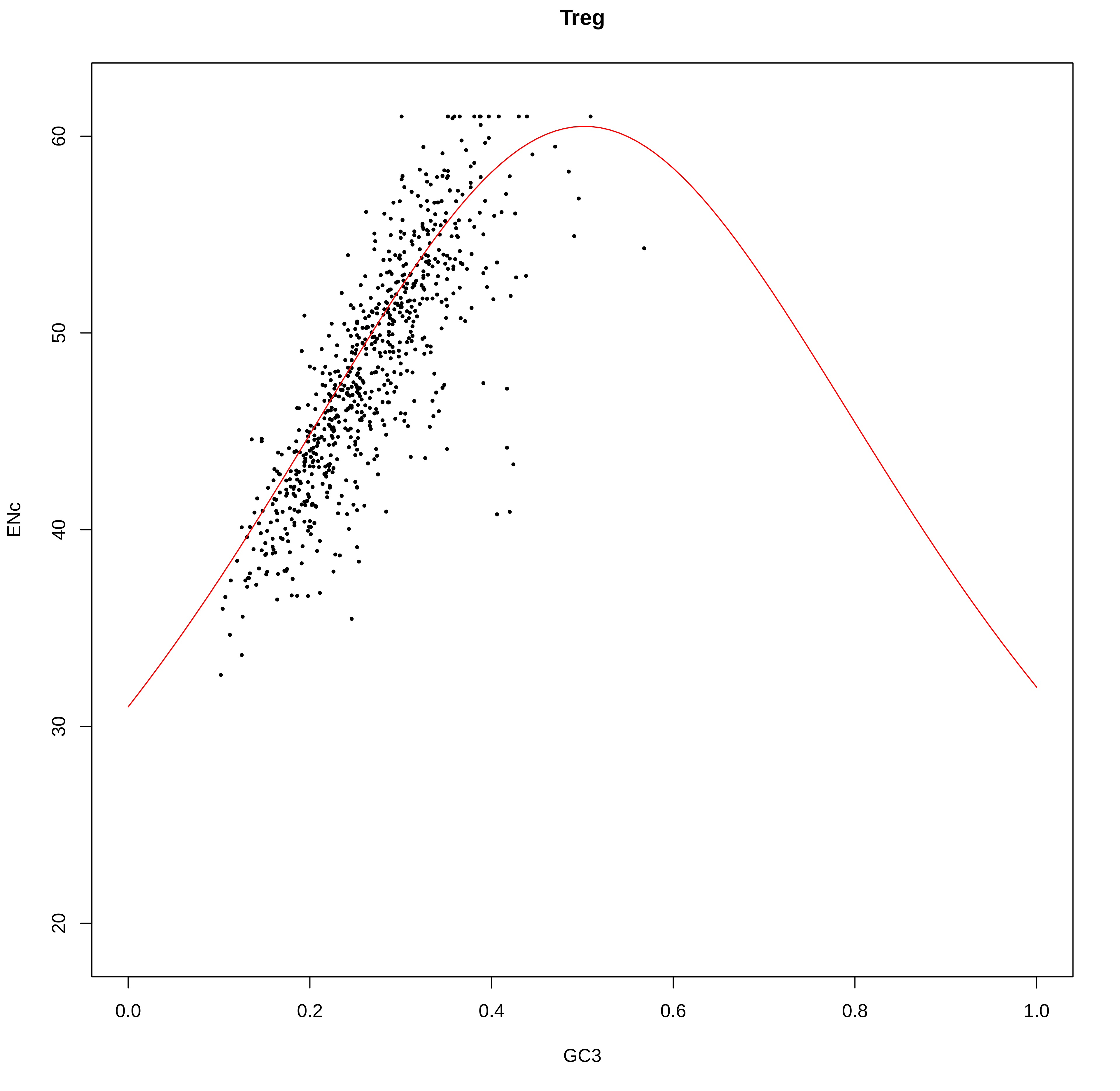

Treg
ENc
GC3
20
30
40
50
60
0.0
0.2
0.4
0.6
0.8
1.0

SFig4. Boxplots representing codon frequencies of 10% high expressed genes and 10% low expressed genes.

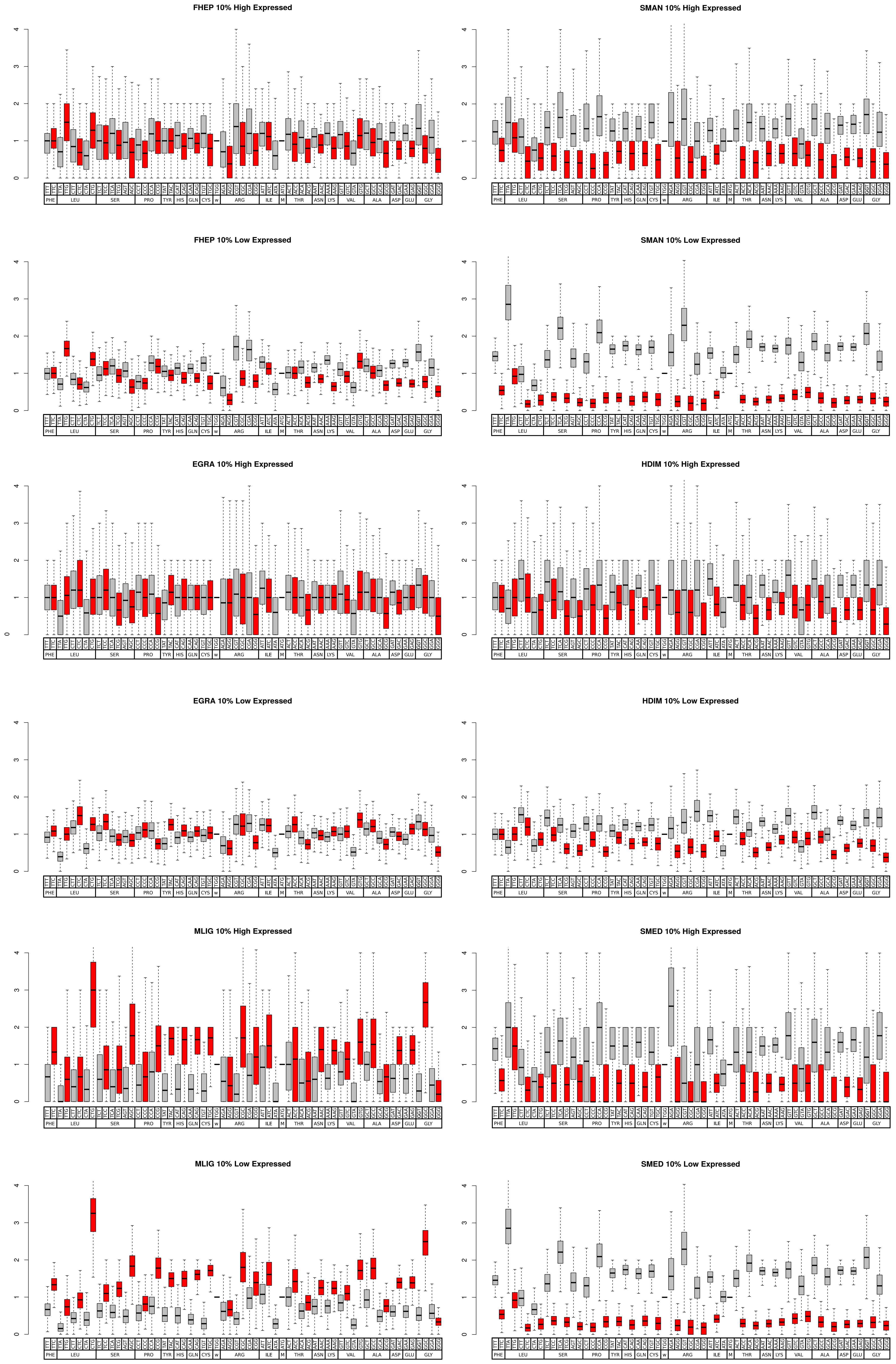
FHEP 10% High Expressed
SMAN 10% High Expressed
FHEP 10% Low Expressed
SMAN 10% Low Expressed
EGRA 10% High Expressed
HDIM 10% High Expressed
EGRA 10% Low Expressed
HDIM 10% Low Expressed
MLIG 10% High Expressed
SMED 10% High Expressed
MLIG 10% Low Expressed
SMED 10% Low Expressed
TTT TTC TTA TTG CTT CTC CTA CTG TCT TCC TCA TCG AGT AGC CCT CCC CCA CCG TAT TAC CAT CAG CAA CAG TGT TGC TGG AGA AGG CGT CGC CGA CGG ATT ATC ATA ATG ACT ACC ACA ACG AAT AAC AAA AAG GTT GTC GTA GTG GCT GCC GCA GCG GAT GAC GAA GAG GGT GGC GGA GGG
PHE LEU SER PRO TYR HIS GLN CYS W ARG ILE M THR ASN LYS VAL ALA ASP GLU GLY

SFig5. Non synonymous amino acid changes between members of three pairs of organisms of the groups trematoda (left), cestoda (center) and turbellaria (right). (A) Graphical representation of a substitution matrix of amino acids present at orthologous positions in alignments. Physicochemical properties of the residues are indicated. (B) Non-synonymous reciprocal changes that show significant differences.

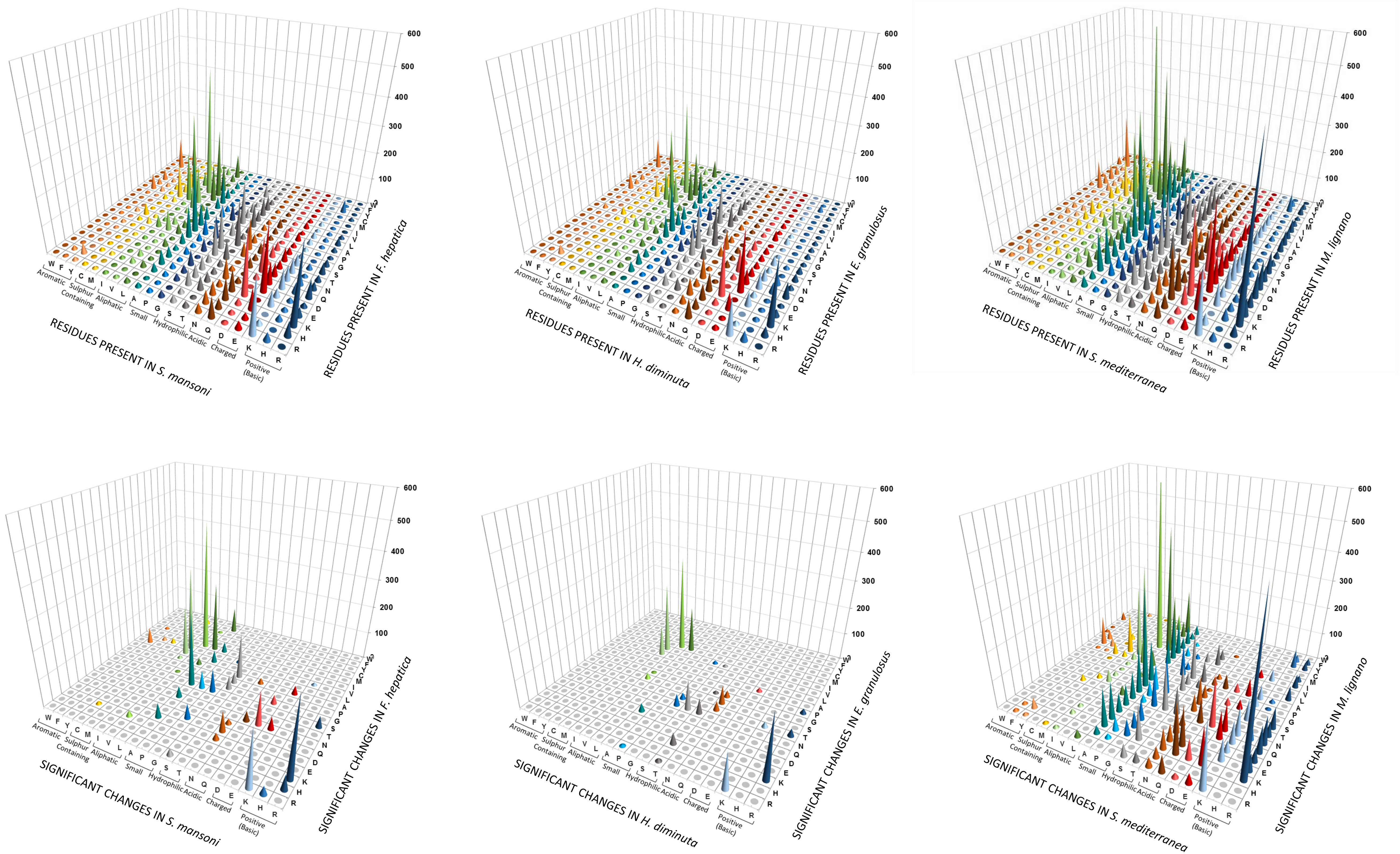
RESIDUES PRESENT IN S. mansoni
RESIDUES PRESENT IN F. hepatica
RESIDUES PRESENT IN H. diminuta
RESIDUES PRESENT IN E. granulosus
RESIDUES PRESENT IN S. mediterranea
RESIDUES PRESENT IN M. lignano
SIGNIFICANT CHANGES IN S. mansoni
SIGNIFICANT CHANGES IN F. hepatica
SIGNIFICANT CHANGES IN H. diminuta
SIGNIFICANT CHANGES IN E. granulosus
SIGNIFICANT CHANGES IN S. mediterranea
SIGNIFICANT CHANGES IN M. lignano
600
500
400
300
200
100
W F Y C M I V L A P G S T N Q D E K H R
Aromatic
Sulphur Containing
Aliphatic
Small
Hydrophilic
Acidic
Charged
Positive (Basic)

SFig6. Codon changes between *S. mediterranea* and *M. lignano*. The substitution state for each codon in pairwise alignments is represented in bars, indicating conserved codons (dark green), synonymous substitutions (pale green) and non-synonymous changes (orange). For comparison the figure is overlaid with the codon frequencies of Figure 3.

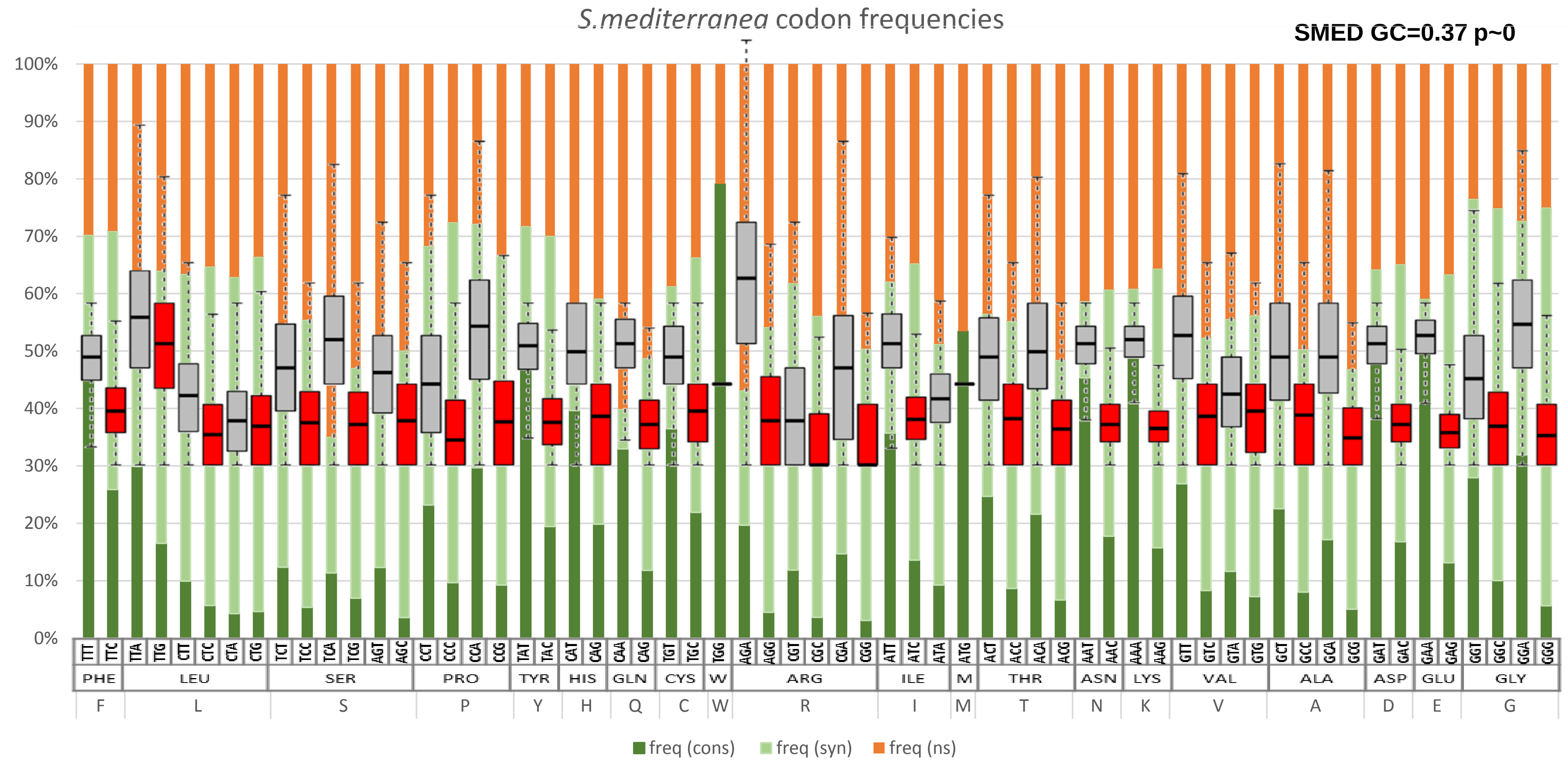
S.mediterranea codon frequencies
SMED GC=0.37 p~0
100%
90%
80%
70%
60%
50%
40%
30%
20%
10%
0%
TTT TTC TTA TTG CTT CTC CTA CTG TCT TCC TCA TCG AGT AGC CCT CCC CCA CCG TAT TAC CAT CAG CAA CAG TGT TGC TGG AGA AGG CGT CGC CGA CGG ATT ATC ATA ATG ACT ACC ACA ACG AAT AAC AAA AAG GTT GTC GTA GTG GCT GCC GCA GCG GAT GAC GAA GAG GGT GGC GGA GGG
PHE LEU SER PRO TYR HIS GLN CYS W ARG ILE M THR ASN LYS VAL ALA ASP GLU GLY
F L S P Y H Q C W R I M T N K V A D E G
freq (cons)
freq (syn)
freq (ns)

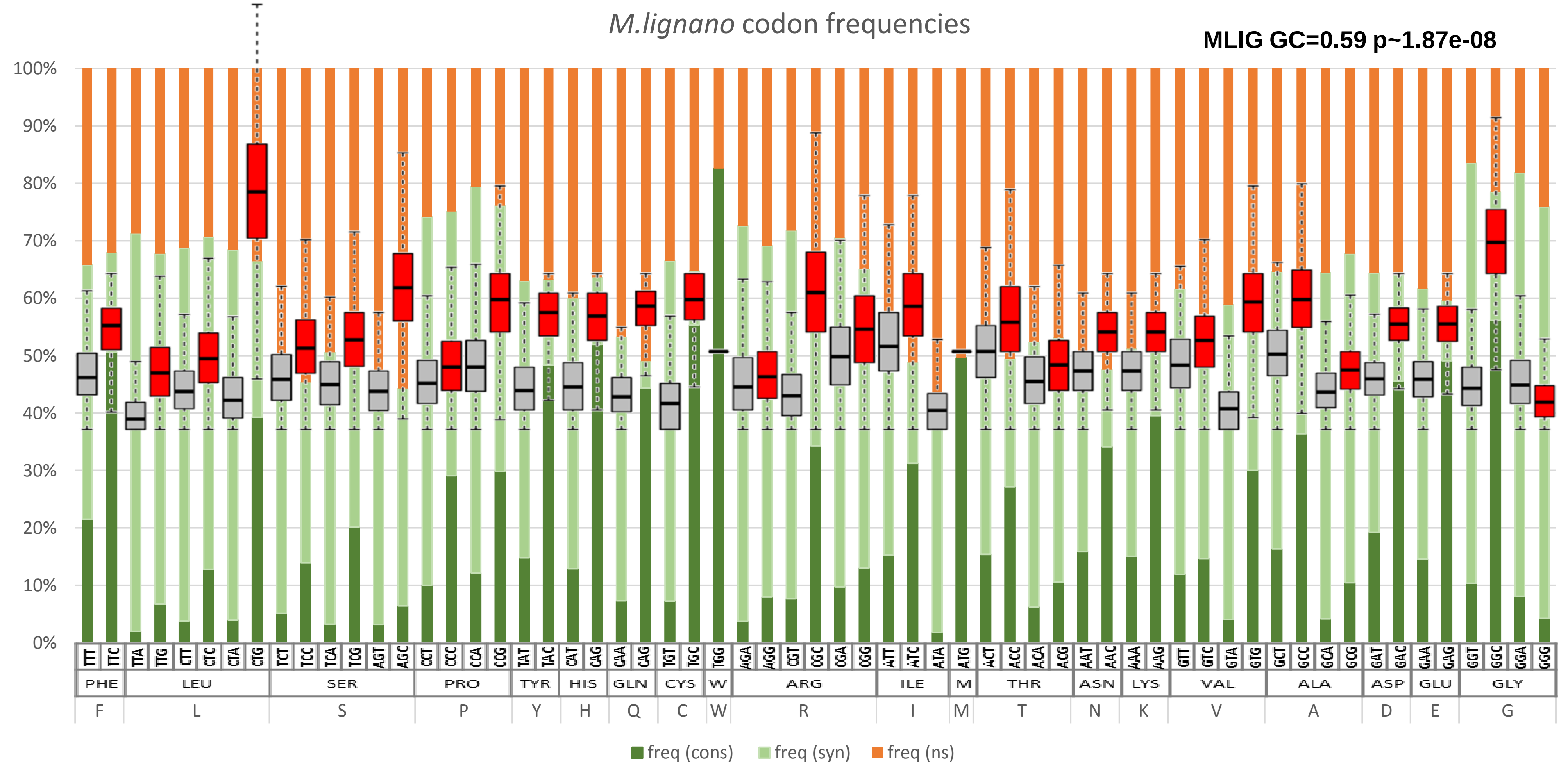
M.lignano codon frequencies
MLIG GC=0.59 p~1.87e-08
100%
90%
80%
70%
60%
50%
40%
30%
20%
10%
0%
TTT TTC TTA TTG CTT CTC CTA CTG TCT TCC TCA TCG AGT AGC CCT CCC CCA CCG TAT TAC CAT CAG CAA CAG TGT TGC TGG AGA AGG CGT CGC CGA CGG ATT ATC ATA ATG ACT ACC ACA ACG AAT AAC AAA AAG GTT GTC GTA GTG GCT GCC GCA GCG GAT GAC GAA GAG GGT GGC GGA GGG
PHE LEU SER PRO TYR HIS GLN CYS W ARG ILE M THR ASN LYS VAL ALA ASP GLU GLY
F L S P Y H Q C W R I M T N K V A D E G
freq (cons)
freq (syn)
freq (ns)

SFig7. Non synonymous changes between *S. mansoni* and *F. hepatica*. Detail of the substitution matrix of Suppl. Table 3 of the changes involving (A) Ala and Ser (*S. mansoni* codons in columns, *F. hepatica* in rows), (B) Ile and Val y (C) Arg and Lys. Note the lower than expected counts of changes towards GC3 rich codons and the enrichment in synonymous substitutions that imply two substitutions.

A

Fhep \ Sman		A GCA	A GCG	A GCC	A GCT	S TCA	S TCG	S TCC	S TCT	S AGC	S AGT
A	GCA	204	45	96	261	21	9	11	18	1	9
A	GCG	124	32	58	167	23	6	3	15	1	1
A	GCC	205	31	86	318	25	10	11	17	3	8
A	GCT	269	62	101	396	21	8	8	25	3	6
S	TCA	9	3	2	10	140	31	41	98	4	9
S	TCG	12	2	4	17	91	34	24	85	3	18
S	TCC	14	4	5	19	132	33	50	134	7	7
S	TCT	15	4	6	17	117	33	38	122	3	9
S	AGC	0	3	1	7	19	1	2	10	81	155
S	AGT	4	2	2	7	13	3	4	18	74	215

B

Fhep \ Sman		M ATG	I ATA	I ATC	I ATT	V GTA	V GTG	V GTC	V GTT
M	ATG	828	29	11	32	6	6	7	16
I	ATA	9	106	68	146	11	8	9	31
I	ATC	24	210	207	413	27	15	20	63
I	ATT	19	254	185	473	26	20	20	66
V	GTA	5	16	14	34	83	40	60	162
V	GTG	14	46	29	69	199	138	109	340
V	GTC	9	30	25	54	126	76	90	246
V	GTT	6	51	32	72	146	129	120	331

C

Fhep \ Sman		K AAA	K AAG	R CGA	R CGG	R CGC	R CGT	R AGA	R AGG
K	AAA	872	345	17	11	5	27	50	15
K	AAG	563	312	14	5	5	13	31	11
R	CGA	42	20	132	40	48	185	97	37
R	CGG	27	12	64	21	30	111	53	12
R	CGC	33	15	89	29	54	186	77	25
R	CGT	46	12	139	53	74	280	121	29
R	AGA	31	21	29	9	16	63	44	21
R	AGG	15	7	19	12	9	22	18	15
